# Demystifying the roles of single metal site and cluster in CO_2_ reduction via light and electric dual-responsive polyoxometalate-based metal-organic frameworks

**DOI:** 10.1126/sciadv.add5598

**Published:** 2022-12-09

**Authors:** Qing Huang, Qian Niu, Xiu-Fen Li, Jiang Liu, Sheng-Nan Sun, Long-Zhang Dong, Shun-Li Li, Yue-Peng Cai, Ya-Qian Lan

**Affiliations:** ^1^ School of Chemistry, South China Normal University, Guangzhou 510006, P. R. China.; ^2^ School of Chemistry and Materials Science, Nanjing Normal University, Nanjing 210023, P. R. China.

## Abstract

Photo- or electroreduction of carbon dioxide into highly valued products offers a promising strategy to achieve carbon neutrality. Here, a series of polyoxometalate-based metal-organic frameworks (M-POMOFs) were constructed by metalloporphyrins [tetrakis(4-carboxyphenyl)-porphyrin-M (M-TCPPs)] and reductive POM for photo- and electrocatalytic carbon dioxide reductions (PCR and ECR, respectively), and the mysteries between the roles of single metal site and cluster in catalysis were disclosed. Iron-POMOF exhibited an excellent selectivity (97.2%) with high methane production of 922 micromoles per gram in PCR, together with superior Faradaic efficiency for carbon dioxide to carbon monoxide (92.1%) in ECR. The underlying mechanisms were further clarified. Photogenerated electrons transferred from iron-TCPP to the POM cluster for methane generation under irradiation, while the abundant electrons flowed to the center of iron-TCPP for carbon monoxide formation under the applied electric field. The specific multielectron products generated on iron-POMOF through switching driving forces to control electron flow direction between single metal site and cluster catalysis.

## Teaser

Polyoxometalate-based MOFs were developed for photo- and electro-reduction of CO2 into highly valued products.

## INTRODUCTION

Photo- and electrocatalytic reductions of carbon dioxide (CO_2_) into serviceable energy products, also called PCR and ECR, are particularly attractive for realizing carbon recycling (*1*, *2*). The catalytic driving force can be supplied by solar energy or other renewable electricity (*3*–*5*). Although these approaches involve various pathways, their inherent nature is to break the activation energy barrier of CO_2_, which mainly relies on the choice of the catalysts (*6*). Therefore, it should be of great importance to develop high-performance photocatalyst and electrocatalyst with advantages of favorable activity, desired selectivity, and strong catalytic stability (*7*, *8*). Furthermore, structure-activity relationship is another essential parameter for rational design of previously unknow catalysts. For some of the nanocatalysts, the influence of coexistence of single metal sites and clusters in PCR and ECR processes is still hard to understand. Therefore, it is of high priority to establish a well-defined crystalline model to investigate the catalytic performance and mechanism, especially the catalytic activity and selectivity between the single metal site and the cluster.

Metal-organic frameworks (MOFs) (*9*–*11*), a kind of crystalline materials assembled by metal ions and organic linkers, have attracted broad interest in PCR and ECR because of their quasi-semiconductor characteristic, explicit structural information, and available active site (*12*–*14*). MOF catalysts have the potential to integrate single metal site, polyoxometalate (POM) cluster and the photo– and electro–dual-responsive composition. Coupled with their well-defined and highly adjustable structure, they could be conducive to providing the visualization platform to investigate the catalytic performance and mechanism in the PCR or ECR process (*15*–*18*). Using the same catalysts in both photocatalysis and electrocatalysis can be of far-reaching significance, especially for extending their efficiency of utilization. However, this is still difficult, as recent MOF catalysts mainly displays either photocatalytic or electrocatalytic activity. Therefore, it should be critical to endow the MOF catalysts with photo– and electro–dual-responsive functions. Porphyrin-based MOF catalysts, with rigid planar conjugated structure, have been reported to be capable of improving both the light absorption and electron transport capacity (*19*–*21*). However, their catalytic products are less valued, as only two electron products such as CO is produced (*22*–*24*). For further converting CO_2_ into high-value products (e.g., CH_4_, C_2_H_6_, C_2_H_4_, etc.), achieving the multiple proton-coupled electron products should be the next obstacle in developing MOF catalysts (*25*–*27*). To solve this, Cao and co-workers (*28*) and our group (*29*) merged POMs and Ni cluster into MOFs, respectively, to achieve the photocatalytic CO_2_-to-CH_4_ conversion. It supplied the idea to generate high-value products by assembling porphyrins and POM clusters into MOFs.

POMs, known as “electron sponge” (*30*), enable to enriching electrons during chemical reactions (*31*). Because of their highly soluble nature in water, forming the framework structure is necessary for protecting the POMs from being dissolved (*32*). Constructing POM-based MOFs (POMOFs) using POMs as nodes can adequately use the frame structure of the MOFs and the strong redox capacity of POMs (*33*–*35*). Therefore, POMOFs can be expected to realize multielectron and multiproton transfer, facilitating the generation of high-value reduction products.

Here, a series of crystalline POMOF catalysts were prepared to investigate their PCR and ECR performances and catalytic mechanisms through assembling the POM and tetrakis(4-carboxyphenyl)-porphyrin-M (M-TCPPs) in one framework. Theoretically, Zn-ε-Keggin, as a typical POM cluster (*36*, *37*), can contribute its abundant electrons for multielectron transfer process in the redox reactions. Meanwhile, the inherent macrocycle conjugated π electrons make the M-TCPPs effective for electron transfer in both photocatalysis and electrocatalysis. Thus, the Zn-ε-Keggin was applied to connect TCPP ligands, and the porphyrins-POM MOFs (M-POMOFs: [PMo^V^_8_Mo^VI^_4_O_35_(OH)_5_Zn^II^_4_]_2_[Fe^III^-TCPP-Cl]·Guest; [PMo^V^_8_Mo^VI^_4_O_35_(OH)_5_Zn^II^_4_]_2_[M^II^-TCPP][H_2_O]·Guest; M = Zn, Ni, Cu, Co, and Mn) were constructed. The results showed that Fe-POMOF exhibited the superior performance in both PCR reaction [CH_4_ (922 μmol g^−1^) with the selectivity of 97.2%] and ECR reaction (CO with Faradaic efficiency of 92.1%) among the six M-POMOF catalysts. Furthermore, experimental and theoretical calculation results revealed the underlying catalytic mechanisms of Fe-POMOF in PCR and ECR reactions, respectively. In PCR, it can be speculated that the photogenerated electrons flowed from the Fe-TCPP to the POM for CH_4_ generation. In contrast, abundant electrons flowed to the center of Fe-TCPP for CO formation under the applied electric field. Thus, this work can be of great importance for understanding not only the electron transfer mechanism under the photo- and electrocatalytic driving forces but also the interaction between the single metal site and the POM cluster. It also provides the emerging idea and valuable method for developing highly effective catalysts.

## RESULTS

### Structure and characterization

**Fig. 1.**
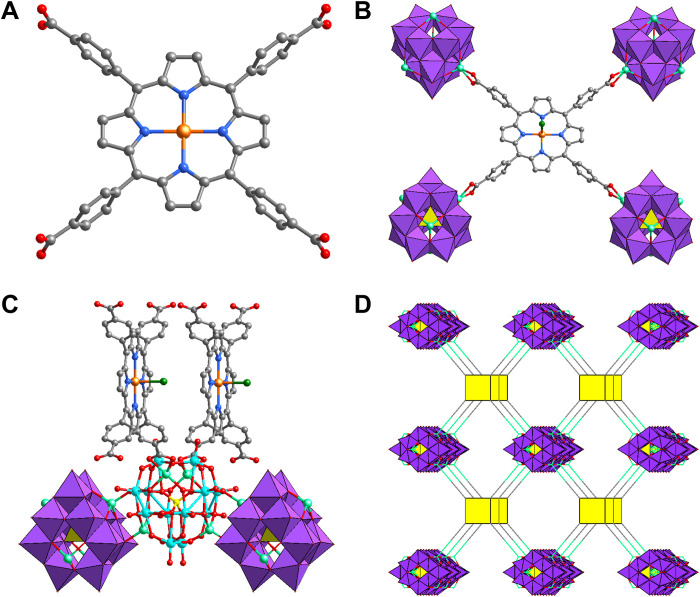
The crystal structures of M-POMOFs. (**A**) M-TCPP (M = Fe^III^, Zn^II,^ Ni^II^, Cu^II^, Co^II^, and Mn^II^) linkers. When the M is Fe, it also coordinated by a Cl ion. Color code: C, black; N, blue; O, red; P, yellow; Cl or O, dark green; Mo, lake blue; Fe, golden; Zn, light green; (**B**) Coordination environments of Fe-TCPP ligand. (**C**) Coordination environments of the Zn-ε-Keggin cluster. (**D**) Three-dimensional frameworks of M-POMOFs.

The block-shaped M-POMOF crystals were successfully synthesized via the hydrothermal method (fig. S1). The detailed procedure and materials characterizations are shown in the Supplementary Materials. After assembling reductive Zn-ε-Keggin and six different M-TCPPs, the M-POMOFs exhibited a nearly identical framework structure, adopting the orthorhombic *Fmmm* space group confirmed by single-crystal x-ray diffraction (XRD) analyses. In the porphyrin center, Fe(III) coordinates to four N atoms and one disordered Cl atom, while other metals (Co^II^, Ni^II^, Zn^II^, Mn^II^, and Cu^II^) coordinates to four N atoms and one disordered O atom from H_2_O. Each Zn-ε-Keggin segment connects to two M-TCPP ligands and two Zn-ε-Keggin fragments, while each M-TCPP coordinates with four Zn-ε-Keggin segments simultaneously (Fig. 1 and fig. S2). Among the above coordination modes, the adjacent Zn-ε-Keggin units interconnect and form the Z-shaped (εZn)∞ chains along the crystallographic *c* axis (fig. S3), which are further bridged by two carboxylates of the M-TCPP linker to complete the formation of a defined three-dimensional framework.

The high purities of M-POMOF samples were confirmed by comparison of their powder XRD (PXRD) patterns for the simulated and as-synthesized samples (fig. S4). After soaking in both acid and basic solutions (pH 3 to 11) for 24 hours, the unchanged PXRD patterns of the POMOFs implied their high chemical stability (fig. S4). Thermogravimetric analyses were recorded to test the thermal stability of these samples, indicating that they were thermally stable to about 300°C. A continuous weight loss step from 300° to 500°C was observed, revealing the thermal decomposition processes. The good stability of M-POMOF was mainly due to the twofold interpenetrating structure, which stabilizes the two networks. In addition, the structure synthesized via high-temperature (180°C) hydrothermal process was more stable (*33*, *36*). The good pH stability and thermal stability endow these materials with high potential for photocatalysis and electrocatalysis.

**Fig. 2.**
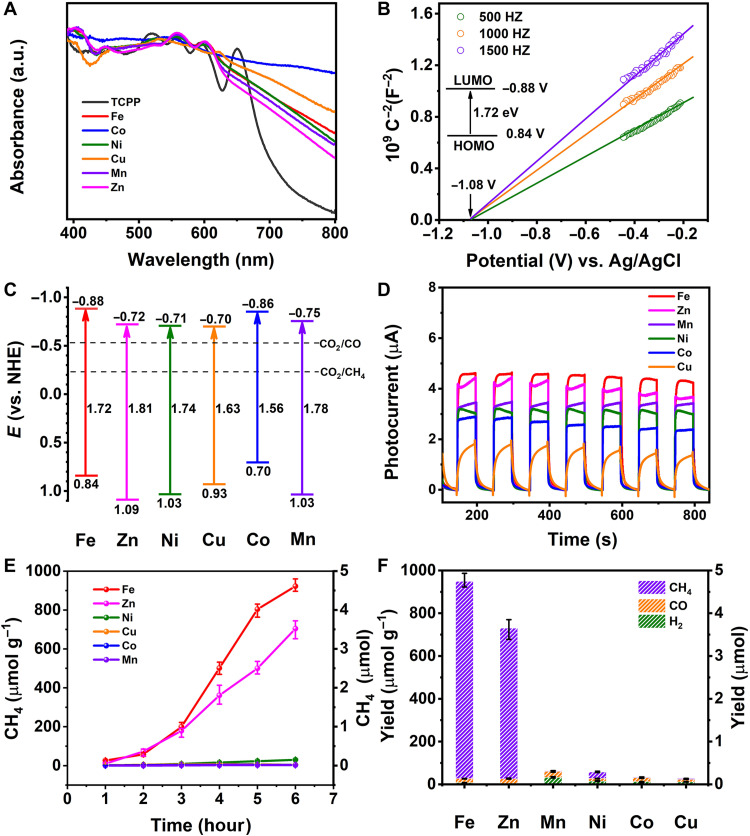
Photocatalytic performances of M-POMOFs. (**A**) UV-visible diffuse reflection spectra for six M-POMOFs. (**B**) Mott-Schottky plots with the inset of the bandgap of its conduction and valence band for Fe-POMOF. (**C**) The bandgaps of M-POMOFs. (**D**) Transient photocurrent curves. (**E**) The amounts of CH_4_ produced as a function of the visible illumination time. (**F**) The production yield of CH_4_, CO, and H_2_ in PCR after 6 hours. a.u., arbitrary units.

In the photochemical characterization, the M-POMOF crystals showed a wide range of absorption in the visible region, inheriting the feature of the TCPP ligand (Fig. 2A). Ultraviolet (UV)–visible diffuse reflection spectra revealed that the TCPP ligand could be capable of serving as a visible light harvesting unit. The bandgaps of M-POMOFs were measured to be ~1.7 to 1.9 eV (fig. S6). Mott-Schottky patterns showed that these M-POMOFs belonged to n-type semiconductors based on the positive slope, indicating the potentials for supplying electrons in photocatalysis (Fig. 2B and figs. S7 to S11). As shown in Fig. 2B and figs. S7 to S11, the bottom of the conduction band values of M-POMOFs were estimated to be −0.88 V (Fe), −0.72 V (Zn), −0.71 V (Ni), −0.70 V (Cu), −0.86 V (Co), and −0.75 V (Mn) versus normal hydrogen electrode (versus NHE). These values are much lower than the reduction potential of CO_2_-CH_4_ (−0.24 V) and CO_2_-CO (−0.52 V) (*8*), implying the theoretical potentials for converting CO_2_ into CH_4_ and CO (Fig. 2C). The *I*-*t* curves of M-POMOFs, especially the Fe-POMOF and Zn-POMOF, exhibited remarkable responsiveness and repeatability under visible light irradiation (Fig. 2D).

### The PCR performance of M-POMOFs

The visible light–driven photoreduction were carried out in the triethanolamine (TEOA) with deionized water under 293 K and 1 atm of CO_2_ according to the semiconductor properties, redox properties, and rich active metal sites. The peaks of CH_4_ and CO were observed by gas chromatography (GC) analysis (fig. S12), while no peak of liquid production was found in ^1^H nuclear magnetic resonance (NMR) (fig. S13). It indicated that the main reduction products of CO_2_ photoreduction were CH_4_ and CO rather than the liquid product. The results showed that the CH_4_ production decreased in an order of Fe (922 μmol g^−1^) > Zn (704 μmol g^−1^) ≫ Ni (29.9 μmol g^−1^) > Cu (3.8 μmol g^−1^) > Co (3 μmol g^−1^) ≈ Mn (2.9 μmol g^−1^) (Fig. 2, E and F). The selectivity of M-POMOFs decreased in a sequence of Fe (97.2%) > Zn (96.6%) ≫ Ni (52.0%) > Cu (14.2%) > Co (8.8%) > Mn (4.5%) (Fig. 2, E and F). Notably, the selectivity of Fe-POMOF for CH_4_ reached to 97.2%. With this high selectivity, Fe-POMOF continuously produced CH_4_ (922 μmol g^−1^) along with CO (19 μmol g^−1^) and H_2_ (8 μmol g^−1^) during the 6-hour reaction (figs. S14 and 15). This confirmed the superior CO_2_-CH_4_ reduction with both high CH_4_ production and selectivity in Fe-POMOF. Besides that, the performance of Fe-POMOF for CH_4_ generation far exceeded those of other MOF-based materials (table S1) (*24*, *28*, *38*–*42*). Table S2 illustrated that all the factors including visible light absorption, the reaction substrate (CO_2_), TEOA, and catalyst were indispensable in continuously producing CH_4_ during a prolonged time. In addition, because ^13^CH_4_ [mass/charge ratio (*m*/*z*) = 17] and ^13^CO (*m*/*z* = 29) were detected in ^13^CO_2_ isotopic experiments (figs. S16 and S17), it could be evident that CO_2_ is the main PCR reactant.

To explore the differences in photocatalytic effects of six different M-POMOFs, charge separation efficiency has been considered as the key characteristic of investigation. Transient photocurrent curves and transient-state photoluminescence (TSPL) spectroscopy were recorded to obtain the photogenerated electron-hole separation efficiency. The transient photocurrent curves of M-POMOFs exhibited remarkable responsiveness in the order of Fe > Zn > Mn > Ni > Co > Cu, which indicated that Fe-POMOF has the strongest charge separation efficiency (Fig. 2D) (*43*). TSPL spectrum provided important information about the carrier lifetime, which represents the key index of radiation recombination efficiency of photogenerated charge. As can be seen from fig. S18, the carrier lifetimes of M-POMOFs were determined to be 3.26 ns (Fe), 3.06 ns (Zn), 3.01 ns (Mn), 3.0 ns (Ni), 2.84 ns (Co), and 2.75 ns (Cu). Therefore, Fe-POMOF exhibited the longest lifetime of photogenerated charge carriers among all the M-POMOFs. As shown in fig. S18, the carrier lifetimes of M-POMOFs were determined to be 3.26 ns (Fe), 3.06 ns (Zn), 3.01 ns (Mn), 3.0 ns (Ni), 2.84 ns (Co), and 2.75 ns (Cu). In addition, the carrier lifetimes of M-TCPP ligands were determined to be 2.80 ns (Fe), 2.79 ns (Zn), 2.72 ns (Mn), 2.66 ns (Ni), 2.51 ns (Co), and 2.47 ns (Cu), respectively (fig. S19). Compared to the TCPP ligand, the M-POMOF structure increased the distance of the electron transfer path, leading to an increase in the carrier lifetime. Second, the long-range ordered structure of MOF, acted as an electron transport channel, was beneficial to enhance the transport of photogenerated electrons and slow down the recombination of photogenerated electron-hole pairs (*44*). Thus, M-POMOF displayed a longer carrier lifetime than corresponding M-TCPP, certifying that the assembled M-POMOF could have benefits in prolonging the carrier lifetime.

The recycling stability tests showed the excellent catalytic activity (>865 μmol g^−1^) and selectivity (>97%) for Fe-POMOF throughout the whole six cycles (fig. S20). For Fe-POMOF catalyst, a range of different characterization techniques were applied to confirm its stability after the PCR reaction. PXRD spectra confirmed that Fe-POMOF maintained the stability of the framework structure after catalytic reactions (fig. S21). Raman spectra were consistent before and after PCR reaction (fig. S22). Fourier-transform infrared (FTIR) spectroscopy revealed that no noticeable change of the peaks for the function groups of Fe-POMOF (fig. S23). X-ray photoelectron spectroscopy (XPS) certified consistent valence states of metallic elements in Fe-POMOF (fig. S24). According to the results of metal K-edge x-ray absorption near-edge structure for the catalysts before and after PCR reaction, the valence states of metals kept consistent (fig. S25). The extended x-ray absorption fine structure (EXAFS) data and wavelet transform EXAFS analysis showed no change of the coordination environment, coordination number, and bond length around metal after PCR reaction (fig. S25 and tables S3 and S4), illustrating a good stability maintained by the Fe-POMOF catalyst in PCR reaction. Above various characterization results showed that Fe-POMOF catalyst maintained a good stability in PCR reaction.

### The ECR performance of M-POMOFs

**Fig. 3.**
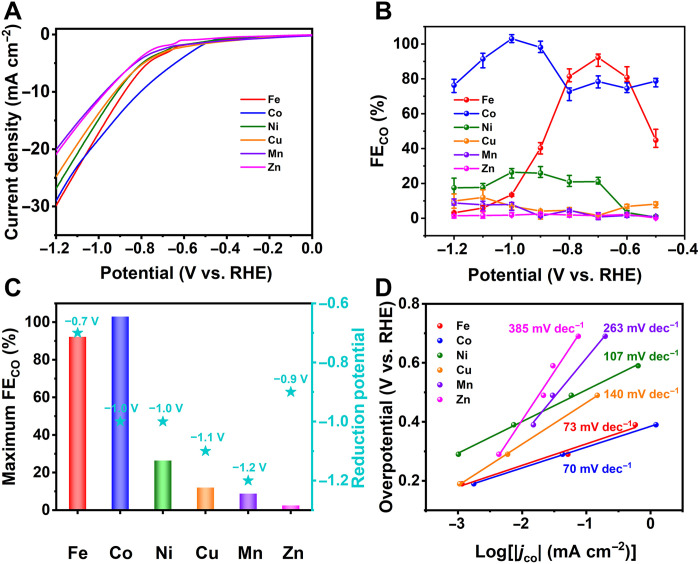
Electrocatalytic performances of M-POMOFs. (**A**) Linear sweep voltammetric curves were performed in 0.5 M KHCO_3_ electrolyte with the saturated CO_2_ atmosphere (99.999%). (**B**) FE_CO_. (**C**) Maximum FE_CO_. (**D**) Tafel slopes in the CO_2_-saturated 0.5 M KHCO_3_ aqueous solution..

In the electrocatalysis, linear sweep voltammetry (LSV) experiments were applied to evaluate the electrocatalytic activity of M-POMOFs. CO_2_-saturated 0.5 M KHCO_3_ solution showed both more positive initial potential and larger current density than those of the Ar-saturated aqueous solution, indicating that these M-POMOFs were capable of CO_2_ reduction in electrocatalysis (fig. S26). Under −1.2 V versus reversible hydrogen electrode (versus RHE), Fe- and Co-POMOFs showed larger current densities (30.1 and 29.2 mA cm^−2^) than those of other four M-POMOFs [Ni (27.1 mA cm^−2^), Cu (25.0 mA cm^−2^), Mn (20.2 mA cm^−2^), and Zn (21.0 mA cm^−2^)] (Fig. 3A). In addition, the current densities of Fe- and Co-POMOF were much larger than those of many other reported MOF materials (table S5).

CO and H_2_ were lastly found for the main products in the electrocatalysis (fig. S27). No liquid product was generated in the solution after the ECR reaction (fig. S28). The selectivity of M-POMOFs was assessed by the Faradaic efficiency of CO and H_2_ (FE_CO_ and FE_H2_), with the results recorded between −0.5 and −1.2 V (Fig. 3B and fig. S29). The results showed that the maximum FE_CO_ values of M-POMOFs were more than 99% (Co, −1.0 V), 92.1% (Fe, −0.7 V), 26.4% (Ni, −1.0 V), 12% (Cu, −1.1 V), 8.8% (Mn, −1.2 V), and 2.5% (Zn, −0.9 V), respectively (Fig. 3C). Among which, Fe- and Co-POMOF exhibited superior ECR performance compared to that of many other MOFs (table S6). Although Co-POMOF exhibited high conversion efficiency for CO generation, more positive reduction potentials implied less energy required for proceeding the catalytic reactions. Thus, taking the potentials into consideration, the FE_CO_ of Fe-POMOF reached 92.1% at −0.7 V, higher than those of other M-POMOFs (78.4% for Co, 21.0% for Ni, 1.6% for Cu, 0.72% for Mn, and 1.5% for Zn) (Fig. 3B). The ECR performance originated from the catalysts rather than the conductive substrate, because no CO was detected on substrate (fig. S30). For ensuring the source of CO, Ar atmosphere and ^13^CO_2_ isotopic experiments were carried out. Ar atmosphere confirmed that no CO generated in the control experiments (fig. S31). Furthermore, the ^13^CO_2_ isotopic tracing results indicated that CO was mainly derived from the CO_2_ (fig. S32).

Partial current densities, as another crucial parameter in ECR, were also calculated for all the M-POMOFs. Fe-POMOF and Co-POMOF gave higher partial CO current density (*j*_CO_) than partial H_2_ current density (*j*_H2_) at more positive reduction potential (figs. S33 and S34), indicating that the improved current was more efficient in reducing CO_2_ than H_2_O. On the condition of same potentials (−0.7 V), the |*j*_CO_| of Fe- and Co-POMOFs were 3.2 and 5.2 mA cm^−2^, respectively, far superior to other four M-POMOFs (fig. S31). In addition, the CO turnover frequency of Fe-POMOF and Co-POMOF could reach 301 and 488 hour^−1^ at −0.7 V (fig. S35), respectively. Both of the results indicated a higher capacity of Fe-POMOF and Co-POMOF than the other POMOF catalysts in the electrocatalysis.

Tafel slopes are generally applied to describe the dynamics activity of electrical catalysts. In this study, the values of Fe-POMOF and Co-POMOF were 73 and 70 mV decade^−1^, an order of magnitude smaller than those of Ni (107 mV decade^−1^), Cu (140 mV decade^−1^), Mn (263 mV decade^−1^), and Zn (385 mV decade^−1^). With more efficient charge transfer and larger active surface, remarkable reaction kinetics were found in Fe-POMOF and Co-POMOF for CO formation (Fig. 3D). The double-layer capacitance (*C*_dl_) values of M-POMOFs decreased in an order of Co (30.87 mF cm^−2^) > Fe (28.55 mF cm^−2^) > Ni (27.00 mF cm^−2^) > Cu (25.75 mF cm^−2^) > Mn (23.03 mF cm^−2^) > Zn (16.21 mF cm^−2^), indicating that Fe-POMOF and Co-POMOF can offer more active sites for promoting the CO_2_ reduction speed (fig. S36). The Nyquist plots showed that the charge transfer resistances of Fe-POMOF and Co-POMOF were much lower than those of other four M-POMOFs (fig. S37), suggesting that a faster charge shuttle speed in Fe-POMOF and Co-POMOF transferred from the catalysts to the reactants, resulting in enhanced both activity and selectivity of the electrochemical reduction of CO_2_ to CO.

In view of that Fe-POMOF exhibited excellent performance in both PCR and ECR, Fe-POMOF had the potential to act as light and electric dual-responsive catalysts. To further confirm this, the durability in ECR of Fe-POMOF was assessed as the high stability that had been certified in PCR section. As shown in fig. S38, the PXRD after the reaction revealed that the structure of the framework remained unchanged. The Raman spectra were consistent before and after the reaction (fig. S39). Meanwhile, the current stability could be as long as 32 hours with the current density stabilizing at 5.7 mA cm^−2^ (fig. S40). FE_CO_ stability remained at the region of 86 to 92% before 12 hours. The above experiments had demonstrated the good stability of Fe-POMOF in the ECR reaction. For XPS determination, it was further applied to characterize the variation of metal valence state in the Fe-POMOF catalysts. XPS certified consistent valence states of Mo and Zn elements in Fe-POMOF before and after ECR reaction (fig. S41). No Fe signal in XPS could be explained by the insufficient mass of Fe in Fe-POMOF. After we confirmed the good stability of the framework, we further confirmed the valence of Fe in the Fe-TCPP before and after the ECR reaction, which reflected valence variation of Fe in Fe-POMOF due to the same environment of Fe. As shown in the results (fig. S42), the valence state of Fe(III) remained the same before and after the ECR reaction.

To reveal the role and function of Fe-TCPP and POM in ECR reaction, the separate POM and Fe-TCPP were investigated to explore their roles in Fe-POMOF structure. The results showed that almost no CO is generated on POM but FE_CO_ of Fe-TCPP reached 50% at −0.7 V (fig. S43). Thus, Fe-TCPP should be the contributor (catalytic site) for the ECR reaction. At this time, POM, as an electronic concentrator, was expected to accept and transfer the external electrons from the electric field to the M-TCPP active center. For investigating the POM’s role in ECR, porphyrin MOFs [Zr-PCN-222 (Fe) and Hf-PCN-222 (Fe) (*45*)] without the POM units were used as comparison examples. The results showed a sequential decrease in ECR selectivity in Fe-POMOF (~92%), Hf-PCN-222 (Fe; ~70%), Zr-PCN-222 (Fe; ~65%), and Fe-TCPP (~50%) under the same conditions (−0.7 V) (fig. S44). It suggested that POM in Fe-POMOF exhibited strong ability in enriching and transferring electrons to metalloporphyrin center.

### In situ characterization

**Fig. 4.**
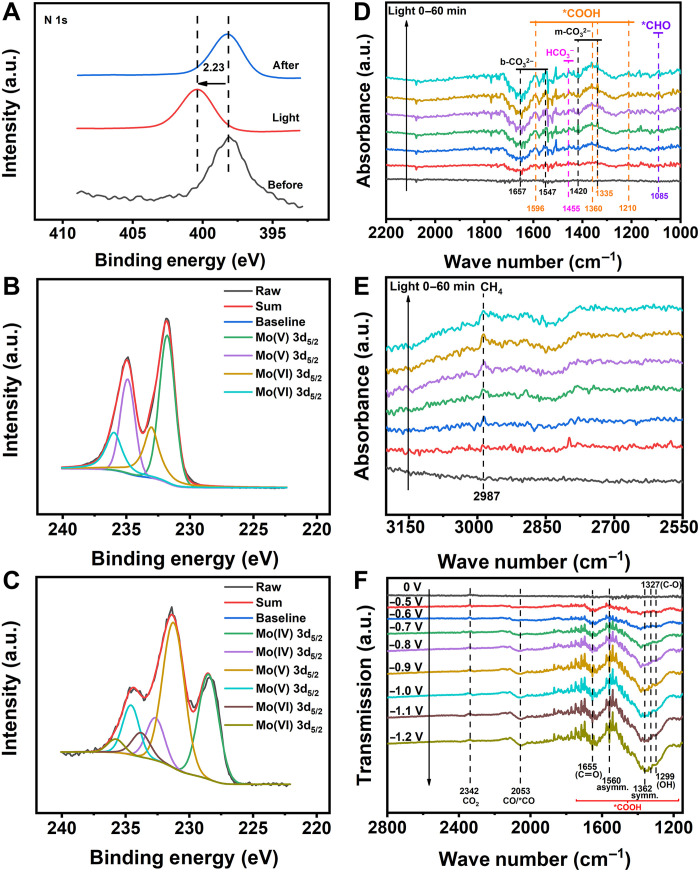
In situ characterization experiments of Fe-POMOF. (**A**) In situ XPS signals of N 1s for Fe-POMOF in PCR reaction. (**B** and **C**) In situ XPS signals of Mo 3d before PCR reaction and under visible light were recorded in (B) and (C). In situ XPS signals of Mo 3d after PCR reaction in the Supplementary Materials. (**D** and **E**) In situ FTIR spectra during PCR reaction were recorded in (D) and (E) via the absorbance method. (**F**) In situ FTIR spectra during ECR reaction via the transmission method.

For further finding the catalytic mechanism in both PCR and ECR, in situ XPS and in situ FTIR were conducted. The flow direction of photogenerated electron in Fe-POMOF was detected by in situ XPS. Under visible light, the binding energy of characteristic element N of Fe-TCPP increased (Fig. 4A), while that of Mo of POM decreased simultaneously (Fig. 4, B and C, and fig. S45). The binding energy of Fe moved to the direction of low value after light irradiation, which was evaluated on the basis of the in situ XPS data for Fe-TCPP (fig. S46). Although binding energy of Fe changes slightly, it does not affect the overall increase of binding energy in whole Fe-TCPP ligand of Fe-POMOF. In detail, the binding energy of N moved to the direction of high binding energy about 2.23 eV, along with slight change of Fe, and parts of Mo(V) and Mo(VI) were reduced to Mo(IV). This phenomenon might be attributed to the fact that the electrons were lost from the Fe-TCPP ligand and captured by the POM in Fe-POMOF. Above these phenomena might be attributed to the fact that the electron was released from the TCPP ligand and captured by the POM in Fe-POMOF. It meant that photogenerated electrons flowed from Fe-TCPP to POM during the photocatalytic reaction and the active center was more likely to be the POM in Fe-POMOF in the PCR processes. After the PCR, the state of catalysts was restored to the state before the reaction (Fig. 4, A to C, and fig. S45).

Furthermore, in situ FTIR measurements were used to probe adsorbed intermediates and verify the reactive sites during the PCR and ECR reactions. In PCR reaction, in situ FTIR results revealed that CO_2_ could be successfully activated into *COOH on catalysts in both visible light and dark environments (Fig. 4D and fig. S47). In contrast to the dark conditions, the occurrence of more pronounced peaks in visible light indicated that the visible light could provide more energy for adsorbing and activating CO_2_ (Fig. 4D). The new IR peaks at 1547 and 1657 cm^−1^ were attributed to the bidentate carbonate (b-CO_3_^2−^) species on Fe-POMOF. Peaks at 1335 and 1420 cm^−1^ corresponded to monodentate carbonate (m-CO_3_^2−^). The peak at 1455 cm^−1^ was assigned to HCO_3_^−^ (*46*–*48*). Peaks appearing at 1210, 1335, 1360, and 1596 cm^−1^ were ascribed to the formation of *COOH, which was a crucial intermediate during the photochemical conversion of CO_2_ to CH_4_ or CO (*49*, *50*). The IR peak at 1085 cm^−1^ could be assigned to *CHO, another pivotal intermediate species for the formation of CH_4_ (*47*, *50*). Besides, the peak of CH_4_ on the catalyst appeared at 2987 cm^−1^ (Fig. 4E) (*51*). The detection of the key intermediates and products implied the whole progress of the photochemical CO_2_ conversion, which is summarized in supporting material 1 for reaction path (RS1) to RS13 in theoretical calculations. To further confirm the catalytic site, the individual POM and Fe-TCPP were also applied to conduct the in situ FTIR experiments under the photocatalysis. An obvious peak of CH_4_ occurred on the POM rather than on Fe-TCPP, indicating that POM of Fe-POMOF acted as the active site for CO_2_-CH_4_ conversion in PCR reaction (figs. S48 and S49). In the ECR, the in situ FTIR experiments of Fe-POMOF were carried out at a potential ranging from −0.5 to −1.2 V (Fig. 4F). A significant amount of CO (*CO) appeared in the band at 2053 cm^−1^, and the CO was accumulated with the consumption of CO_2_ (peak at 2342 cm^−1^) (*52*). Meanwhile, symmetric (ν_s_) and asymmetric (ν_as_) stretching vibrations of *COOH were observed at 1384 and 1560 cm^−1^. OH deformation and C═O stretch vibration of *COOH were recorded at 1299 and 1655 cm^−1^ (*53*). The key intermediate (*COOH) for the CO formation was observed in ECR. Furthermore, individual POM and Fe-TCPP were performed in ECR. In situ FTIR results indicated that Fe-TCPP, rather than the POM, could activate the CO_2_ molecular for CO generation under the electrocatalysis (figs. S50 and S51). Thus, Fe-TCPP of Fe-POMOF was more likely to be the active catalytic site for CO_2_-CO conversion in ECR reaction.

### Density functional theory calculations

**Fig. 5.**
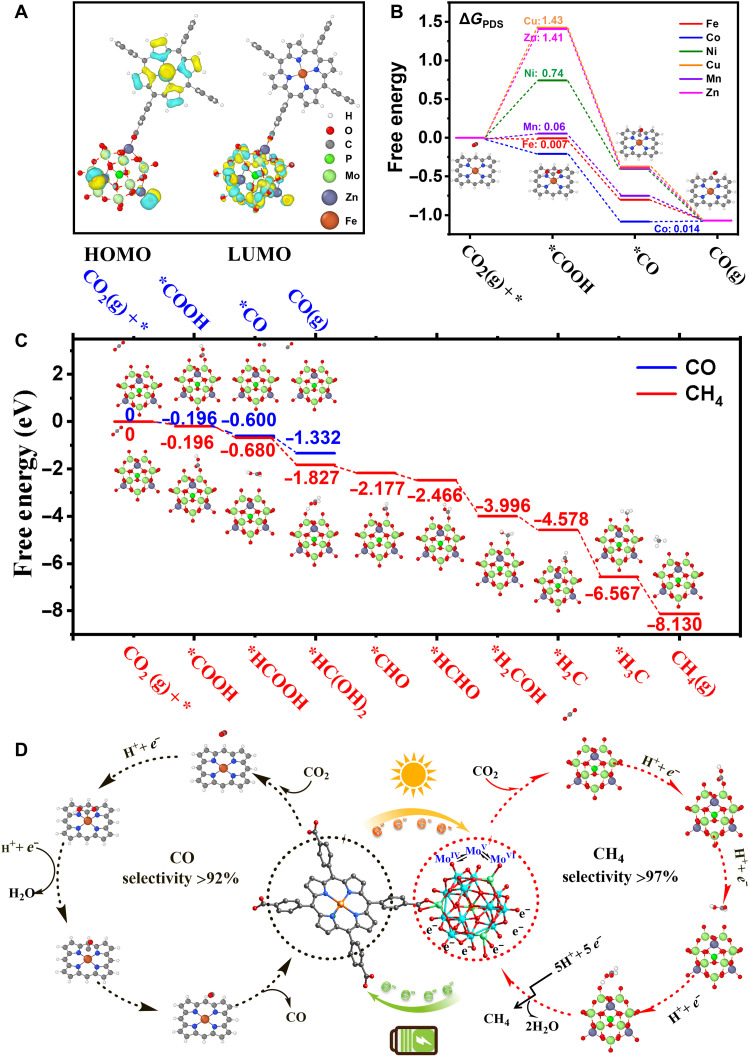
The DFT calculation and proposed reaction mechanism. (**A**) The charge density of LUMO and HOMO was calculated for Fe-POMOF. (**B**) Calculated Gibbs free energy profile for ECR reaction toward the production of CO on M-TCPP of M-POMOF. (**C**) Simulated CO_2_-to-CH_4_ and CO_2_-to-CO conversion pathway with Gibbs free energy on POM in Fe-POMOF under photocatalysis condition. (**D**) Proposed reaction mechanism in PCR and ECR reactions for Fe-POMOF, respectively.

To verify the experimental results, density functional theory (DFT) analysis was conducted to describe the photoreduction and electroreduction in Fe-POMOF. For PCR reaction, it could be seen that the highest and lowest occupied molecular orbitals (HOMO and LUMO, respectively) were mainly located in Fe-TCPP and the POM cluster, respectively (Fig. 5A and figs. S52 to S55). Above results indicated the POM cluster could act as the active sites for photoreduction (since once Fe-POMOF was stimulated by light, the POM would be the electron acceptor). In light of this, the free energy diagram (FED) for the POM was calculated under the critical potential of −0.88 V (Fig. 5C). The second step (*COOH→*CO/*HCOOH) in Fig. 5C was the key process in determining the products of CO_2_ reduction, which was based on the value of Gibbs energies (Fig. 5C). Because of Δ*G*^CH4^_2_ (−0.484 eV) < Δ*G*^CO^_2_ (−0.404 eV), it could be inferred that the POM preferred CH_4_ pathway than CO in the photocatalysis (Fig. 5C and fig. S56). Because the POM was the active site for photocatalysis, CH_4_ was hence the main product of photoreduction. To explain why Fe-POMOF outperformed other metal counterparts, the associated HOMO and LUMO of the other M-TCPP were plotted in fig. S57. Intrinsically, for photocatalysis, the so-called “performance” is made of two factors. They are the catalytic activity and the light absorption efficiency. For all the metal POMOFs, the LUMO orbital is contributed by the POM (fig. S57). Note that for Mn and Cu, the M-TCPP also has the contribution for LUMO. Because the adsorption of *COOH on M-TCPP is stronger than that on the POM, the POM should remain as the catalytic active site for CO_2_ reduction reaction (CO_2_RR), and the catalytic activities should differ little between each metal. Therefore, the performance should be mainly attributed to the light absorption efficiency. For this, as indicated by the energy level diagrams of the frontier orbit (fig. S57), Fe-POMOF has the smallest LUMO-HOMO gap among all the metals. Meanwhile, the experiments also suggest that the energy gap of Fe-POMOFs is one of the smallest. This indicates the higher light absorption efficiency of Fe than other metals. On the other hand, the Bader charge analysis indicates that Fe-TCPP has a relatively large charge (−0.72) among all the metals when mixing with the POM (Co-TCPP, Cu-TCPP, Mn-TCPP, Ni-TCPP, and Zn-TCPP have charges of −0.64, −0.64, −0.82, −0.64 and −0.11, respectively). This will result in a higher LUMO level of the POM, which will increase the overpotential and then the activity efficiency for CO_2_RR. This is the main cause of the higher photocatalytic activity of Fe-POMOFs than other M-POMOFs.

As for the ECR reaction, we calculated the FEDs of Fe-TCPP and the POM under the critical potential of −0.7 V (versus RHE; Fig. 5B and figs. S58 and S59). Under this condition, the smaller value of the key process (Δ*G*^CO^_2_) was found, indicating that Fe-TCPP preferred CO pathway than CH_4_ in the catalysis (fig. S58). In addition to the Δ*G*_2_ values, all the elementary steps were exothermic in the POM (fig. S59), but Fe-TCPP contained one endothermic step as shown in fig. S60 (*HCHO→*CH, Δ*G* = 0.24 eV). These results also confirmed that the CO pathway was favored by Fe-TCPP as the active site in ECR. Therefore, the whole process could be concluded, as CO_2_(g) was first reduced to *COOH, then to *CO, and lastly to CO(g) on Fe-POMOF (Fig. 5B). During the process, Fe-TCPP was recognized as the active site for electrocatalysis, which was consistent with the experimental observation for CO generation. The different products between PCR and ECR reactions could be attributed to their deviated active sites: POM for photocatalysis and Fe-TCPP for electrocatalysis. For further explaining the electrocatalytic active centers, the activities of CO generation at the different metal centers were investigated and compared at −0.7 V. As shown in Fig. 5B, the potential determining step (PDS) was CO release (the last step) in Co-POMOF, while the PDS in other M-POMOFs were the generation of *COOH (the first step), with their respective Δ*G*_PDS_ of 0.007 eV for Fe, 0.014 eV for Co, 0.74 eV for Ni, 1.43 eV for Cu, 0.06 eV for Mn, and 1.41 eV for Zn in M-POMOFs. This indicated the outstanding activities shown by Fe-POMOF, much higher than those of other metal-derived frameworks, which was consistent with their FE_CO_ at −0.7 V (Fig. 5B). About the intrinsic cause of the trend, one can see the partial density of states (PDOS) of the metal centers (fig. S60). The PDOSs of Ni, Zn, and Cu are quite symmetrical, while those of Co, Fe, and Mn are asymmetrical, indicating that Co, Fe, and Mn have higher spin density, which will result in a higher reactivity, i.e., strong binding. This trend was found consistently in the FEDs of CO_2_RR. As a result, their respective ΔG_*COOH_ order (Co < Fe < other metals) in M-POMOFs were obtained. As for Co, Mn, and Fe, originally, one may expect Fe and Mn to bind CO stronger than Co, because Co has a lower spin density (indicated by the relatively more symmetrical PDOS). However, as indicated by the position of *π orbital of CO, the overlap is stronger in Co than Fe and Mn. This will generate a higher *π back bonding effect, giving CO additional energy. For O species such as OH, since there is no such *π back bonding effect, CO adsorption on Co is stronger than on Fe. The results showed that CO is difficult to release on Co. Thus, the PDS is CO released in Co-POMOF but not the first step. Therefore, these are the reasons for the trend of Δ*G*_PDS_ results among the six metals in the porphyrin center (Fig. 4B).

According to the results from experiments and DFT calculations, the PCR and ECR catalytic mechanisms in Fe-POMOF were proposed by the POM cluster and single metal site (Fig. 5D). In the PCR reaction, the activated Fe-TCPP (by visible light) created the electron-hole pairs and allowed the procreant electrons to transfer to the cluster. The POM acted as the active site to reduce CO_2_ to CH_4_. The whole catalysis underwent the processes of eight-electron transfer, eight-proton migration, and the removal of two water molecules. In the ECR reaction, the coupling between POM and Fe-TCPP could form the channel for transporting the electrons to the metal active sites under the exertion of the electric field. Then, the CO derived from CO_2_ was adsorbed and activated by Fe-TCPP. The discrepancies among the catalytic performance were mainly attributed to their respective energy barrier demand during the adsorption or desorption of intermediates.

## DISCUSSION

In the photocatalysis, the binding energy of N of Fe-TCPP moved to the direction of high value, and parts of Mo(V) and Mo(VI) were reduced to Mo(IV) under visible light. This phenomenon might be attributed to the fact that electrons were lost from the TCPP ligand and captured by the POM in Fe-POMOF. In other words, it meant that photogenerated electrons flowed from Fe-TCPP to POM during PCR reaction. Fe-TCPP ligand could harvest the light and be activated to produce the photogenerated electrons, and the electrons subsequently flowed from Fe-TCPP to POM for CH_4_ formation. This result has been supported by a combination of in situ XPS, in situ IR, and DFT calculations, which have been discussed above.

In the electrocatalysis, the control experiments and in situ FTIR results evidenced that Fe-TCPP was the active site of the whole catalyst during the catalytic process. In other words, the results meant that the external electrons flowed to the active site (Fe-TCPP) for CO_2_ reduction. The catalyst was an integrated system containing both Fe-TCPP and the POM. After adding electrons to the catalyst, the electrons in the catalyst flowed to Fe-TCPP through the POM. For further supporting this result, the cyclic voltammogram (CV) experiment of Fe-POMOF was conducted, revealing that both the POM and Fe-TCPP have the ability to obtain/lose electrons (fig. S61). At the reduction potential, the occurrences of the characteristic peaks for the POM (I−III) and Fe (IV and V) indicated that both the POM and Fe-TCPP have the ability to obtain electrons. When the reduction potential was applied to provide electrons to Fe-POMOF, the external electrons could be obtained by POM and Fe-TCPP. Because the catalyst was a whole unit, after the catalytic site was determined, the flow direction of electrons in the catalyst was from the inactive component (POM) to the active one (Fe-TCPP). In this study, as we confirmed that Fe-TCPP had been regarded as the active site by in situ FTIR and control experiments in the electrocatalysis and the oxidation states for Mo in the POM did not change after the ECR, we revealed that the POM could capture electrons from the external reduction potential and then transfer them to the center of Fe-TCPP for CO generation.

The novelty and significance of this work were revealed as follows: On one hand, the POMOF catalysts were constructed from metalloporphyrin and POM cluster, representing an efficient combination of monometallic active site and polymetallic active cluster. It is precisely because of this structural composition feature that POMOF catalyst has been proved that it can simultaneously complete efficient PCR and ECR reaction using different catalytic active components in the same structure. We used this model catalyst system to explore the effects of different catalytic active units (single metal active site and multimetal active cluster) on the reduction product species and catalytic performance (activity, selectivity, durability, etc.) during the photo- and electro-driven CO_2_ reduction reactions, respectively. Furthermore, the results showed that the charge transfer directions between different substrates are completely opposite during the reaction under photo/electric external stimuli, directly leading to the changes in the final reduction products and the catalytic properties. This point has never been reported in the previous studies either. The structure-property relationships designed in the study should be of great importance for inspiring the future design and synthesis of high-performance POMOF catalysts for bifunctional catalytic reactions (photocatalytic and electrocatalytic) or photoelectric coupling reactions.

On the other hand, these POMOF crystal catalysts in the study, effectively constructed by photosensitive component (M-TCPP) and strong reductive unit (Zn-ε-Keggin cluster for the multielectron transferring), could achieve the high selectivity for CH_4_ (97.2%), yield (922 μmol g^−1^), and conversion rate (~154 μmol g^−1^ hour^−1^) in PCR reaction. These high numbers were much higher than many reported works (table S1). It is well known that the transferring of CO_2_ into multielectron products is very difficult, such as CH_4_ involving the processes of eight-electron and eight-proton migration. In most cases, CO and HCOOH are the main products, as they are relatively easy to generate by involving only two-electron and two-proton migration. Therefore, a structural assembly strategy that introduces the strong reductive unit into POMOF catalyst is very critical for promoting the generation of multielectronic coupled protonic products in CO_2_ reduction. In addition, we could also clearly identify the roles of active components in the same structure and their synergistic coupling effects in different catalytic reactions.

In summary, we successfully synthesized a series of M-POMOFs by assembling the POM (Zn-ε-Keggin) and six M-TCPPs into the isostructural framework. The catalytic activities and selectivities of M-POMOFs were investigated in PCR and ECR processes, especially concentrating on the respective and interactive roles between the single metal site and the POM cluster. Fe-POMOF in the PCR produced CH_4_ (922 μmol g^−1^) with the selectivity of 97.2% through visible light–driven catalysis, while that in the ECR exhibited a superior Faradaic efficiency of 92.1% in converting CO_2_ to CO at −0.7 V. The PCR and ECR reaction mechanisms were further cross-verified by both experimental results and theoretical calculations. From the results, it can be evident that the photogenerated electrons flowed from the Fe-TCPP to the POM for fulfilling the multiple-electron migration under the visible light, while the abundant electrons could flow to the center of Fe-TCPP under the exertion of electric field. POM clusters acted as the photocatalytic active site for the generation of CH_4_, while Fe-TCPP could be the electrocatalytic active site to produce CO. It is anticipated that this work will provide theoretical guidance for the structural design of photocatalysts and electrocatalysts and help to understand the directional charge transfer induced by different driving forces and contribute to clarifying the relationships between single metal site and cluster catalysis.

## METHODS

### Synthesis of M-POMOFs

Na_2_MoO_4_·2H_2_O (310 mg, 1.28 mmol), H_3_PO_3_ (10 mg, 0.125 mmol), zinc chloride (68 mg, 0.50 mmol), and tetrabutylammonium hydroxide 10 weight % of solution in water (250 μl) and H_2_O (3.5 ml) were charged in a Pyrex vial and stirred for 10 min. Then, the pH value of the mixture was adjusted to 5.0 by 2 M HCl. Subsequently, Fe-TCPP (33 mg), Mo powder (99.99%; 25 mg, 0.26 mmol), and appropriate amount of dimethylacetamide were added into the mixture and stirred for 15 min. The mixture was sealed in a 15-ml Teflon-lined stainless steel container and then heated at 180°C for 72 hours. After cooling to room temperature at 15°C hour^−1^, dark violet block crystals of Fe-POMOF were collected. The preparation processes of Co-POMOF, Ni-POMOF, Cu-POMOF, Mn-POMOF, and Zn-POMOF were similar to Fe-POMOF except that Fe-TCPP (33 mg) was replaced by Co-TCPP (132 mg), Ni-TCPP (132 mg), Cu-TCPP (99 mg), Mn-TCPP (33 mg), and H_2_-TCPP (118 mg) [Cambridge Crystallographic Data Centre (CCDC): 1832957, 1832956, 1832958, and 1811860, respectively].The good crystal data of Cu-POMOF and Mn-POMOF have not been obtained, but PXRD and IR show that they are isomorphic with these crystal structures. The N_2_ and CO_2_ isotherms are shown in figs. S62 and S63.

### Photochemical measurements

The PCR experiments were probed on evaluation system (CEL-SPH2N, CEAuLight, China) in a 100-ml quartz container, and the two round openings of the container were sealed with rubber mats. A xenon arc lamp (CEL-HXF300/CEL-HXUV300) with a UV cutoff filter (420 to 800 nm) was used as irradiation source. The light intensity is about 110 mW cm^−2^. The as-prepared photocatalyst (5 mg) was evacuated in mixed solutions (30 ml) with AR TEOA (2 ml) and deionized water (28 ml) and predegassed with CO_2_ (99.999%) for 30 min to remove air before irradiation. The pH values were determined to be 10.5, 10.5, and 7.3 in the absence of the catalyst with TEOA, after suspension of the catalyst, and after sparging with CO_2_ (30 min). The sealed reaction system (CO_2_ pressure of 1 atm) was positioned about 20 cm away from visible light source and kept stirring constantly to ensure the photocatalyst particles in suspension. The reactor was connected with a circulating cooling water system to maintain the solution’s temperature at around 20°C. Liquid product was measured by ion chromatography system (Dionex Aquion RFIC, Thermo Fisher Scientific). Gaseous product was measured by GC (column type: TDX-1; GC-7900, CEAuLight, China) equipped with a flame ionization detector (FID) and a thermal conductivity detector (TCD). The isotope-labeled experiments were recorded by GC–mass spectrometry (GC-MS; 7890A and 5875C, Agilent).

### Photoelectrochemical studies

The electrochemical experiments were performed on the electrochemical workstation (CHI660e) or the electrochemical workstation (VSP Potentiostat, BioLogic) in a standard three-electrode system. The working electrodes for photocurrent and Mott-Schottky tests were prepared as follow: The as-synthesized crystals (2 mg) were grinded to powder and then dispersed in 1 ml of solvent (990 μl of ethanol and 10 μl of 0.5% Nafion) by ultrasonication to form the ink. Subsequently, 200 μl of the ink was covered onto both sides of indium tin oxide (ITO) glass and dried in room temperature for photocurrent and Mott-Schottky tests. Photocurrent tests were carried out in 0.2 M Na_2_SO_4_ aqueous solution at a bias of 0.0 V, using a xenon lamp (λ ≥ 420 nm) as the light source, Ag/AgCl electrode as the reference electrode, and Pt plate as the counter electrode. The Mott-Schottky tests were performed in 0.2 M Na_2_SO_4_ solution, using above catalyst/ITO electrode as the working electrode; Ag/AgCl electrode as the reference electrode; and Pt plate as the counter electrode at different frequencies of 500, 1000, and 1500 Hz. The TSPL decay curves were obtained on an FLS1000 fluorescence at room temperature (25°C). Edinburgh FLS980 Spectral excitation light source: A xenon lamp of 450 W, a visible region of 200 to 780 nm, and an IR region of 780 to 1600 nm. Life light source: Microsecond lamp and nanosecond lamp (pulse light source).

### Electrochemical measurements

All electrocatalysis tests of these catalysts were carried out on the electrochemical workstation (BioLogic) using the standard three-electrode configuration (Ag/AgCl electrode and carbon electrode acted as the reference and counter electrode, respectively) in an airtight H-type cell injected with 0.5 M KHCO_3_ (CO_2_-saturated solution, pH 7.2). To enhance the conductivity of the MOF catalysts, the acetylene black (AB) was introduced in the pure MOF crystals by grinding the mixture (*54*). In many reported works, Nafion solution as a dispersion solution was generally used to form a uniform ink with MOF and AB, which was propitious to attach to the surface of carbon paper. The preparation process of the working electrode is as follows: 10 mg of AB and 10 mg of pure MOF crystal as catalyst were ground uniformly, and the 0.5% Nafion solution (1000 μl) was added. To mix evenly, the mixture needs sonication over 30 min. Then, the mixture (100 μl × 2) was dropped on two sides of a carbon paper (1 cm by 1 cm). After the natural drying, the working electrode (total area, 1 cm by 2 cm) can be used for test.

At a scan rate of 5 mV s^−1^, LSV mode was used to obtain the polarization curves in ECR experiments (*54*). The polarization curves of the working electrode were recorded under the inert atmosphere (Ar gas) and the CO_2_ (99.999%), respectively. The FE experiments were performed from −0.5 to 1.2 V (versus RHE) in the 0.5 M KHCO_3_ solution bubbling with CO_2_ (99.999%) atmosphere about 40 min, and the calculation about FE of catalysts was shown in the Supplementary Materials. On the basis of the Nernst equation: *E* (versus RHE) = *E* (versus Ag/AgCl) + 0.1989 V + 0.059 × pH, the test results were reported versus RHE. To evaluate the electrochemcial active surface area (ECSA), the *C*_dl_ were recorded by CVs with various scan rates from 10 to 50 mV s^−1^. Electrochemical impedance spectroscopy experiments were executed, ranging from 100 kHz to 0.1 Hz at an overpotential (−0.7 V versus RHE) using an AC voltage with 10-mV amplitude on the electrochemical workstation (VSP, BioLogic).

The generated gaseous products were analyzed by a GC (7900, CEAuLight, China) equipped with an FID and a TCD with the helium as carrier gas (*54*). The results of isotope-labeled experiments (^13^CO_2_ instead of CO_2_) were analyzed by GC-MS (7890A and 5875C, Agilent). After reaction, the liquid products were collected and quantified by NMR (Bruker Avance III 400) spectroscopy. The specific operation is as follows: 0.4 ml of electrolyte after reaction (−0.7 V) and 0.1 ml of D_2_O. Solvent presaturation technique was implemented to suppress the water peak.

### In situ measurements

The in situ IR measurements of photoreduction were carried out using an FTIR spectrophotometer (Nicolet iS50 FTIR, Thermo Fisher Scientific) within a photoreactor using a mercury cadmium telluride (MCT) detector. The photocatalyst was evenly inside the photoreactor for monitoring reaction progress of PCR. Next, heating and purging under N_2_ atmosphere were done to remove the air and any adsorbed species, and then 99.999% CO_2_ gas along with water vapor was passed for 30 min inside the photoreactor. Last, visible light was irradiated on catalyst using xenon lamp light (>420 nm). In situ FTIR signal was collected through MCT detector with a resolution of 4 cm^−1^ and eight scans at a regular time interval. The in situ IR measurements of electroreduction were carried out using an FTIR spectrophotometer (Nicolet iS50 FTIR, Thermo Fisher Scientific) within an MCT detector. Experiments in 0.5 M KHCO_3_ were conducted in a newly designed spectroelectrochemical cell in which the Si ATR (attenuated total reflectance) crystal is mounted on the side of the cell. A three-electrode system is similar to electrocatalysis. After the gold sputtering treated on the reflecting plane of a Si prism, the catalyst ink that was dropped evenly on the electrode was used as the working electrode. KHCO_3_ electrolytes were prepared by purging with high-purity CO_2_ gas (99.999%) over 30 min. All spectroscopic measurements were collected with a resolution of 4 cm^−1^ and eight scans. A PIKE Technologies VeeMAX III ATR accessory was used for experiments in 0.5 M KHCO_3_. Spectra are presented in absorbance where positive and negative peaks signify an increase and decrease in the corresponding interfacial species, respectively. The in situ XPS measurements of photoreduction were carried out by Thermo Fisher Scientific (ESCALAB 250Xi) equipped with light source device and atmosphere input device. The spectrums were recorded before visible light, visible light for 30 min, and after photoreduction of CO_2_.

### Theoretical calculations

Spin-polarized DFT calculations were performed with periodic supercells under the generalized gradient approximation (GGA) using the Perdew-Burke-Ernzerhof (PBE) functional for exchange correlation and the ultrasoft pseudo-potentials for nuclei and core electrons. The Kohn-Sham orbitals were expanded in a plane-wave basis set with a kinetic energy cutoff of 30 rydberg (Ry) and the charge-density cutoff of 300 Ry. These values were verified to be converged when calculating the reaction energy in both CO and CH_4_ pathways. The Fermi surface effects have been treated by the smearing technique of Methfessel and Paxton, using a smearing parameter of 0.02 Ry. The convergence criteria are set as 10^−3^ Ry bohr^−1^ of Cartesian force components acting on each atom and 10^−6^ Ry of total energy. Only gamma points were sampled in the Brillouin zones. The plane-wave self-consistence field (PWSCF) codes contained in the Quantum ESPRESSO distribution were used to implement the calculations. The correction was from the zero-point energy, entropy, and heat capacity for converting the total energies to Gibbs free energies (in electron volts).

### The details for composing the FED

During calculation, the HER is considered to go through the following mechanism

H^+^ + *e* + *→H* (RS1) *H + H^+^ + *e*→H_2_ + * (RS2)

In addition, that for CO_2_RR toward CO is

CO_2_ + H^+^ + *e* + *→*COOH (RS3)

*COOH + H^+^ + *e*→*CO + H_2_O (RS4)

*CO→CO(g) + * (RS5)

We also have the mechanism for CO_2_RR toward CH_4_

CO_2_ + H^+^ + *e* + *→*COOH (RS3)

*COOH + H^+^ + *e*→*HCOOH (RS6)

H*COOH + H^+^ + *e*→H*C(OH)_2_ (RS7)

H*C(OH)_2_→*CHO + H_2_O (RS8)

*CHO + H^+^ + *e*→*HCHO (RS9)

*HCOH + H^+^ + *e*→*CH + H_2_O (RS10)

*CH + H^+^ + *e*→*CH_2_ (RS11)

*CH_2_ + H^+^ + *e*→*CH_3_ (RS12)

*CH_3_ + H^+^ + *e*→*^+^CH_4_(g) (RS13)

In calculating the FEDs from RS1 to RS13, the free energies of proton and electron are treated via the classical computational hydrogen electrode method. As for the adsorbates, the associated adsorption free energies of the adsorbates are calculated by the following expression

$$G_{A}=E_{A}+\mathrm{ZPE}-\mathrm{TS}+\int C_{\;p}dT$$ (1)

where *E*_A_ is the total energy of a certain molecule *A* or adsorbate A*. For molecule, *E*_A_ can be obtained directly through a gas phase calculation. For a certain adsorbate, *E*_A_ is calculated by the difference between the DFT-based substrate with (*E*_A*_^DFT^) and without adsorbate A (E_*_^DFT^)

$$E_{A}={E_{A}*}^{\mathrm{DFT}}-{E*}^{\mathrm{DFT}}$$ (2)

ZPE, TS, and ∫*C*_p_d*T* are the correction from zero-point energy, entropy, and heat capacity, whose values are listed on table S7. Other than that, H^+^ is calculated by the Gibbs free energy of ½H_2_, and the energy of electron is calculated by −*Ue*. As for the solvation energies, a value of −0.11 eV is added to each O atom of adsorbates. For instance, −0.11 and −0.22 eV are added for *CO and *COOH. A correction of −0.51 eV is added to CO molecules for the errors for GGA-PBE functional. According to this correction, it can lead an agreement with experimental overall half reaction of CO_2_ reduction. Specifically, the standard reaction Gibbs free energy differences from CO_2_ or H_2_ to the associated intermediates *A* (denoted as Δ*G*^0^_*A_) are expressed respectively as

$$\Delta G^{0}{*}_{\mathrm{COOH}}=G{*}_{\mathrm{COOH}}-G_{\mathrm{CO}2}-1/2G_{H2}$$ (3)

$$\Delta G^{0}{*}_{\mathrm{CO}}=G{*}_{\mathrm{CO}}-G_{\mathrm{CO}2}-G_{H2}+G_{H2O}$$ (4)

$$\Delta {G^{0}}_{H}*=G_{H}*-1/2G_{H2}$$ (5)

$$\Delta G^{0}{*}_{\mathrm{HCOOH}}=G{*}_{\mathrm{HCOOH}}-G_{\mathrm{CO}2}-G_{H2}$$ (6)

$$\Delta {G^{0}*}_{\mathrm{HC}(\mathrm{OH})2}={G*}_{\mathrm{HC}(\mathrm{OH})2}-G_{\mathrm{CO}2}-3/2G_{H2}$$ (7)

$$\Delta G^{0}{*}_{\mathrm{CHO}}=G{*}_{\mathrm{CHO}}-G_{\mathrm{CO}2}-3G_{H2}+G_{H2O}$$ (8)

$$\Delta G^{0}{*}_{\mathrm{HCHO}}=G{*}_{\mathrm{HCHO}}-G_{\mathrm{CO}2}-2G_{H2}+G_{H2O}$$ (9)

$$\Delta G^{0}{*}_{\mathrm{CH}}=G{*}_{\mathrm{CH}}-G_{\mathrm{CO}2}-5/2G_{H2}+2G_{H2O}$$ (10)

$$\Delta {G^{0}*}_{\mathrm{CH}2}={G*}_{\mathrm{CH}2}-G_{\mathrm{CO}2}-3G_{H2}+2G_{H2O}$$ (11)

$$\Delta {G^{0}*}_{\mathrm{CH}3}={G*}_{\mathrm{CH}3}-G_{\mathrm{CO}2}-7/2G_{H2}+2G_{H2O}$$ (12)

The standard Gibb free energy differences from RS1 to RS13are then expressed as

$$\Delta {G^{0}}_{\mathrm{RS}1}=G*H-1/2G_{H2}+Ue$$ (13)

$$\Delta {G^{0}}_{\mathrm{RS}1}=G*H-1/2G_{H2}+Ue$$ (14)

$$\Delta {G^{0}}_{\mathrm{RS}3}=\Delta G^{0}*\mathrm{COOH}+Ue$$ (15)

$$\Delta {G^{0}}_{\mathrm{RS}4}=\Delta G^{0}*\mathrm{CO}-\Delta G^{0}*\mathrm{CO}\mathrm{OH}+Ue$$ (16)

$$\Delta {G^{0}}_{\mathrm{RS}5}=G_{\mathrm{CO}(g)}-\Delta G^{0}*\mathrm{CO}+Ue$$ (17)

$$\Delta {G^{0}}_{\mathrm{RS}6}=\Delta G^{0}*\mathrm{HCOOH}-\Delta G^{0}*\mathrm{COOH}+Ue$$ (18)

$$\Delta {G^{0}}_{\mathrm{RS}7}=\Delta G^{0}*\mathrm{HC}(\mathrm{OH})2-\Delta G^{0}*\mathrm{HC}\mathrm{OOH}+Ue$$ (19)

$$\Delta {G^{0}}_{\mathrm{RS}8}=\Delta G^{0}*\mathrm{CHO}-\Delta G^{0}*\mathrm{HC}(\mathrm{OH})2$$ (20)

$$\Delta {G^{0}}_{\mathrm{RS}9}=\Delta G^{0}*\mathrm{HCHO}-\Delta G^{0}*\mathrm{CHO}+Ue$$ (21)

$$\Delta {G^{0}}_{\mathrm{RS}10}=\Delta G^{0}*\mathrm{CH}-\Delta G^{0}*\mathrm{HCHO}+Ue$$ (22)

$$\Delta {G^{0}}_{\mathrm{RS}11}=\Delta G^{0}*\mathrm{CH}2-\Delta G^{0}*\mathrm{CH}+Ue$$ (23)

$$\Delta {G^{0}}_{\mathrm{RS}12}=\Delta G^{0}*\mathrm{CH}3-\Delta G^{0}*\mathrm{CH}2+Ue$$ (24)

$$\Delta {G^{0}}_{\mathrm{RS}13}=G_{\mathrm{CH}4}-4G_{H2}+2G_{H2O}-\Delta G^{0}*\mathrm{CH}3+Ue$$ (25)

We used Eqs. 13 to 25 to calculate the FEDs.

## Supplementary Materials

### This PDF file includes:

Supplementary Text Figs. S1 to S63 Tables S1 to S7 References
