# Supplementary material for: Demystifying the roles of single metal site and cluster in CO2 reduction via light and electric dual-responsive polyoxometalate-based metal-organic frameworks
Source: Sci Adv. 2022 Dec 9;8(49):eadd5598. doi: 10.1126/sciadv.add5598 (PMC9733922; doi:10.1126/sciadv.add5598)
Supplement: Supplementary file 1 — Supplementary Text Figs. S1 to S63 Tables S1 to S7 References [file sciadv.add5598_sm.pdf]

Science Advances
AAAS

Supplementary Materials for

Demystifying the roles of single metal site and cluster in CO_2 reduction via light and electric dual-responsive polyoxometalate-based metal-organic frameworks

Qing Huang *et al.*

Corresponding author: Ya-Qian Lan, yqlan@m.scnu.edu.cn, yqlan@njnu.edu.cn

Sci. Adv. **8**, eadd5598 (2022)
DOI: 10.1126/sciadv.add5598

This PDF file includes:

Supplementary Text
Figs. S1 to S63
Tables S1 to S7
References

Supplementary Text

Materials and measurements

All reagents and solvents were commercially available and used as received. FTIR spectra in the range of 4000–400 cm^{-1} were obtained *via* KBr pellets on a Bruker Tensor 27 FT/IR spectrophotometer. PXRD patterns of the samples were performed in the range 3-50° on a D/max 2500VL/PC diffractometer with Cu Kα radiation (λ =1.5406 Å) at 298K. The optical absorption and diffused reflectance spectra were carried out on a Cary 5000 UV-Vis-NIR spectrophotometer (Viarian, USA) using $BaSO_4$ as a reflectance standard.

Synthesis of Zn-ε-Keggin (NENU-499) (*36*)

A mixture of sodium molybdate dihydrate (618 mg, 2.55 mmol), Mo powder 99.99% (50 mg, 0.52 mmol), H_3PO_3 (20 mg, 0.25 mmol), zinc chloride (136 mg, 1.00 mmol), tetrabutylammonium hydroxide 40 wt % solution in water (120 μL, 0.18 mmol), and H_2O (8 mL) was stirred for 20 min, and the pH was acidified to 4.8 with diluted HCl (2 M). Then, 6- nitrobenzimidazole (82 g, 0.50 mmol) was added to the mixture, which was transferred and sealed in a 15 mL Teflon-lined stainless steel container and heated at 180 °C for 72 h. After cooling to room temperature at 10 °C·h^{-1}, the dark-red crystals were harvested.

Synthesis of M-TCPP (*45*)

5, 10, 15, 20-Tetrakis(4-methoxycarbonylphenyl)porphyrin (TMCP) (1.7 g, 2.0 mmol) , $FeCl_3·6H_2O$ (6.9 g, 25.6 mmol) and DMF (200 mL) were mixed and refluxed for 12 h. A large amount of water was added after cooling down to room temperature. The as-synthesized material was filtered and washed with water (600 ml) for six times. The fuchsia solid sample was obtained (yield about 90% based on TMCP). Then, the obtained ester, MeOH (50 mL) and THF (50 mL) were mixed and stirred. 50 mL KOH solution (5.2 g, 93.9 mmol KOH was dissolved in 50 mL H_2O) was added into the mixture and was refluxed for one night. After reaction, the organic solvents were evaporated. To make the solid fully dissolve, moderate water was added to the mixed solution and heated. 2 M HCl was used to acidify the obtained fuchsia homogeneous solution until no further fuchsia precipitate was generated. The solid was collected and washed with deionized water until the pH of filtrate reaches about 5. Finally, the solid sample was dried in vacuum at 60 °C. The preparation processes of Co-TCPP, Ni-TCPP, Cu-TCPP, Mn-TCPP and Zn-TCPP were similar to Fe-TCPP except that $FeCl_3·6H_2O$ (6.9 g, 25.6 mmol) was replaced by $CoCl_2·6H_2O$ (6.1 g, 25.6 mmol), $NiCl_2·6H_2O$ (6.2 g, 25.6 mmol), $CuCl_2·2H_2O$ (4.4 g, 25.6 mmol), $MnCl_2·4H_2O$ (5.0 g, 25.6 mmol) and $ZnCl_2$ (3.5 g, 25.6 mmol), respectively.

Syntheses of various M-POMOFs

$Na_2MoO_4·2H_2O$ (310 mg, 1.28 mmol), H_3PO_3 (10 mg, 0.125 mmol), zinc chloride (68 mg, 0.50 mmol), tetrabutylammonium hydroxide (TBAOH) 10 wt % solution in water (250 μL) and H_2O (3.5 mL) were charged in a Pyrex vial and stirred for 10 min. Then, the pH value of the mixture was adjusted to 5.0 by 2 mol/L HCl. Subsequently, 5,10,15,20-tetrakis(4-carboxyphenyl)porphyrinato-Fe (33 mg), Mo powder 99.99% (25 mg, 0.26 mmol) and appropriate amount of dimethylacetamide were added into the mixture and stirred for 15 min. The mixture was sealed in a 15 mL Teflon-lined stainless steel container and then heated at 180 °C for 72 h. After cooling to room temperature at 15 °C·h^{-1}, dark-violet block crystals of

Fe-POMOF were collected. The preparation processes of Co-POMOF, Ni- POMOF, Cu-POMOF, Mn-POMOF and Zn-POMOF were similar to Fe-POMOF except that Fe-TCPP (33 mg) was replaced by Co-TCPP (132 mg), Ni-TCPP (132 mg), Cu-TCPP (99 mg), Mn-TCPP (33 mg) and H_2-TCPP (118 mg).

Reaction product analysis

The calculation of Faradaic efficiency

For CO,

$$\text{FE}=\frac{2F\times n_{co}}{I\times t}\times 100\%$$

For H_2,

$$\text{FE}=\frac{2F\times n_{H_2}}{I\times t}\times 100\%$$

where F is the Faraday constant, n_{CO} is the moles of produced CO and n_{H_2} is the moles of produced H_2.

Turnover Frequency (TOF, h^{-1})

The TOF for CO was calculated as follow:

$$\text{TOF}=\frac{I_{product}/NF}{m_{cat}\times\omega/M_{Fe}}\times 3600$$

$I_{product}$: partial current for certain product, CO;

N: the number of electron transferred for product formation, which is 2 for CO;

F: Faradaic constant, 96485 C mol^{-1};

m_{cat}: catalyst mass in the electrode, g;

ω: Fe loading in the catalyst;

M_{Fe}: atomic mass of Fe, 55. 845 g mol^{-1}.

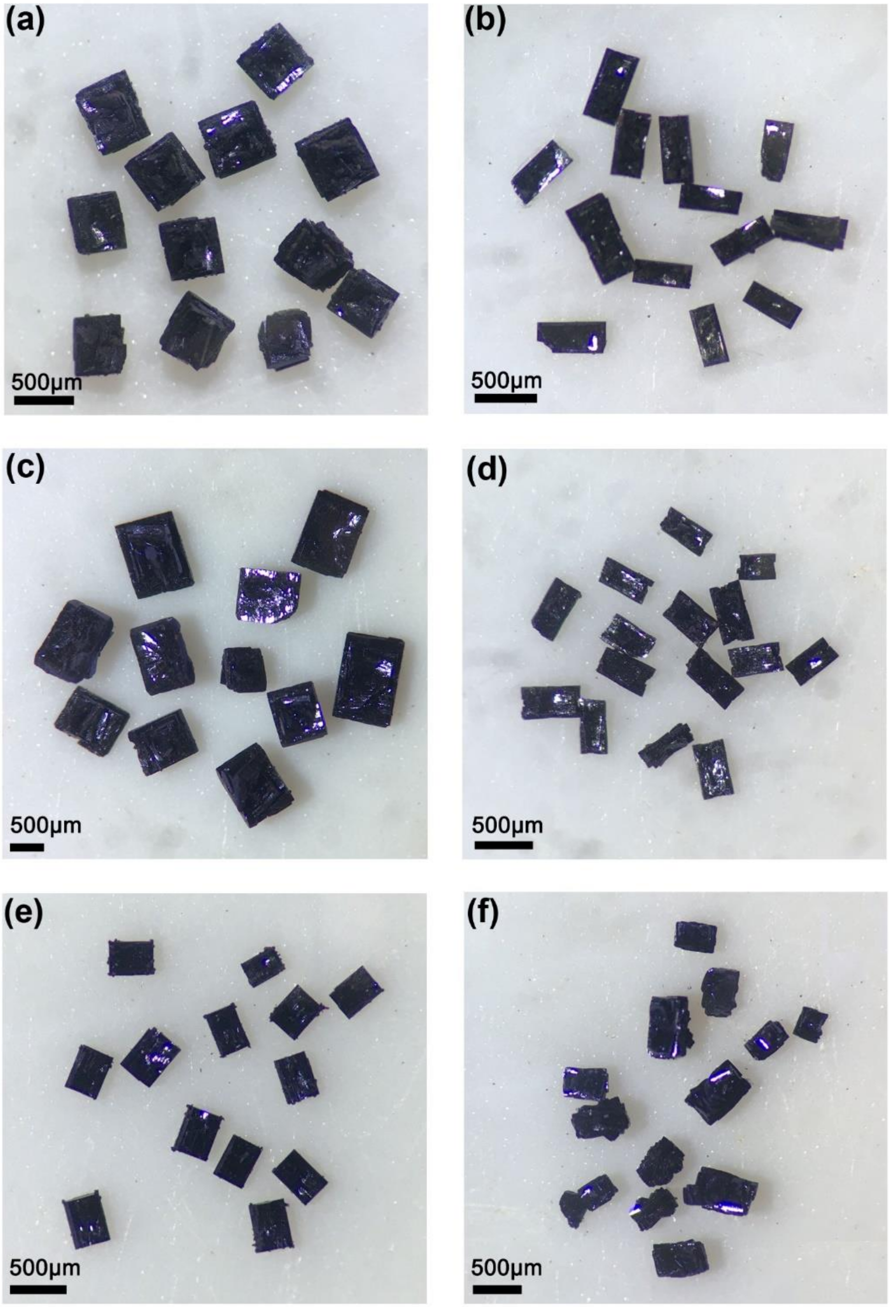

Fig. S1 The images of M-POMOFs under optical microscope (scale bar, 500 μm). (a-f) are Fe, Co, Ni, Cu, Mn and Zn-POMOF.

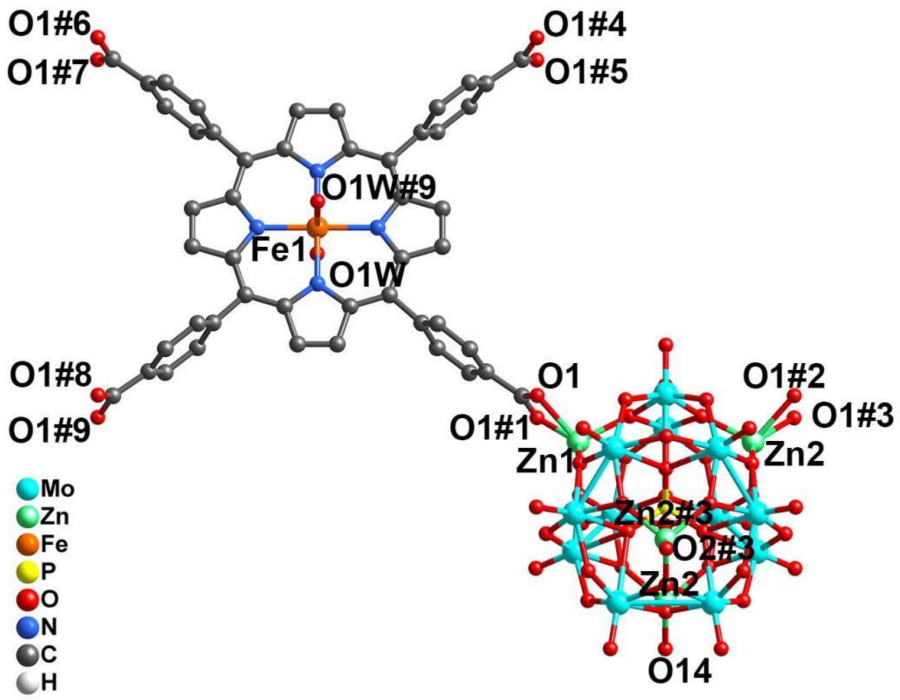

Fig. S2 Summary of the structural information of Zn(II) and Fe (III) centers in Fe-POMOF. Symmetry codes: #1 1 – *x*, *y*, *z*; #2 *x*, 2 – *y*, *z*; #3 1 – *x*, 2 –*y*, *z*; #4 *x*, *y*, 1 – *z*; #5 1 – *x*, *y*, 1 –*z*; #6 *x*, 1 – *y*, 1 – *z*; #7 1 – *x*, 1 – *y*, 1 – *z*; #8 *x*, 1– *y*, *z*; #9 1 – *x*, 1 – *y*, *z*.

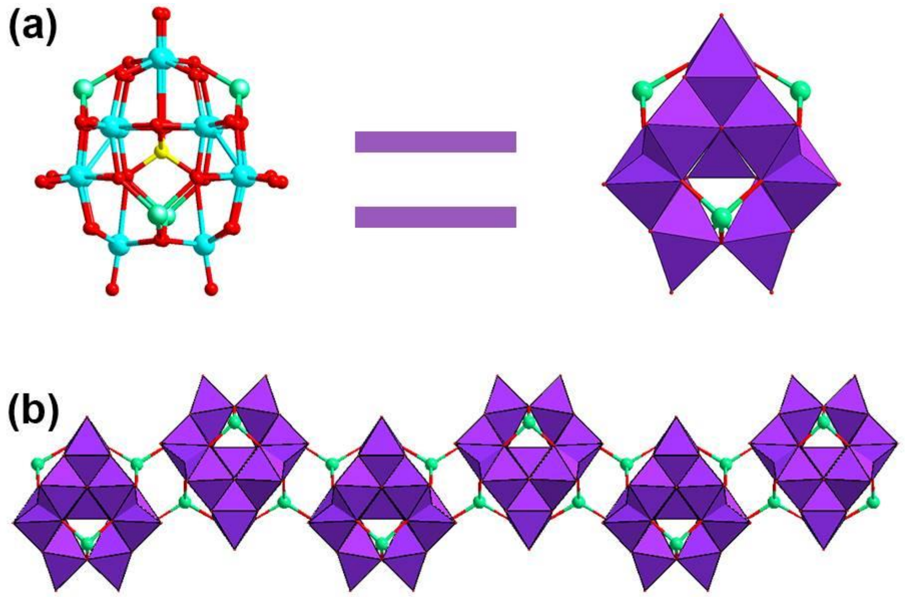

Fig. S3 Ball-and-stick view and polyhedral structure. a. Zn-ε-Keggin culster; **b.** Z-shaped $(\varepsilon Zn)_\infty$ chains

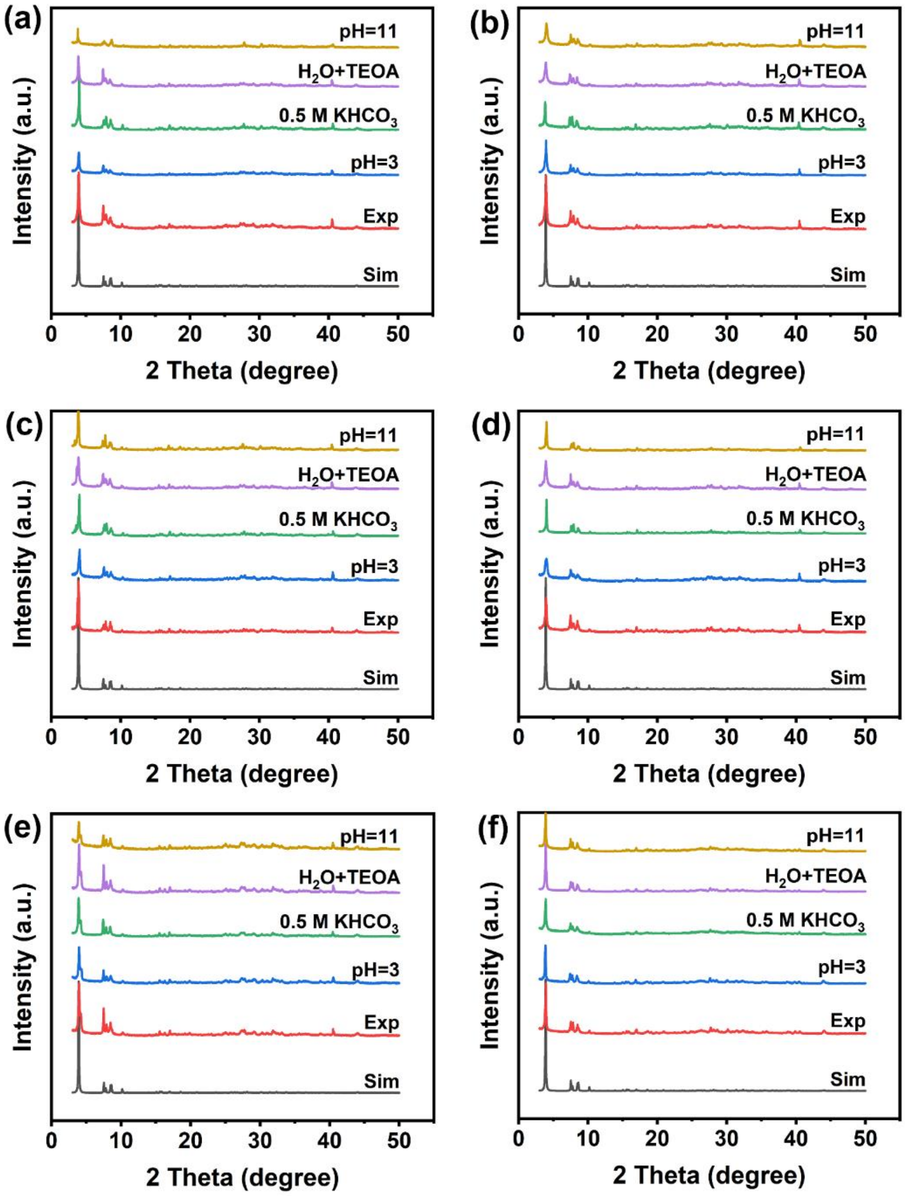

Fig. S4 PXRD patterns. a-f. PXRD patterns of Fe, Co, Ni, Cu, Mn and Zn-POMOF under different conditions for 24 h, indicating their structural robustness upon harsh treatments. "Sim" represents the simulated pattern, and "Exp" represents the pattern of as-synthesized sample.

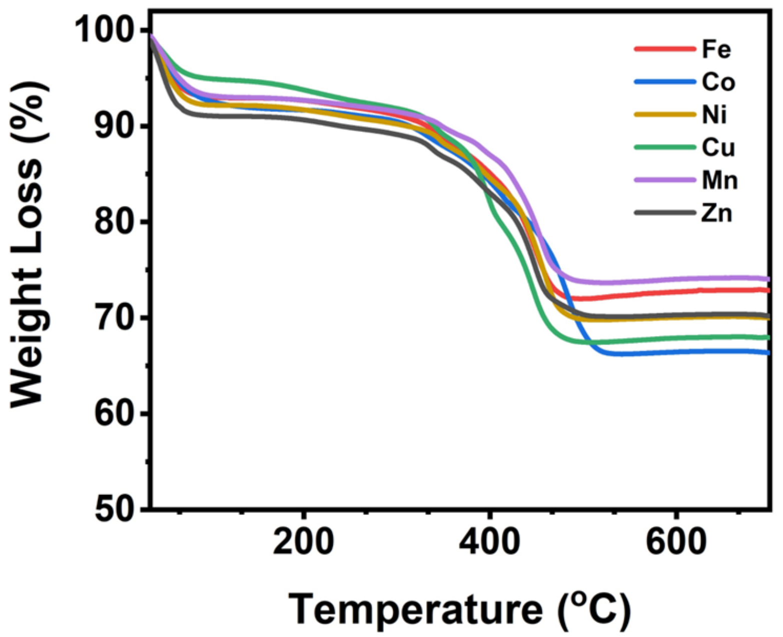

Fig. S5 Thermogravimetric analyses (TGA). TGA results of Fe, Co, Ni, Cu, Mn and Zn-POMOF.

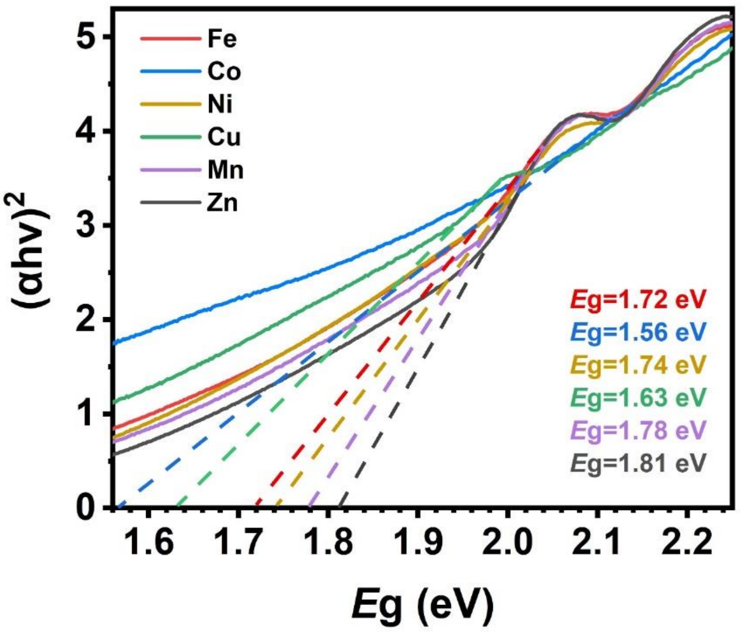

Fig. S6 The band gaps. The band gaps were calculated for M-POMOF samples.

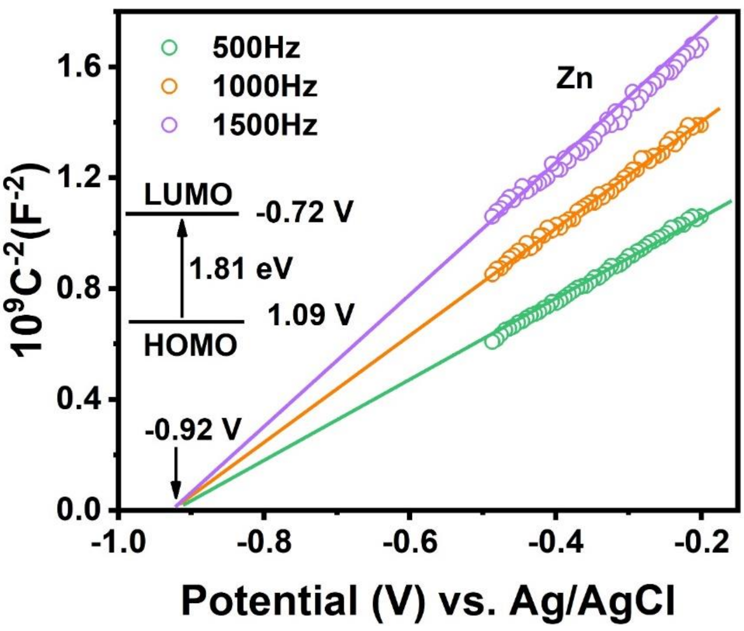

Fig. S7 Mott–Schottky plots. Mott–Schottky plots for Zn-POMOF in 0.2 M Na_2SO_4 aqueous solution.

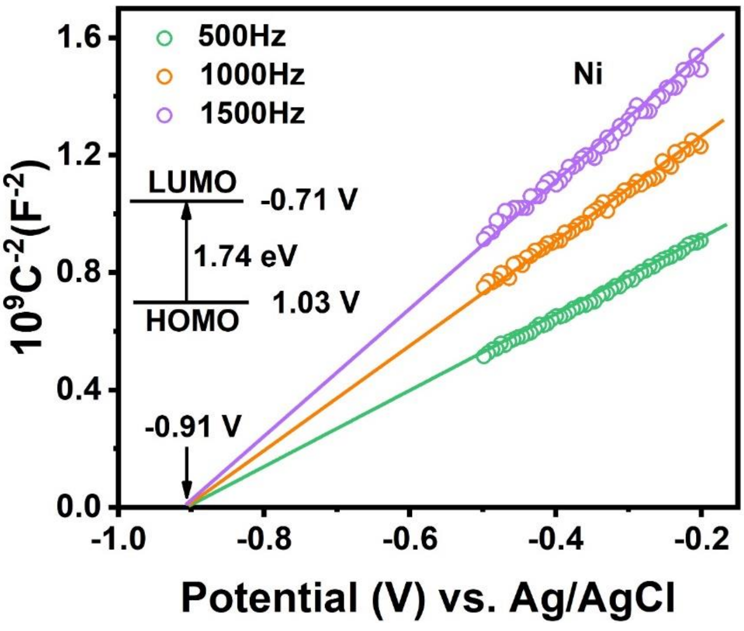

Fig. S8 Mott–Schottky plots. Mott–Schottky plots for Ni-POMOF in 0.2 M Na_2SO_4 aqueous solution.

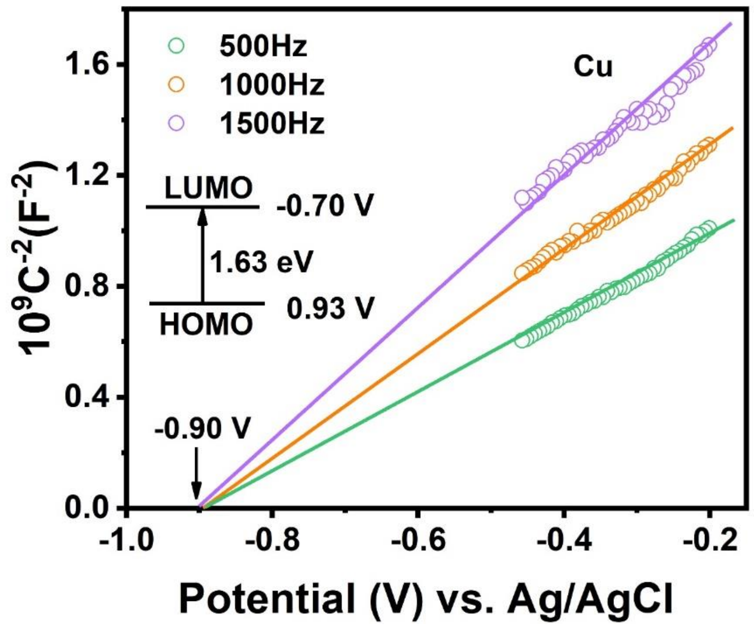

Fig. S9 Mott–Schottky plots. Mott–Schottky plots for Cu-POMOF in 0.2 M Na_2SO_4 aqueous solution.

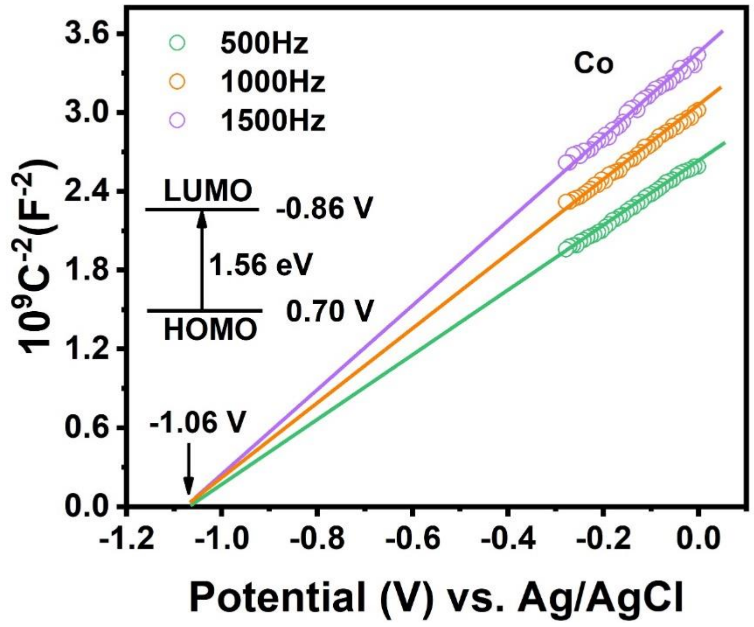

Fig. S10 Mott–Schottky plots. Mott–Schottky plots for Co-POMOF in 0.2 M Na_2SO_4 aqueous solution.

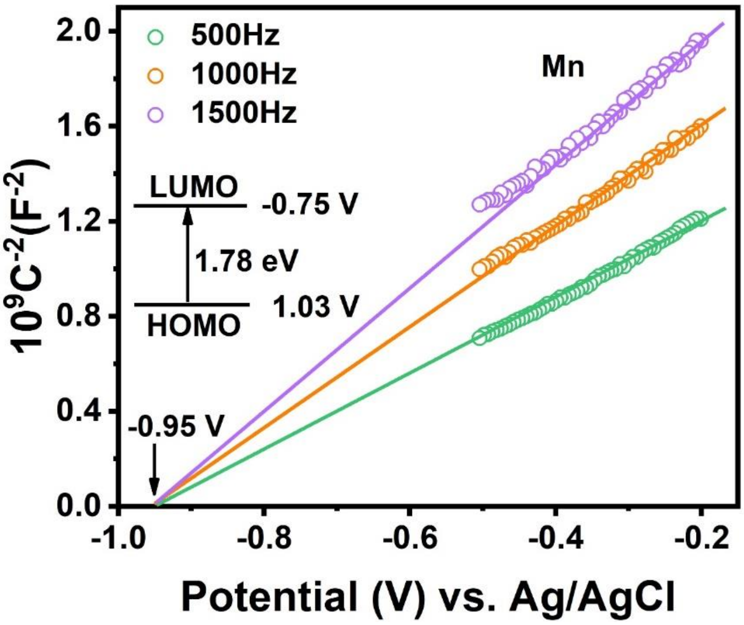

Fig. S11 Mott–Schottky plots. Mott–Schottky plots for Mn-POMOF in 0.2 M Na_2SO_4 aqueous solution.

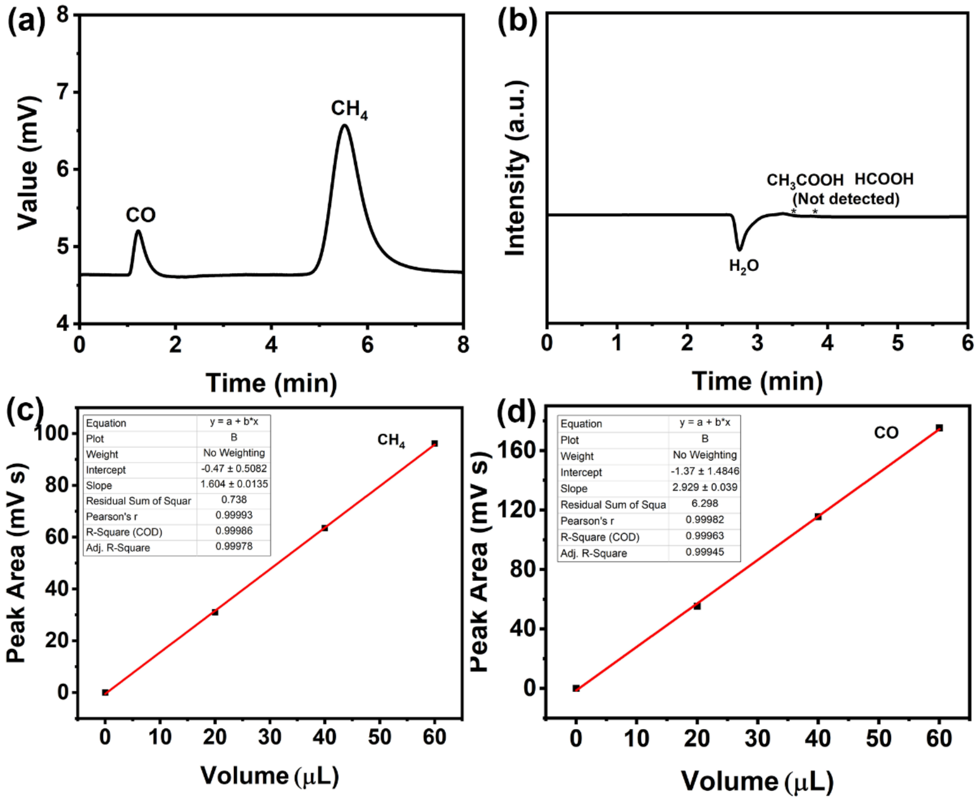

Fig. S12 GC and IC analyses. a. The gas products including CH_4 and CO were detected by gas chromatogram. **b.** The liquid products were not detected. **c.** GC calibration line of CH_4 (y=1.604x-0.47). **d.** GC calibration line of CO (y=2.929x-1.37).

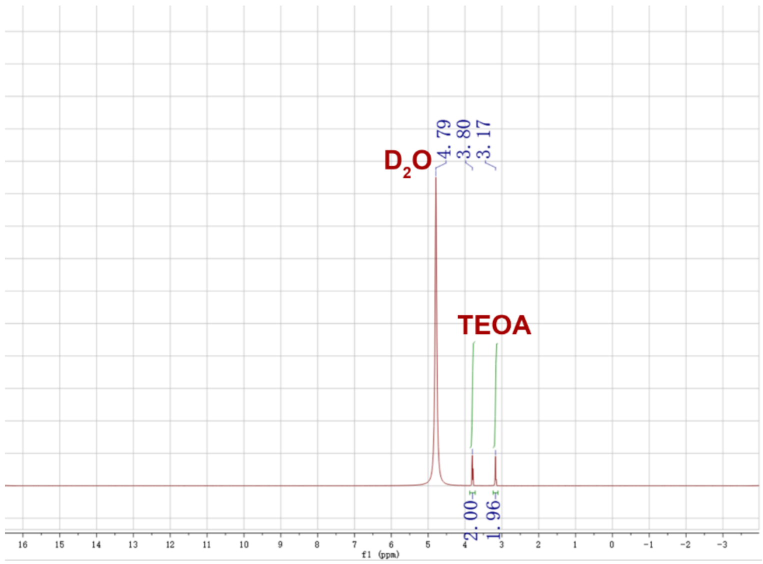

Fig. S13 The ^{1}H NMR spectra of the liquid phases after the PCR experiment for Fe-POMOF. The TEOA was the sacrificial agent in PCR reaction.

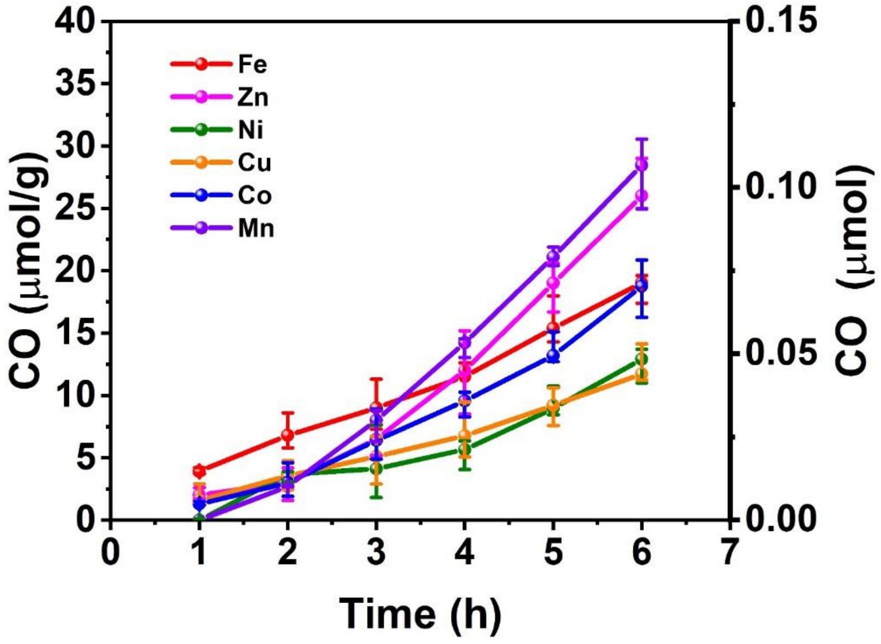

Fig. S14 CO products. Amounts of CO produced as a function of the visible illumination time over Fe, Co, Ni, Cu, Mn and Zn-POMOF.

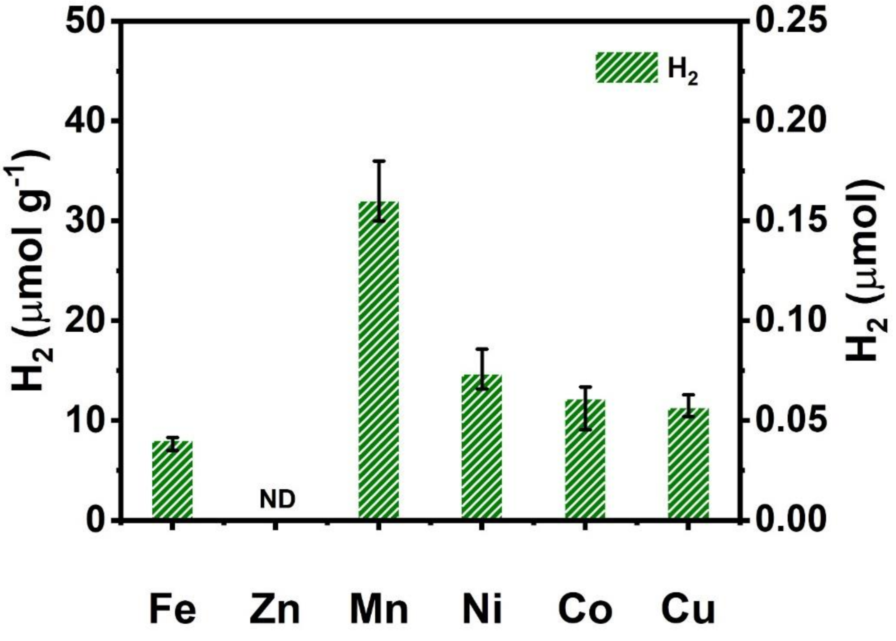

Fig. S15 H_2 pruducts. Amounts of H_2 produced over Fe, Co, Ni, Cu, Mn and Zn-POMOF after 6 h. ND：Not detected.

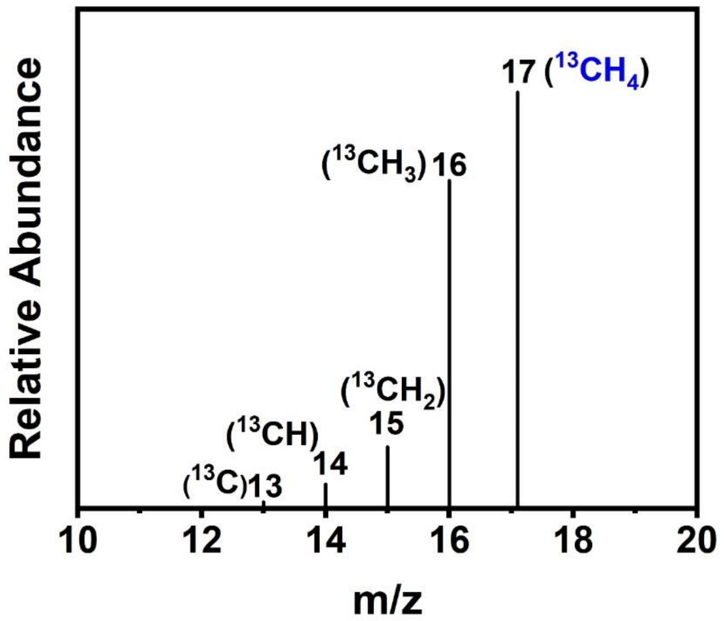

Fig. S16 Gas chromatography and mass spectra. Gas chromatography and mass spectra of $^{13}CH_4$ generation using Fe-POMOF as photocatalyst in photoreduction reaction under $^{13}CO_2$ atmosphere (The single ion monitoring (SIM) mode of mass spectrometry).

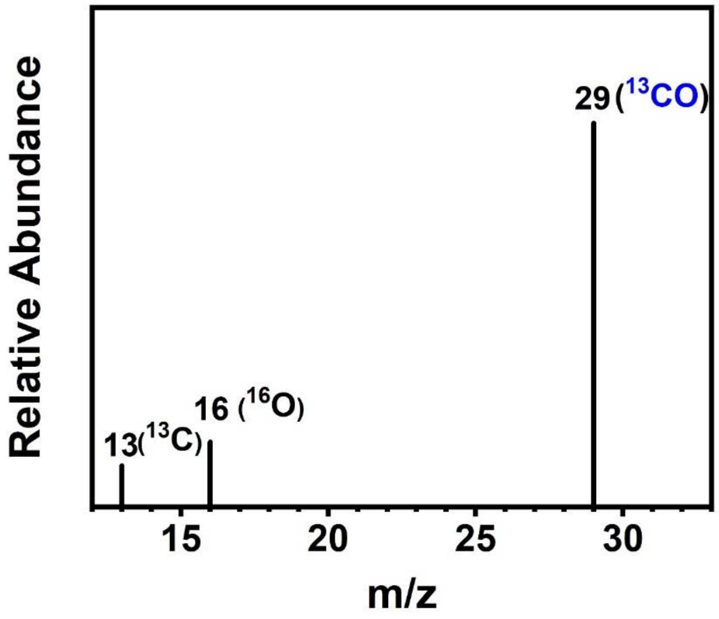

Fig. S17 Gas chromatography and mass spectra. Gas chromatography and mass spectra of ^{13}CO generation using Fe-POMOF as photocatalyst in photoreduction reaction under $^{13}CO_2$ atmosphere (The single ion monitoring (SIM) mode of mass spectrometry).

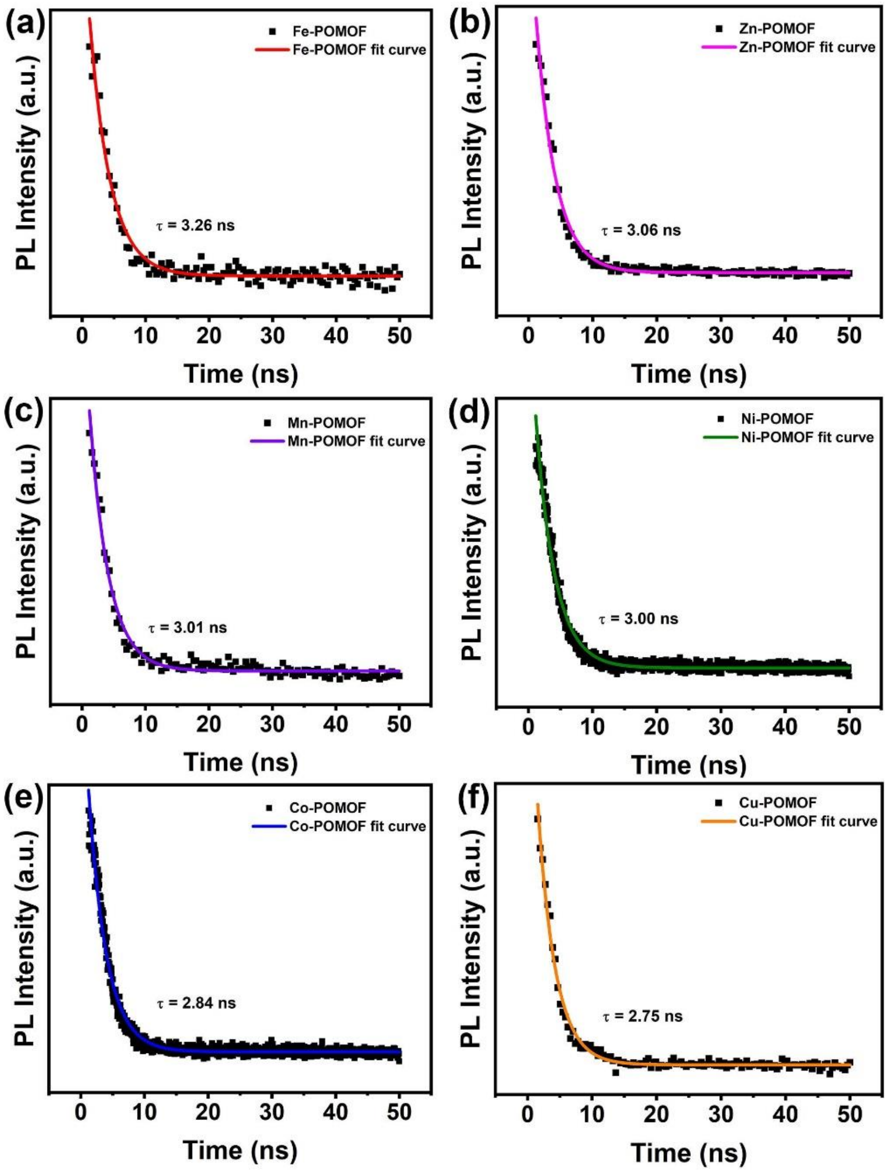

Fig. S18 Transient-state photoluminescence (TSPL) spectroscopy for M-POMOFs. a. Fe-POMOFs (3.26 ns), **b.** Zn-POMOF (3.06 ns), **c.** Mn-POMOF (3.01 ns), **d.** Ni-POMOF (3.0 ns), **e.** Co-POMOF (2.84 ns), **f.** Cu-POMOF (2.75 ns).

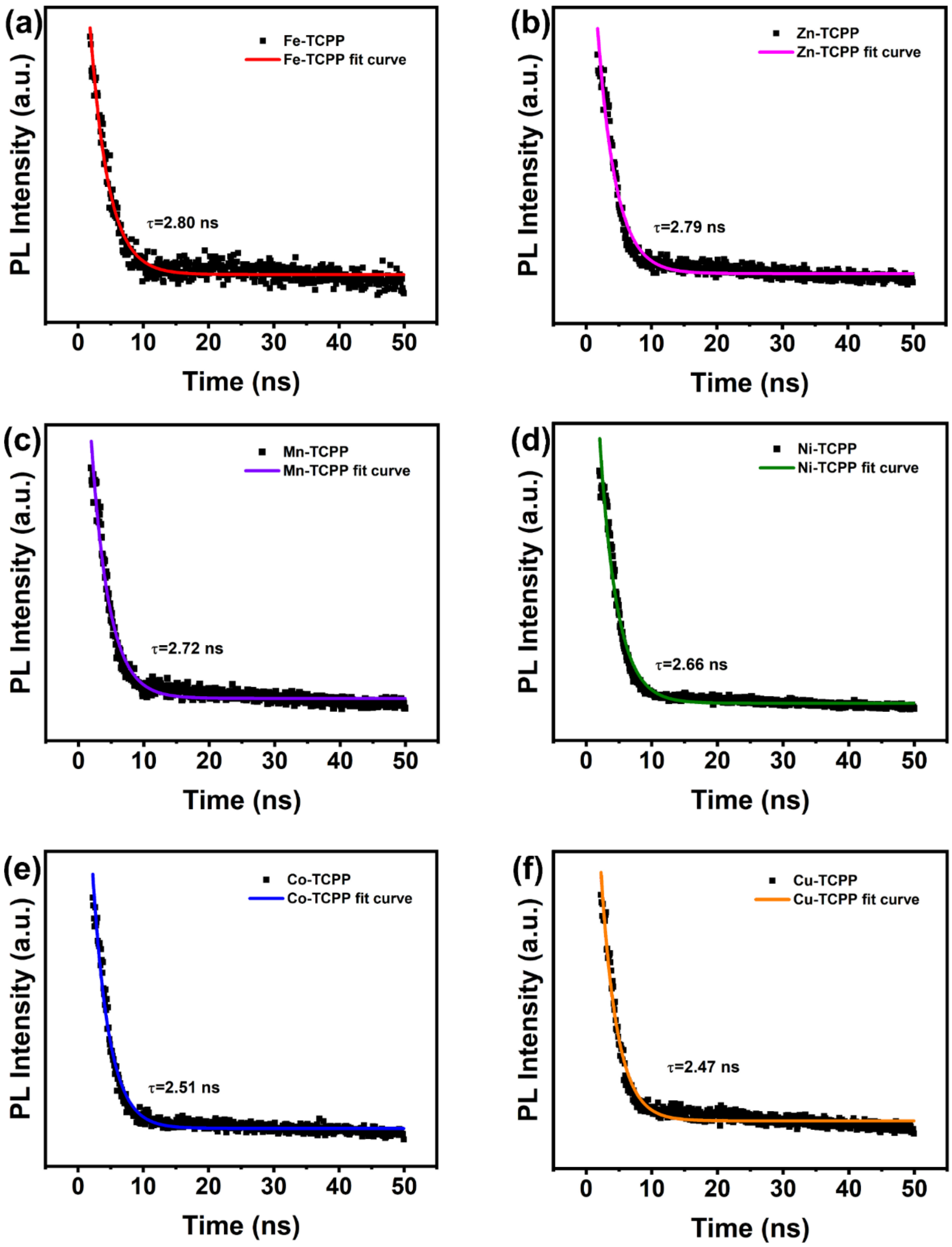

Fig. S19 Transient-state photoluminescence (TSPL) spectroscopy for M-TCPPs. a. Fe-TCPP (2.8 ns), **b.** Zn-TCPP (2.79 ns), **c.** Mn-TCPP (2.72 ns), **d.** Ni-TCPP (2.66 ns), **e.** Co-TCPP (2.51 ns), **f.** Cu-TCPP (2.47 ns).

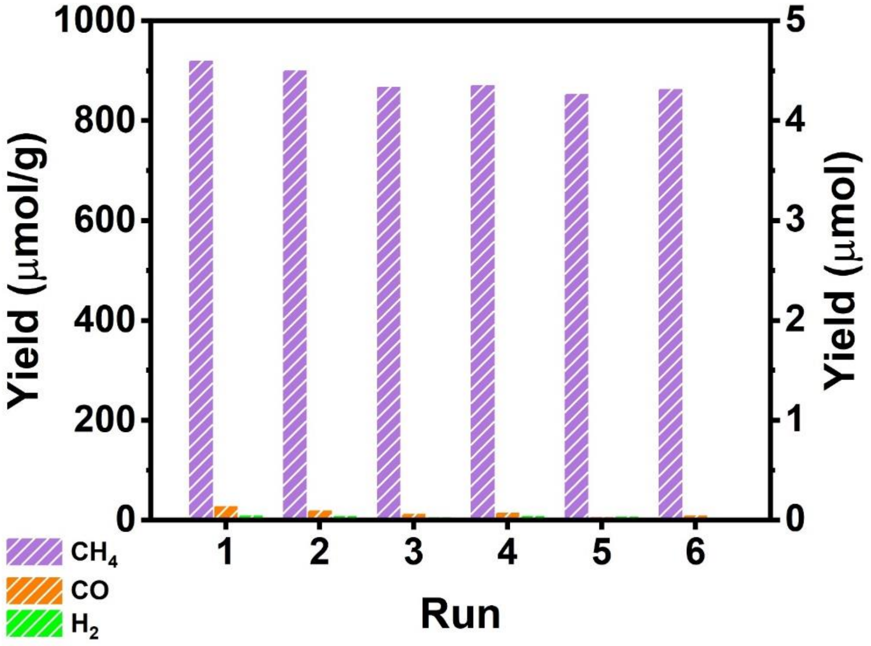

Fig. S20 Production amounts of repeated photocatalytic CO_2 reduction reactions by using Fe-POMOF as catalyst. After each cycle of experiments, the solvent was removed first, and then fresh solvents (28 mL deionized water + 2 mL TEOA) were added, after which pure CO_2 (99.999%) was injected into the system for 30 min. The sealed reaction system (CO_2 pressure of 1 atm) was positioned about 20 cm away from a visible light source. During the catalytic reaction, the photocatalyst particles were kept stirring constantly to ensure adequate contact with CO_2. The reactor was connected with a circulating cooling water system to maintain the temperature at around 20 °C.

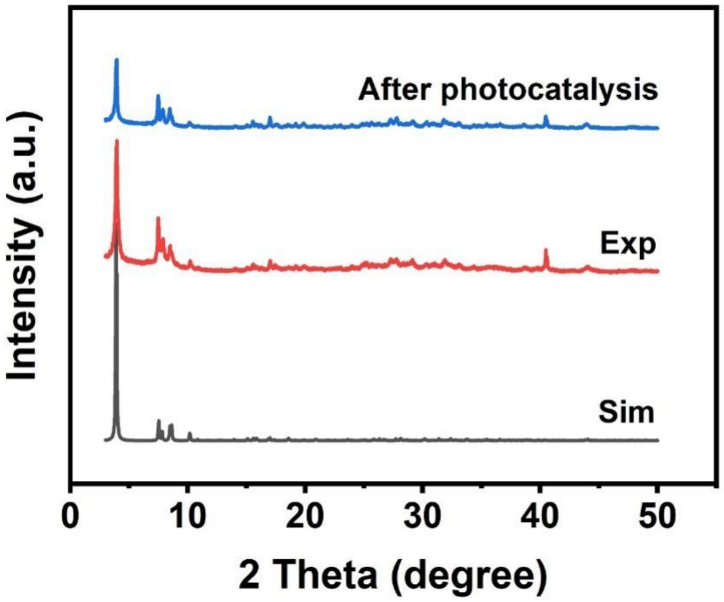

Fig. S21 The PXRD patterns of Fe-POMOF before and after photocatalytic reaction. "Sim" represents the simulated pattern, and "Exp" represents the pattern of as-synthesized sample.

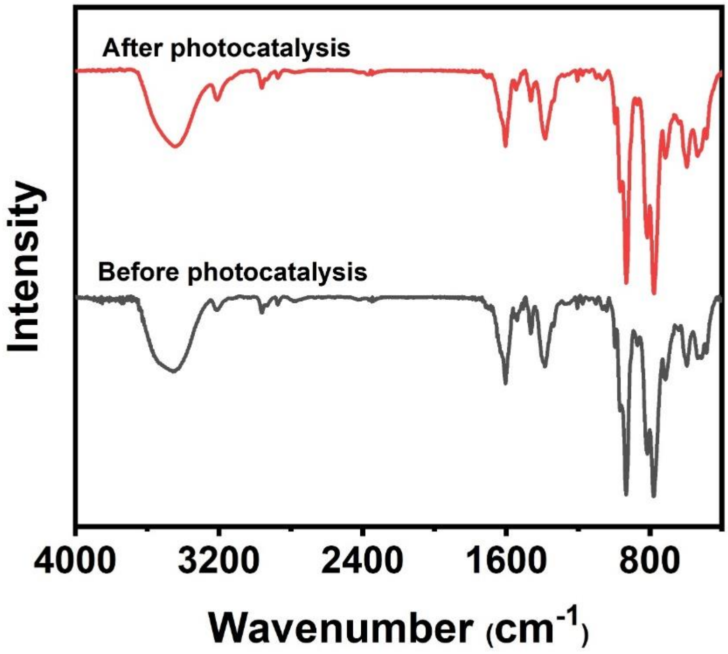

Fig. S22 The FTIR spectra of Fe-POMOF before and after photocatalytic reaction, respectively. The peak at about 1700 cm^{-1} is assigned to the bond in between Zn atom and carboxylic groups (O-C=O) of of TCPP. The peaks in the range of 1620-1420 cm^{-1} are assigned to the porphyrin ring skeleton. As we can see, the FTIR spectra of sample have no noticeable change before and after photocatalytic CO_2 reduction reaction.

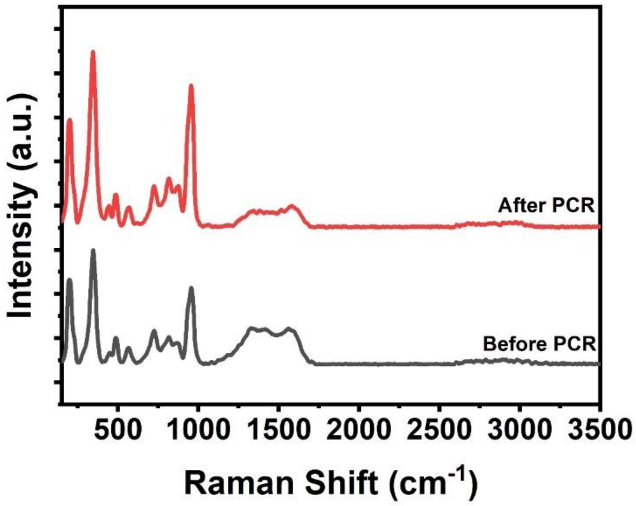

Fig. S23 Raman spectra of Fe-TPMOF before and after PCR reaction.

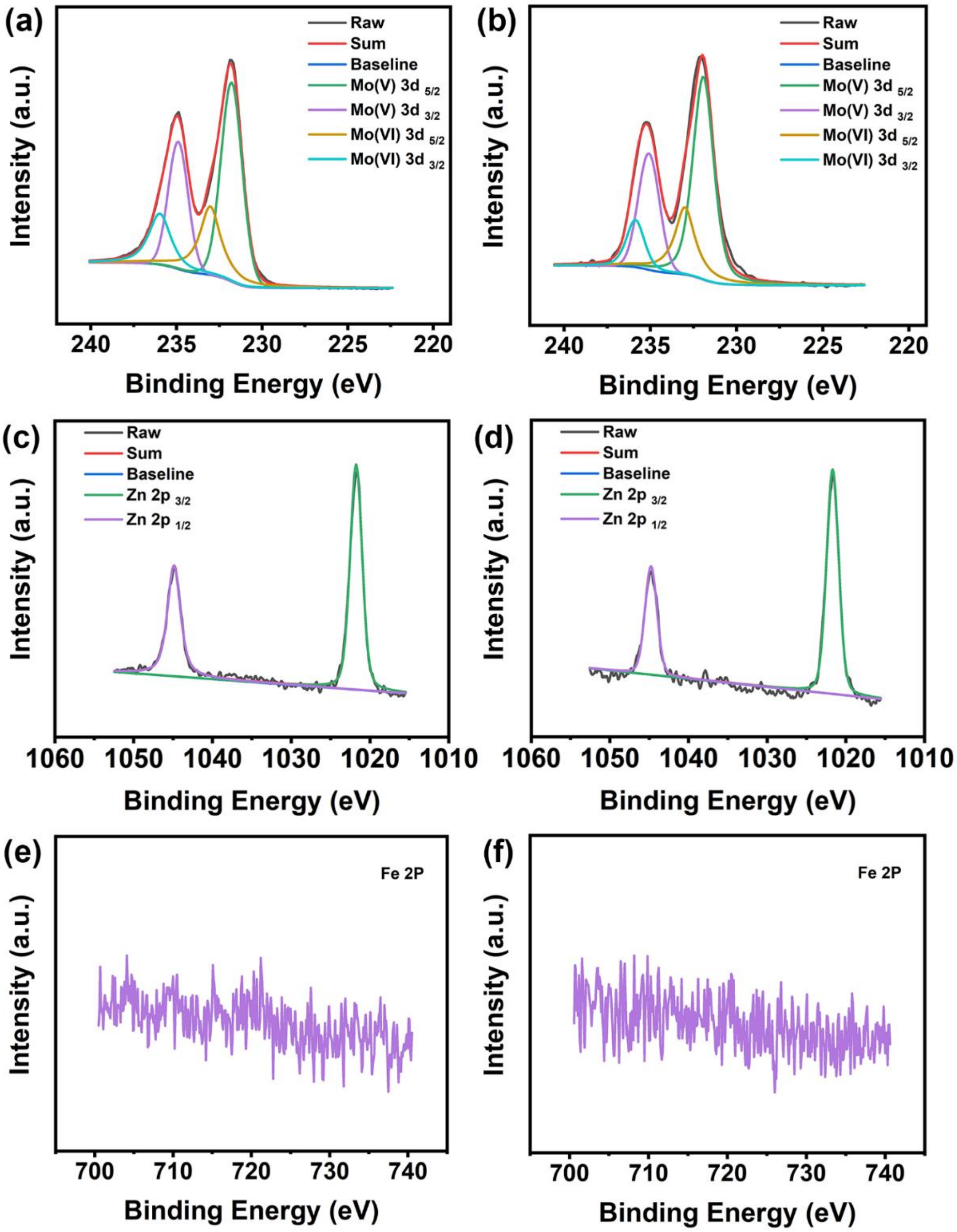

Fig. S24 XPS spectra of metallic elements. **a.** Mo 3d, **c.** Zn 2p and **e.** Fe 2p in Fe-TPMOF before PCR reaction, and for **b.** Mo 3d, **d.** Zn 2p and **f.** Fe 2p in Fe-TPMOF after PCR reaction

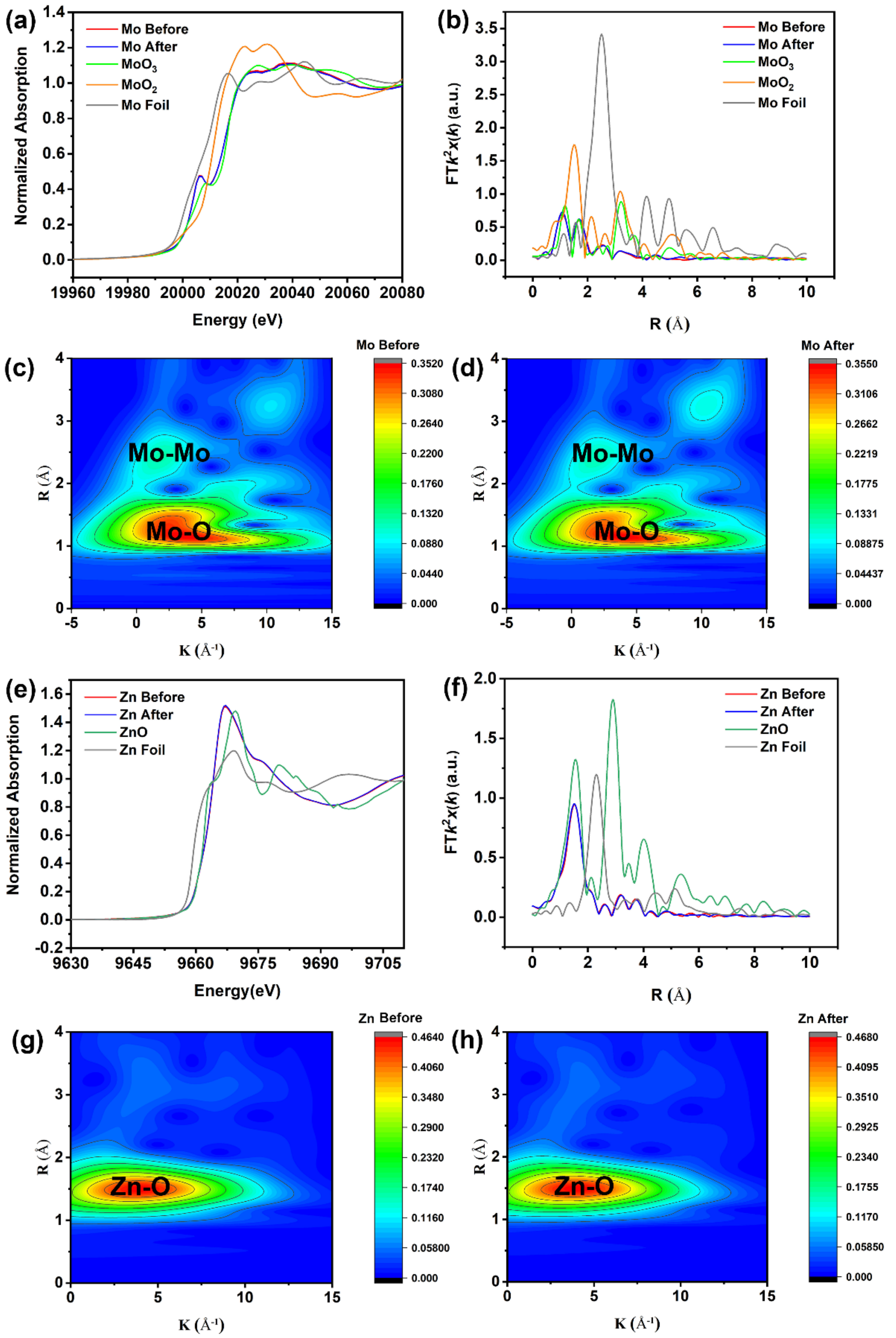

Fig. S25 Synchrotron radiation characterization results of catalyst before and after PCR reaction. **a.** Normalized K-edge X-ray absorption near edge structure (XANES) for Mo in Fe-POMOF before and after PCR reaction. **b.** Radial structure functions (RSFs) of the Mo K-edge obtained by Fourier transformed K^3-weighed EXAFS results. **c.** and **d.** Wavelet transformed EXAFS of Mo in catalyst before and after PCR reaction. **e.** Normalized XANES for Zn in Fe-POMOF before and after PCR reaction. **f.** RSFs of the Zn K-edge obtained by Fourier transformed K^{3-}weighed EXAFS results. **g.** and **h.** Wavelet transformed EXAFS of Zn in catalyst before and after PCR reaction.

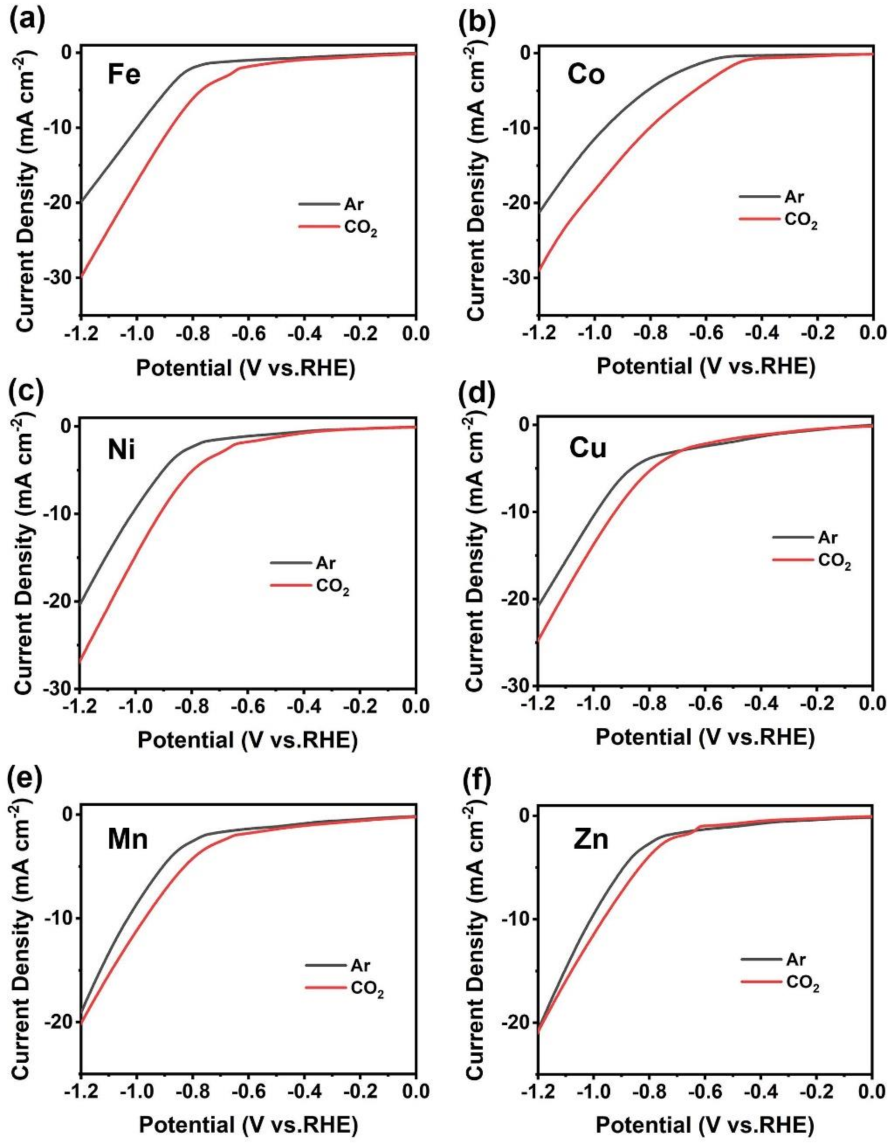

Fig. S26 Linear sweep voltammetric curves. Linear sweep voltammetric curves of M-POMOFs in Ar-saturated and CO_2-saturated 0.5 M $KHCO_3$ aqueous solution.

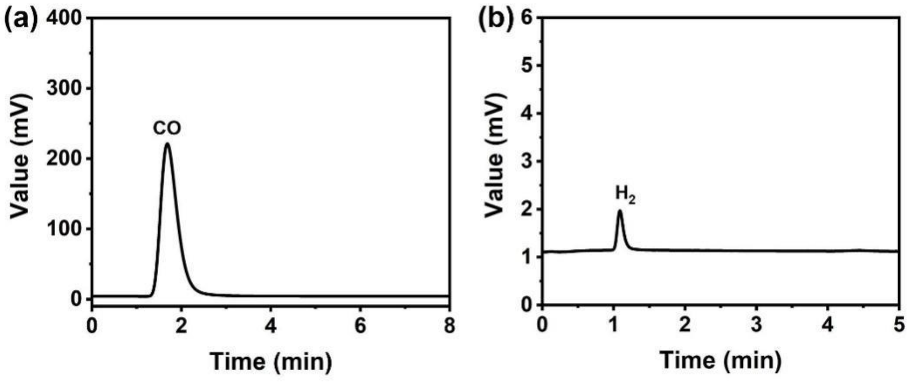

Fig. S27 Gas chromatogram analyses for products in ECR. a. CO and **b.** H_2 were detected by FID and TCD detectors on gas chromatogram.

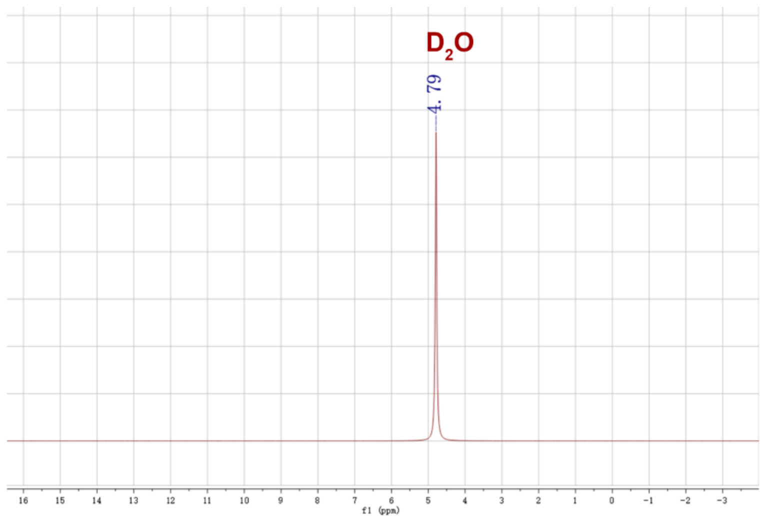

Fig. S28 The ^{1}H NMR spectra of the liquid phases after the ECR experiment for Fe-POMOF.

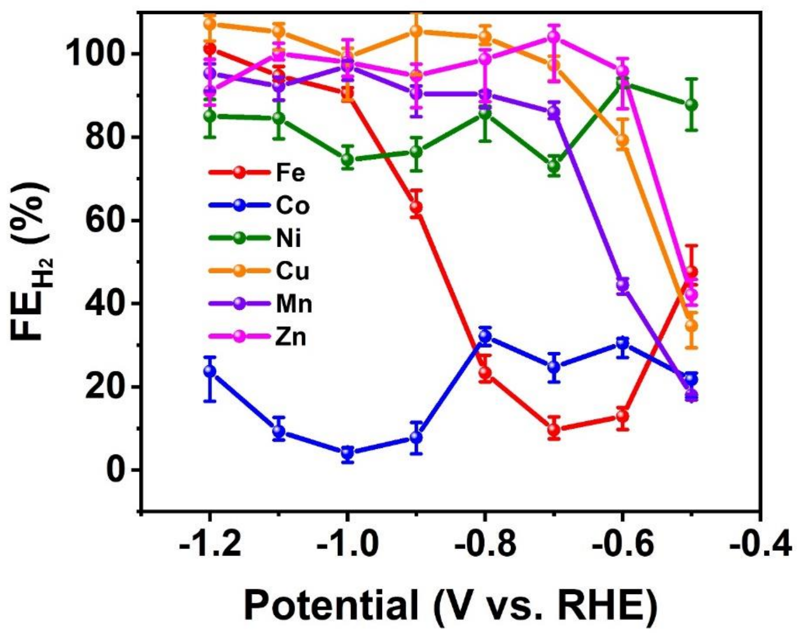

Fig. S29 Faradaic efficiencies for H_2. FE(H_2) of M-POMOFs at different applied potentials under CO_2-saturated 0.5 M $KHCO_3$ aqueous solution.

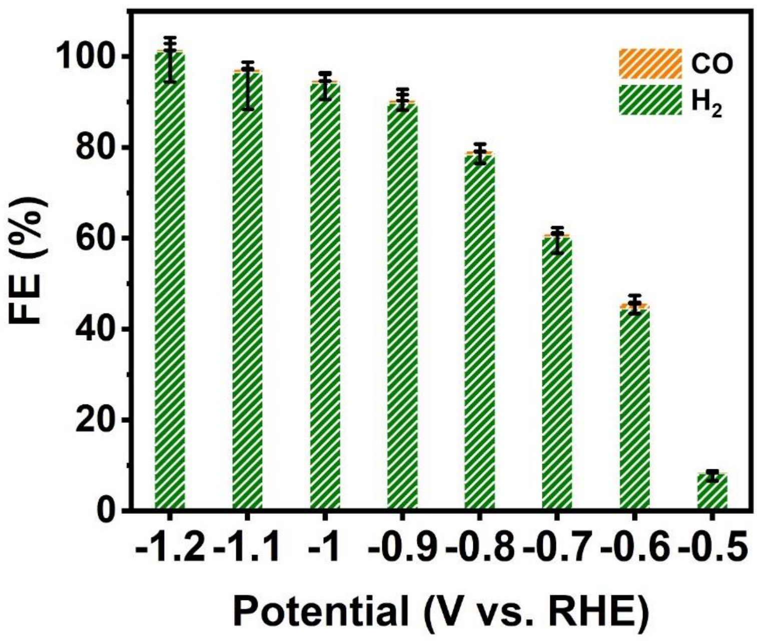

Fig. S30 Faradaic efficiencies for CO and H_2. Faradaic efficiencies for H_2 and CO of conductive substrate (acetylene black on carbon paper) at different applied potentials under CO_2-saturated 0.5 M $KHCO_3$ aqueous solution.

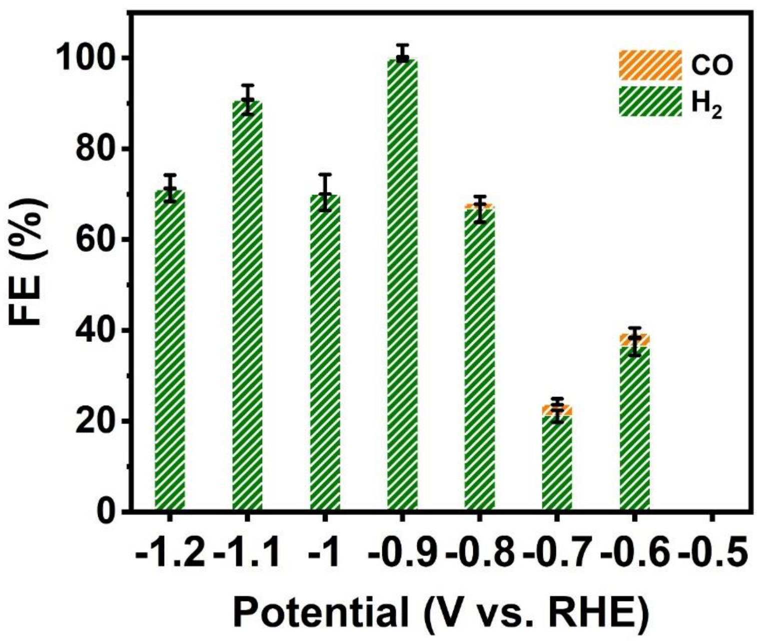

Fig. S31 Faradaic efficiencies for H_2 and CO. Faradaic efficiencies for H_2 and CO of Fe-POMOFs at different applied potentials under Ar-saturated 0.5 M $KHCO_3$ aqueous solution.

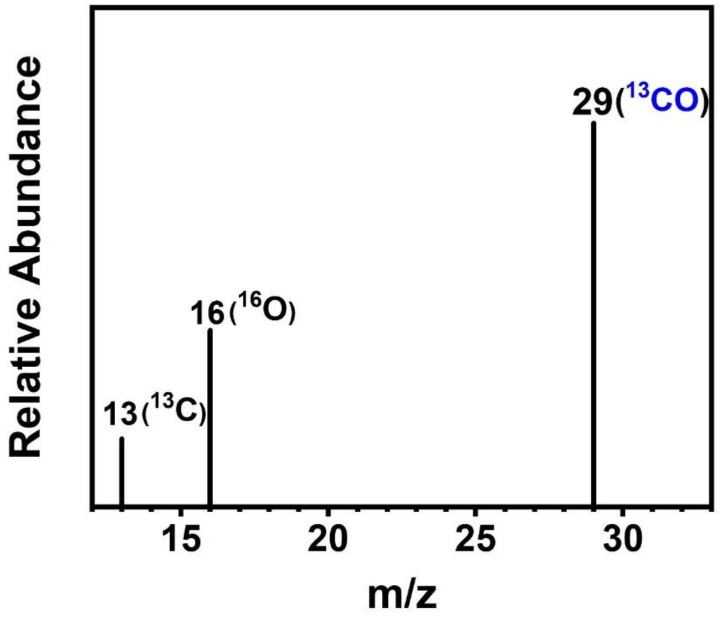

Fig. S32 Gas chromatography and mass spectra (GC-MS) analyses. GC-MS of ^{13}CO generation using Fe-POMOF as photocatalyst in photoreduction reaction under $^{13}CO_2$ atmosphere (The single ion monitoring (SIM) mode of mass spectrometry).

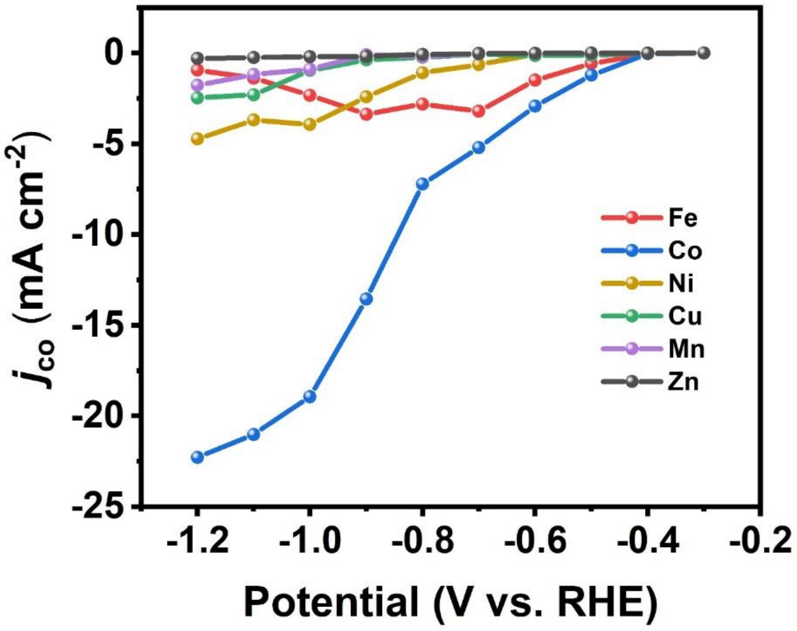

Fig. S33 Partial current density for CO. Partial current density (based on geometric surface area) plots of M-POMOFs for CO in CO_2-saturated 0.5 M $KHCO_3$ aqueous solution.

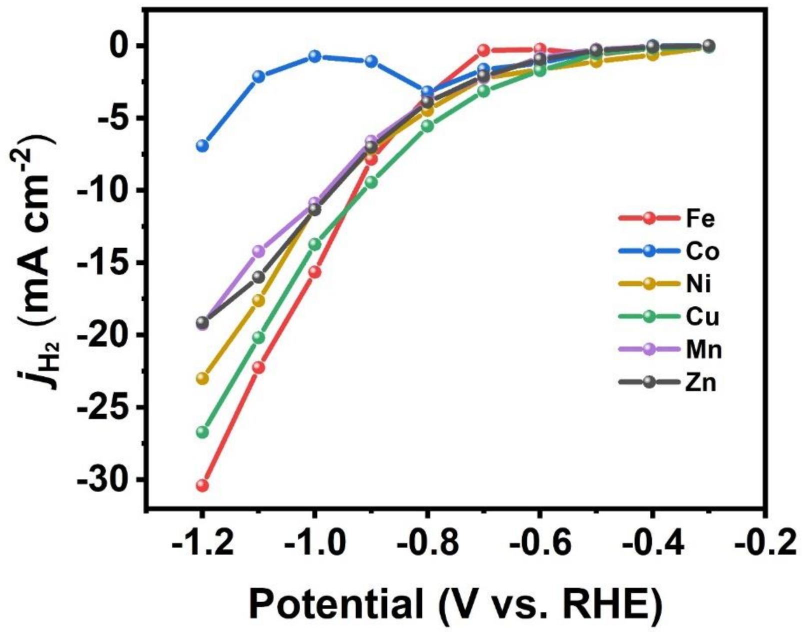

Fig. S34 Partial current density for H_2. Partial current density (based on geometric surface area) plots of M-POMOFs for H_2 in CO_2-saturated 0.5 M $KHCO_3$ aqueous solution.

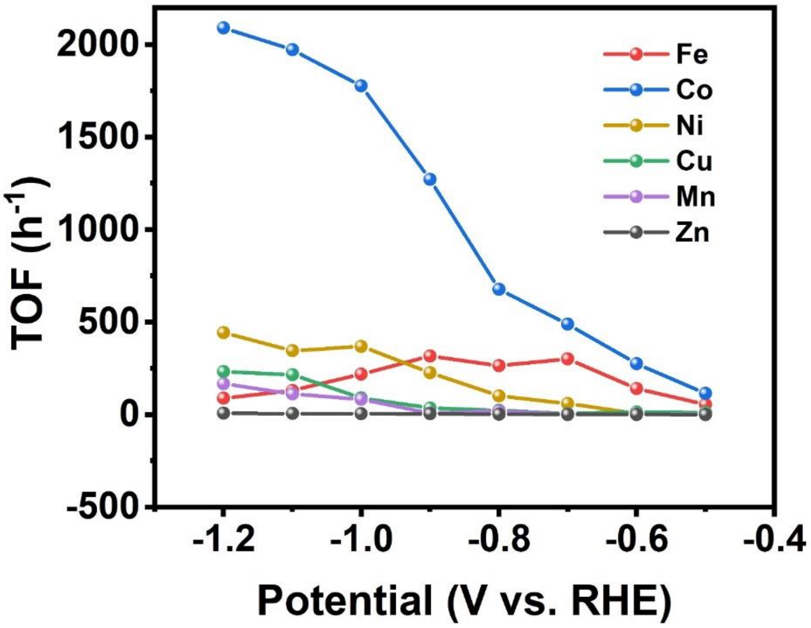

Fig. S35 TOF of M-POMOFs in CO_2-saturated 0.5 M $KHCO_3$ aqueous solution.

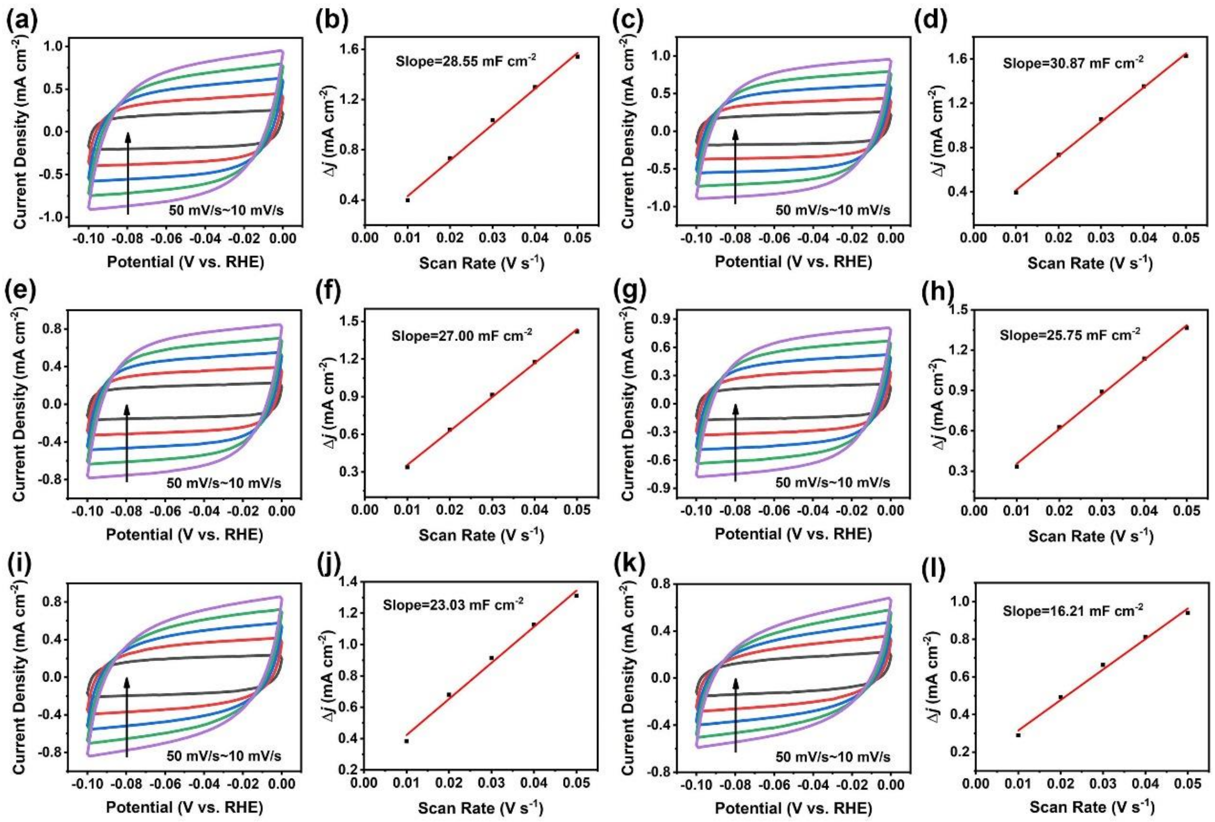

Fig. S36 Cyclic voltammetry (CV) curves in the region of 0.00 ~ 0.10 V *vs*. RHE at various scan rate (50 ~ 10 mV s^{-1}) and corresponding capacitive current at -0.05 V as a function of scan rate. (**a-b**) Fe-POMOF, (**c-d**) Co-POMOF, (**e-f**) Ni-POMOF, (**g-h**) Cu-POMOF, (**i-j**) Mn-POMOF, (**k-l**) Zn-POMOF.

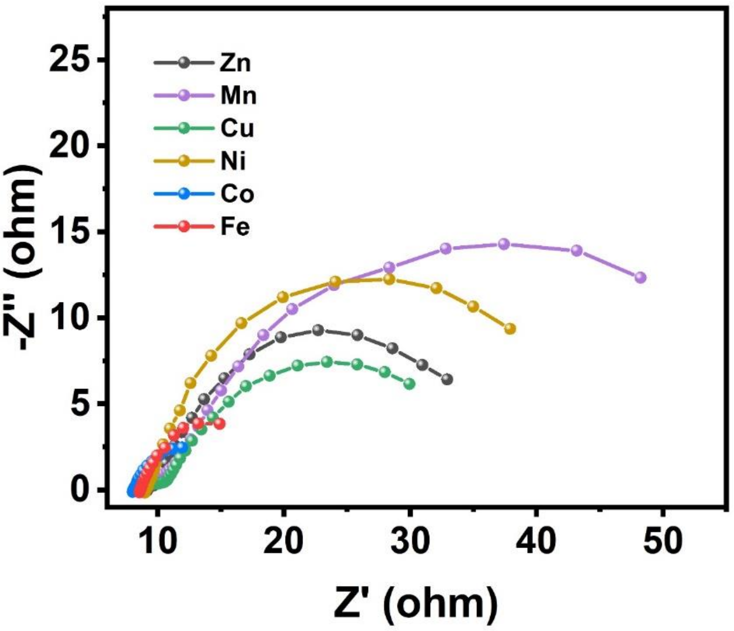

Fig. S37 Nyquist plots of M-POMOFs catalysts over the frequency ranging from 100 kHz to 0.1 Hz.

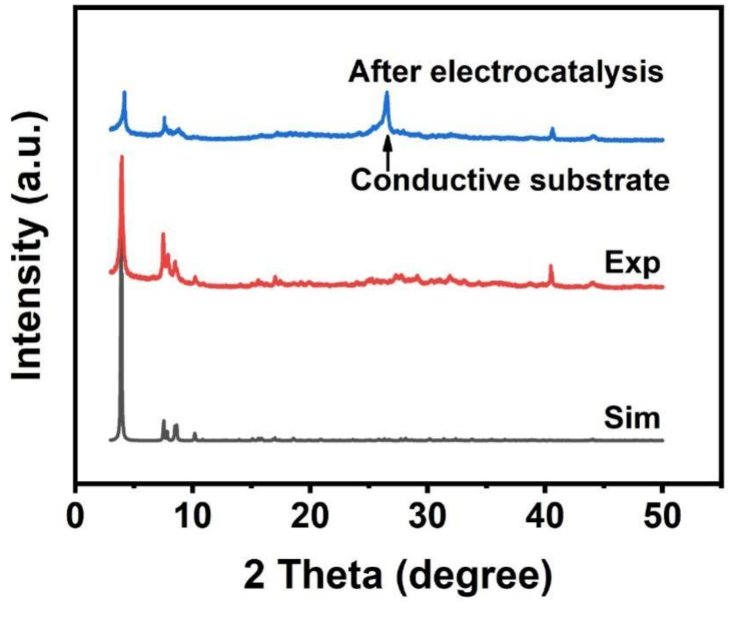

Fig. S38 PXRD patterns of Fe-POMOF after electrochemical experiment. "Sim": simulated pattern and "Exp": as-synthesized sample. The PXRD pattern of Fe-POMOF after electrochemical experiment matches well with the simulated and as-synthesized samples.

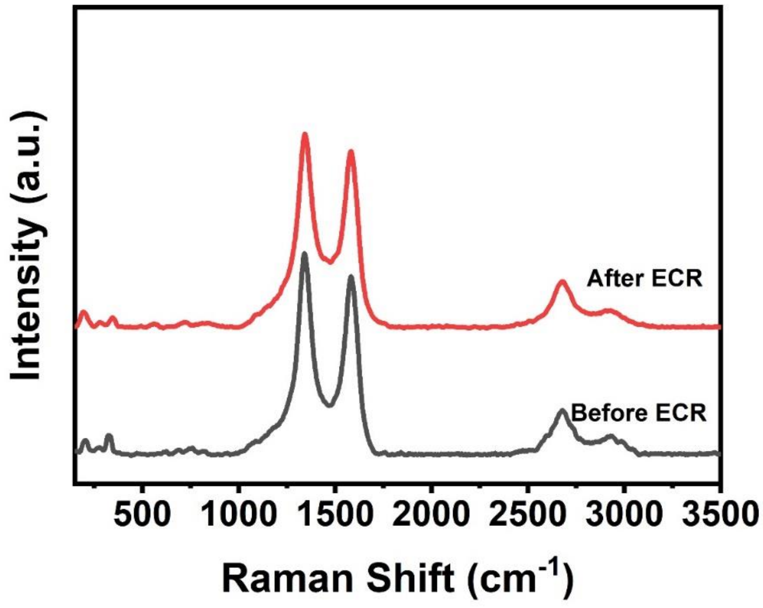

Fig. S39 Raman spectra of Fe-TPMOF before and after ECR reaction. Different from the photocatalytic conditions, the working electrode was added acetylene black (POMOF: acetylene black = 1:1) in order to enhance the conductivity of the catalyst.

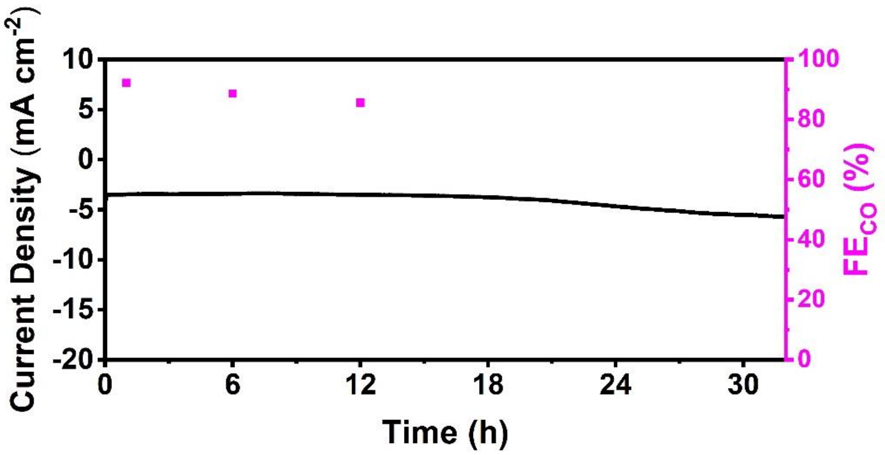

Fig. S40 Durability test of Fe-POMOF at the potential of −0.7 V vs. RHE.

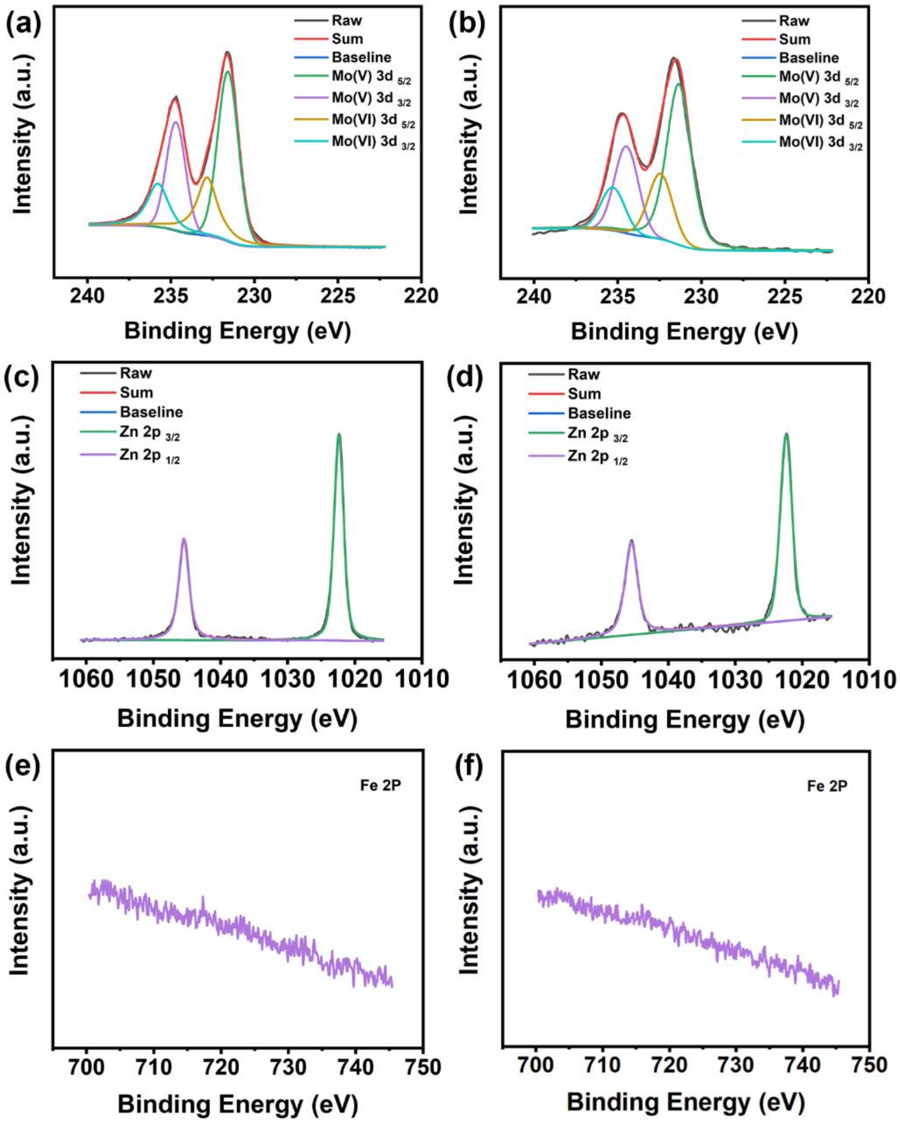

Fig. S41 XPS spectra of metallic elements before and after ECR reaction. a. Mo 3d, **c.** Zn 2p and **e.** Fe 2p in Fe-TPMOF before ECR reaction, and for **b.** Mo 3d, **d.** Zn 2p and **f.** Fe 2p in Fe-TPMOF after ECR reaction.

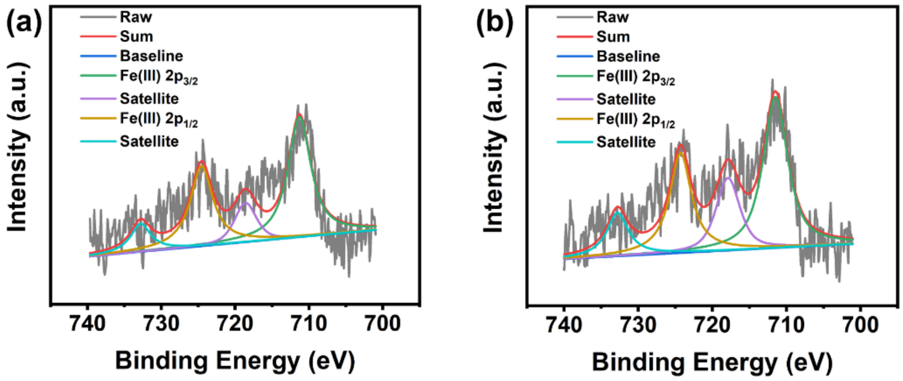

Fig. S42 XPS spectra of Fe in Fe-TCPP before and after ECR reaction. a. XPS spectra of Fe 2p in Fe-TCPP before ECR reaction. **b.** XPS spectra of Fe 2p in Fe-TCPP after ECR reaction. The detection limit of XPS usually required the mass percentage of the target metal higher than 1%, and that of Fe in Fe-POMOF was measured to be ~0.25%. Since the Fe content in Fe-POMOF was less than 1%, XPS detection had almost no signal. After we confirmed the good stability of the framework, we further confirmed the valence of Fe in the Fe-TCPP before and after the ECR reaction, which reflected valence variation of Fe in Fe-POMOF due to the same environment of Fe. As shown in the results, the valence state of Fe (III) remained the same before and after the ECR reaction.

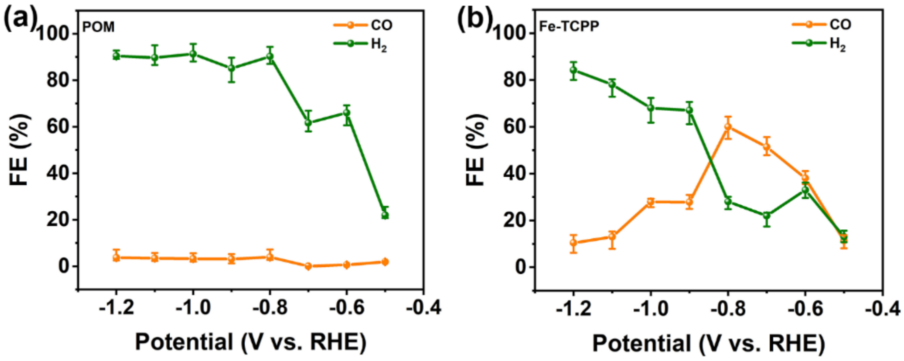

Fig. S43 Faradaic efficiencies at different applied potentials in CO_2-saturated 0.5 M $KHCO_3$ aqueous solution. a. POM (Zn-ε-Keggin). **b.** Fe-TCPP.

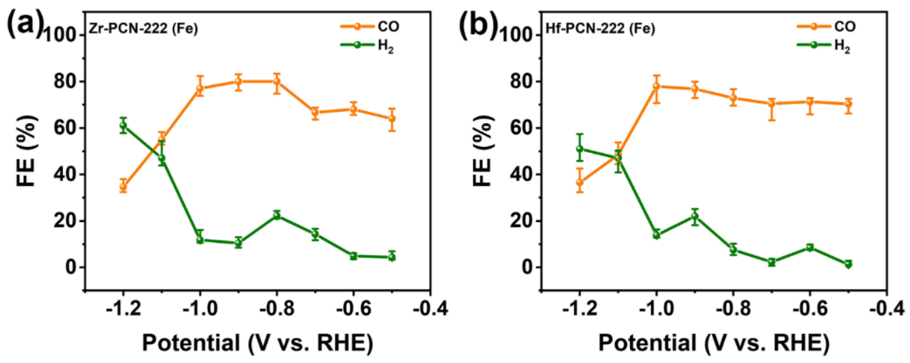

Fig. S44 Faradaic efficiencies of Fe-MOFs at different applied potentials in CO_2-saturated 0.5 M $KHCO_3$ aqueous solution. a. Zr-PCN-222(Fe). **b.** Hf-PCN-222(Fe).

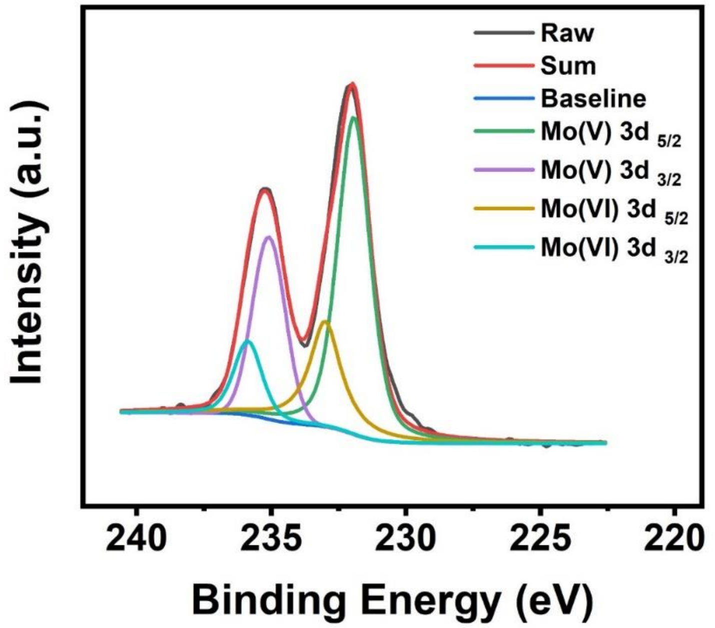

Fig. S45 *In situ* XPS signals of Mo 3d after PCR reaction.

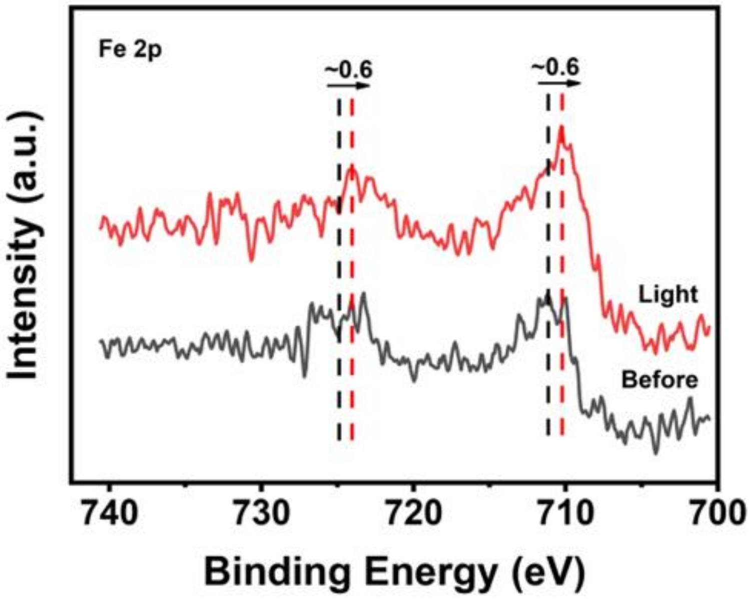

Fig. S46 *In situ* XPS signals of Fe 2p for Fe-TCPP before and under light. The detection limit of XPS usually required the mass percentage of the target metal higher than 1%, and that of Fe in Fe-POMOF was measured to be ~0.25%. Since the Fe content in Fe-POMOF was less than 1%, the XPS detection had almost no signal and the binding energy change of Fe in Fe-POMOF could not be measured. Instead, the binding energy of Fe in Fe-TCPP was measured due to the same environment of Fe in both Fe-TCPP and Fe-POMOF. The *in situ* XPS showed that the binding energy of Fe in Fe-TCPP decreased slightly upon illumination. This may be a similar case for the binding energy change of Fe in Fe-POMOF due to the same environment of Fe.

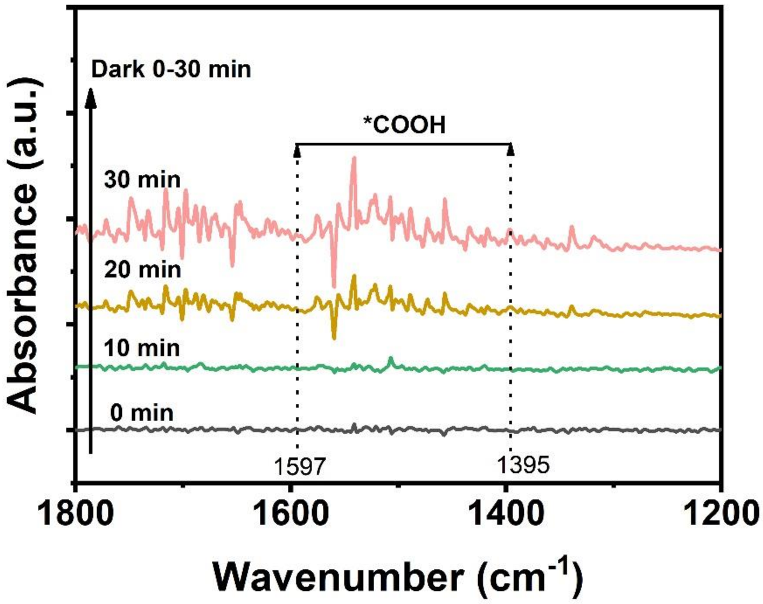

Fig. S47 *In situ* FTIR measurement on Fe-POMOF catalyst in the dark.

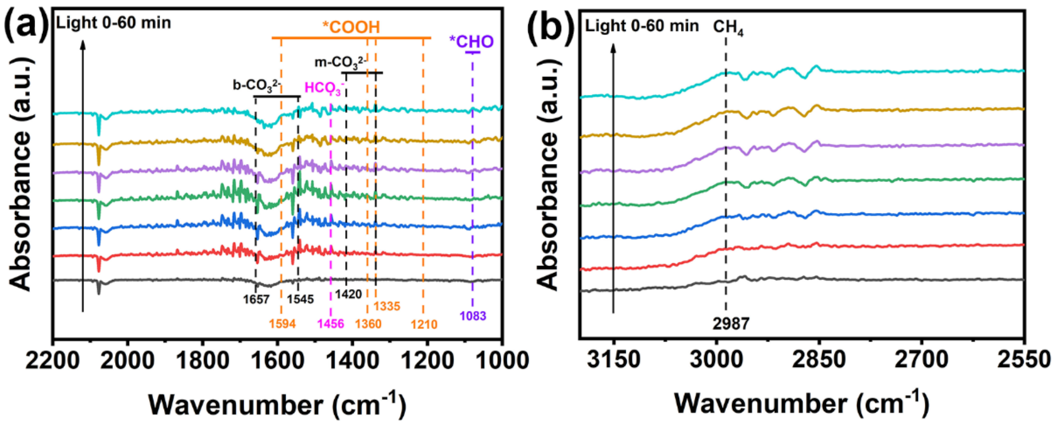

Fig. S48 *In situ* FTIR measurement using POM as a catalyst for PCR reaction. a. 2200-1000 cm^{-1}; **b.** 3200-2550 cm^{-1}. The IR peaks at 1545 and 1657 cm^{-1} were assigned to bidentate carbonate (b-CO_3^{2-}) species on POM; the new Peaks at 1335 and 1420 cm^{-1} were corresponded to monodentate carbonate (m-CO_3^{2-}), Peaks at 1456 cm^{-1} was assigned to HCO_3^-. The occurrence of m-, b-CO_3^{2-} and HCO_3^- indicate that CO_2 can be adsorbed on the surface of the catalysts. Peaks appearing at 1210, 1335, 1360, and 1594 cm^{-1} were ascribed to the formation of *COOH, which was a crucial intermediate during the photochemical conversion of CO_2. Meanwhile, the presence of the IR peak at 1083 cm^{-1}, assigned to *CHO, indicated the formation of another pivotal intermediate species for the CH_4. Decisively, the peak at 2987 cm^{-1} could be corresponded to CH_4. Therefore, the whole process of the reduction could be described as that POM firstly adsorbed CO_2, and then reduce the adsorbed CO_2 through the key intermediates (e.g. *COOH, *CHO), and finally transformed them into CH_4.

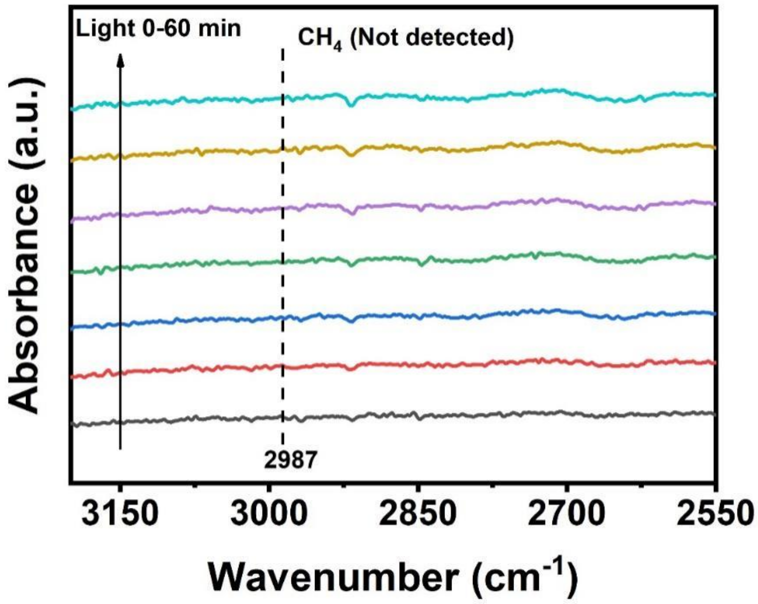

Fig. S49 ***In situ*** **FTIR measurement using Fe-TCPP as a catalyst for PCR reaction.**

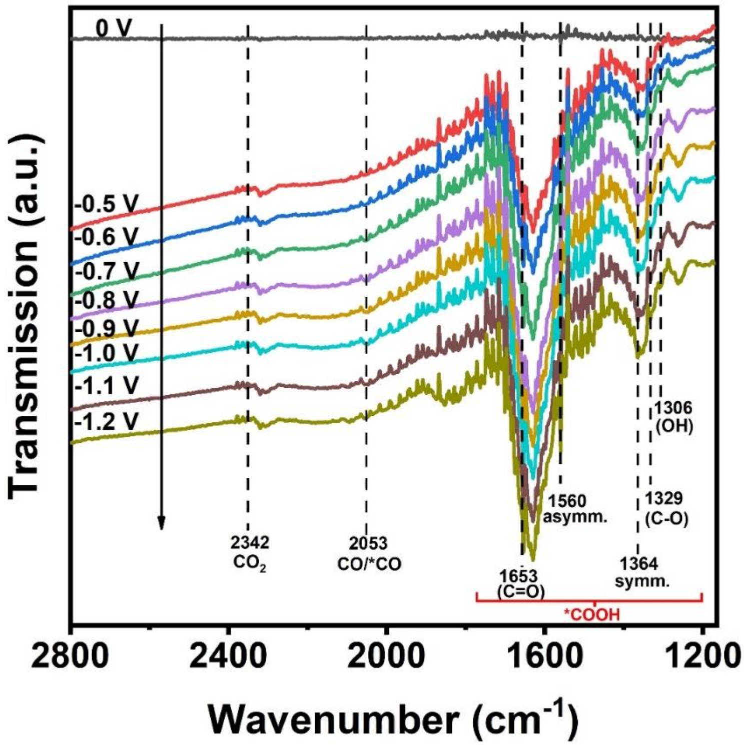

Fig. S50 ***In situ*** **FTIR measurement using Fe-TCPP as a catalyst for ECR reaction.** Peaks appearing at 1306, 1329, 1364, 1560 and 1653 cm^{-1} were ascribed to the formation of *COOH, which was a crucial intermediate for CO_2 reduction reaction. Meanwhile, the presence of the IR peak at 2053 cm^{-1}, assigned to CO/*CO, indicated the formation of another pivotal intermediate species for the CO. The peak at 2342 cm^{-1} could be corresponded to CO_2.

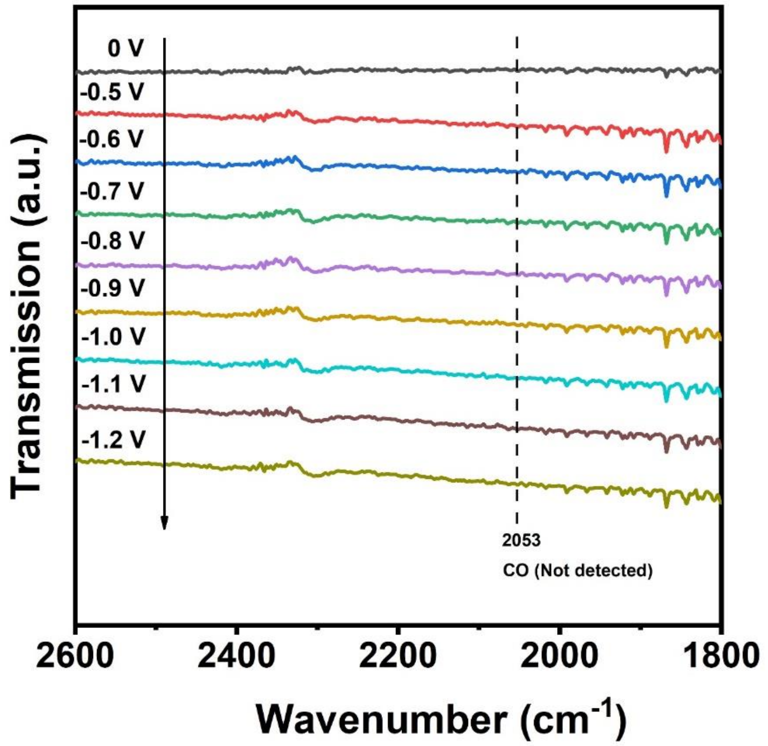

Fig. S51 *In situ* FTIR measurement using POM as a catalyst for ECR reaction.

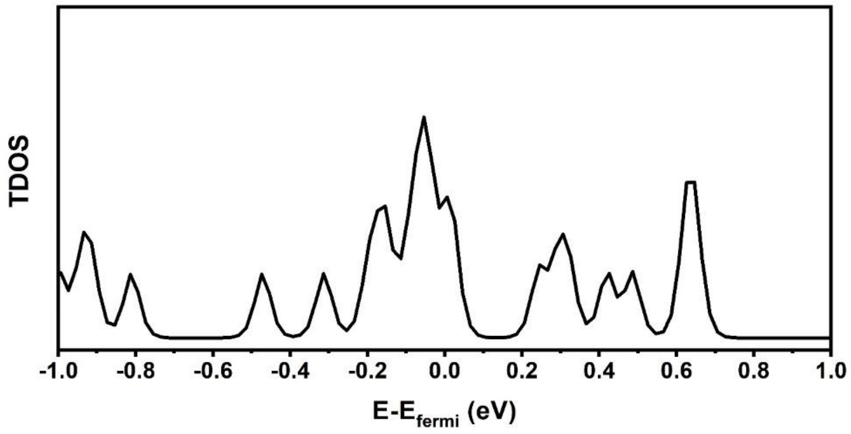

Fig. S52 Total density of states (TDOS) of Fe-POMOF.

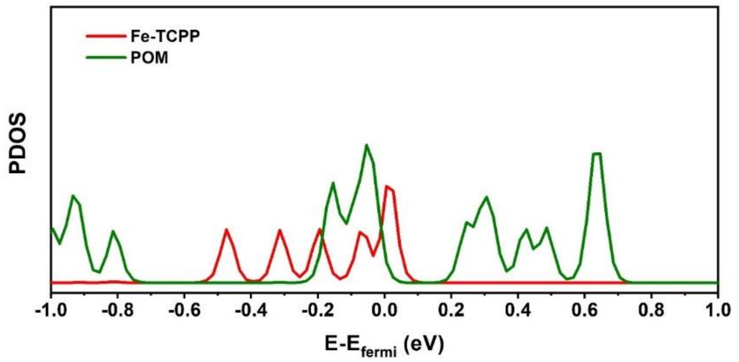

Fig. S53 Partial density of states (PDOS) of Fe-TCPP and POM in Fe-POMOF.

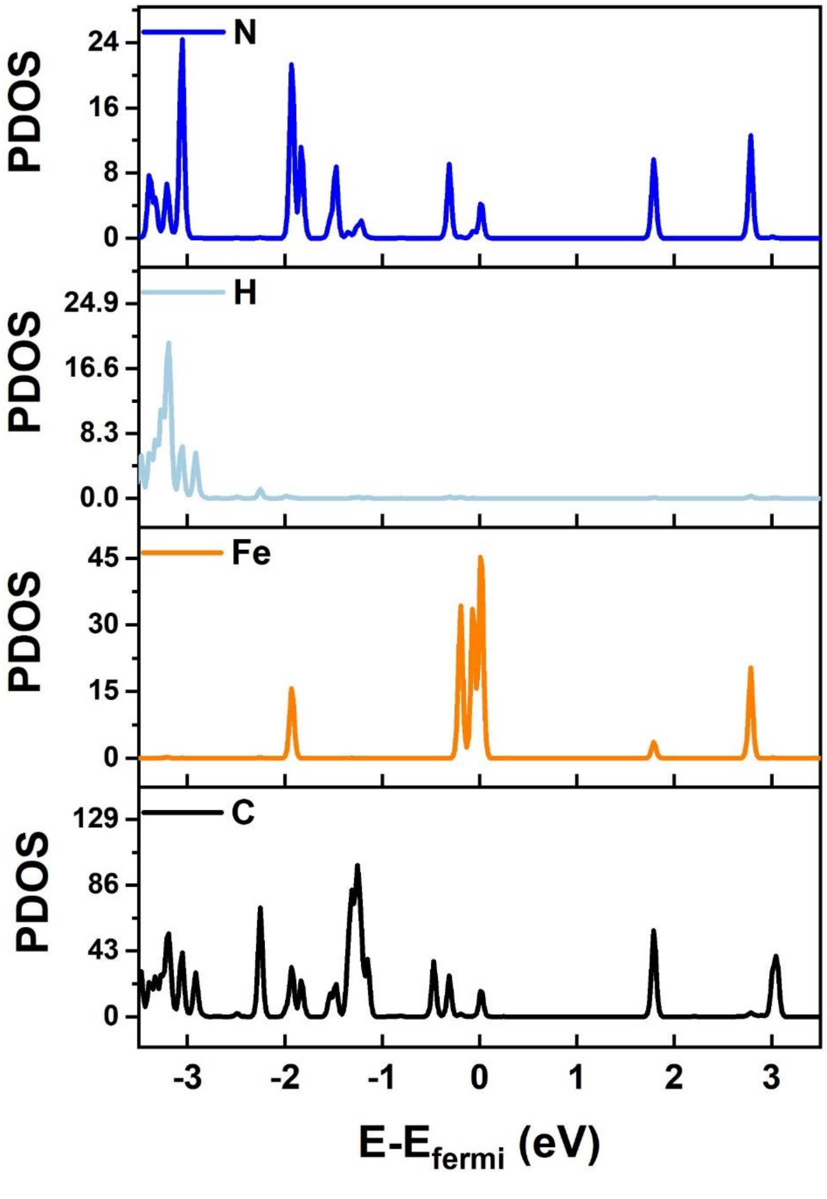

Fig. S54 Partial density of states (PDOS) of N, H, Fe, N elements belong to Fe-TCPP ligand in Fe-POMOF.

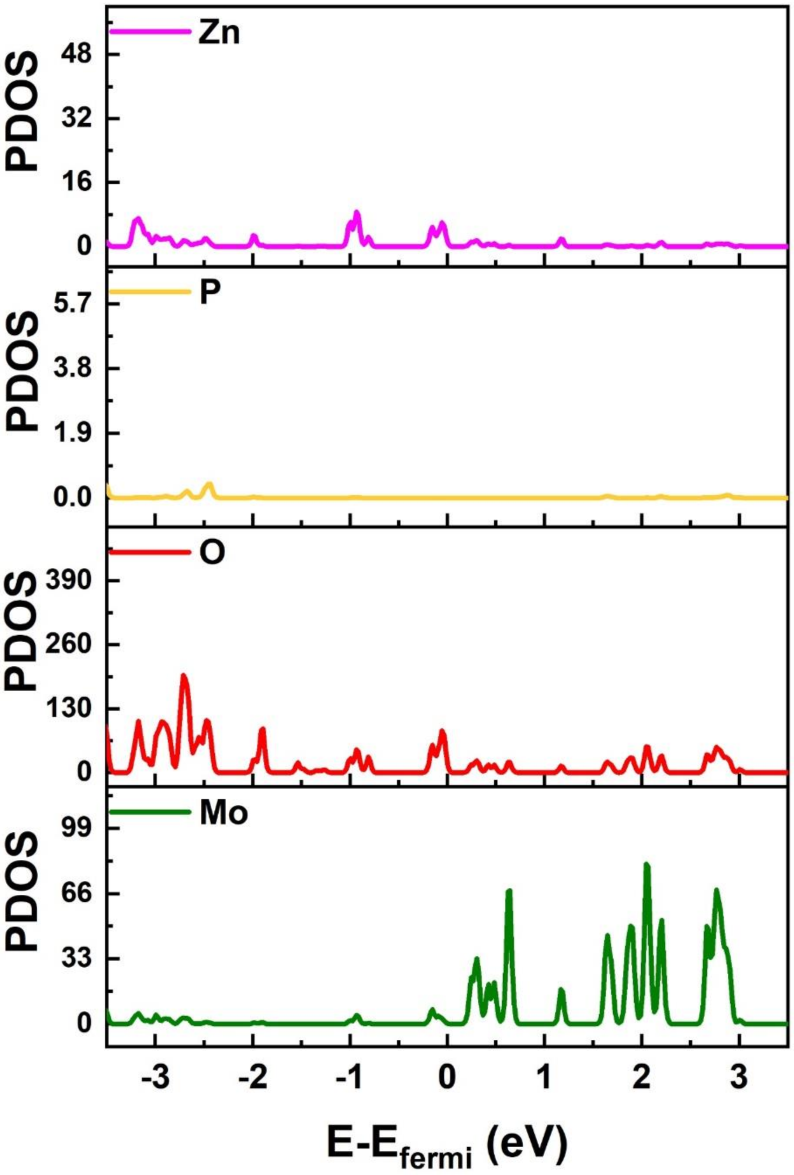

Fig. S55 Partial density of states (PDOS) of Zn, P, O, Mo elements belong to POM ligand in Fe-POMOF.

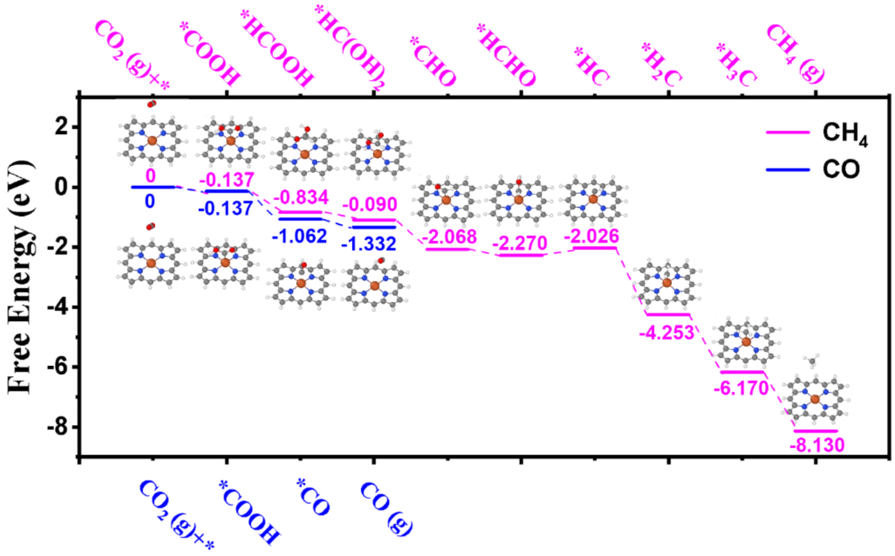

Fig. S56 The optimized structures for reaction intermediates on Fe-TCPP in photocatalysis reaction (when the LUMO of Fe-POMOF is -0.88 V vs. RHE).

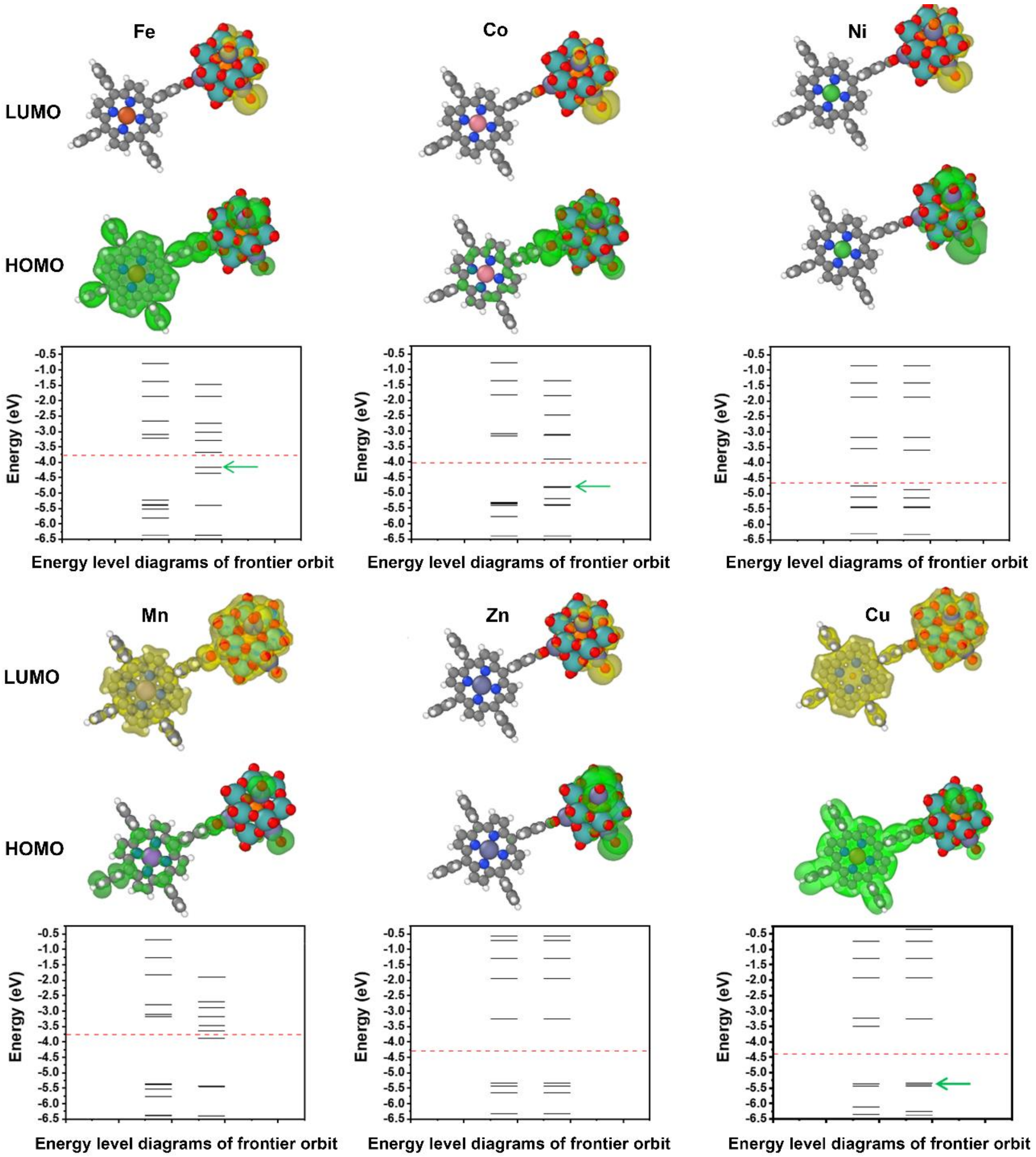

Fig. S57 The frontier orbitals of the associated materials. The orbital recorded was from HOMO-5 to LUMO+5. The red dashed lines were associated with Fermi energy. In each diagram, the left and right short lines indicate the orbitals for spin-up and down, respectively.

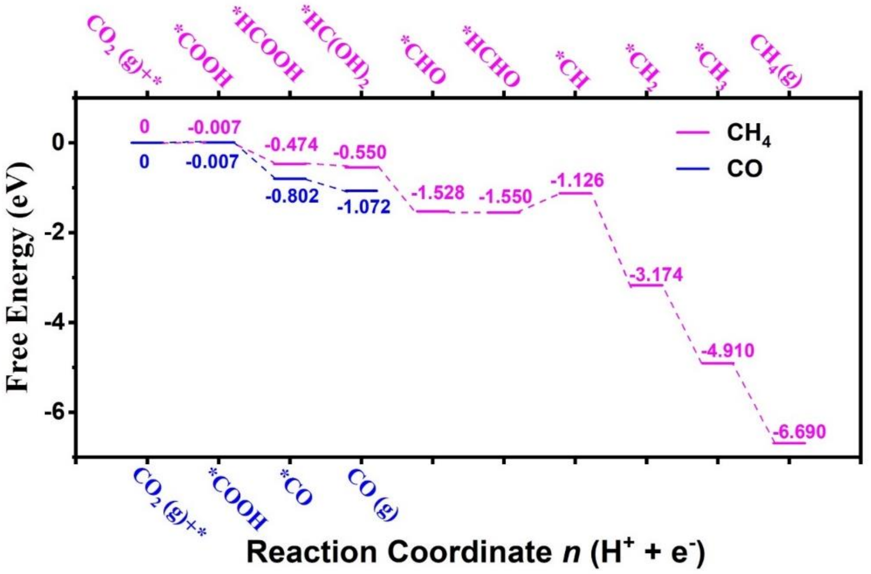

Fig. S58 The optimized structures for reaction intermediates on Fe-TCPP of Fe-POMOF in ECR reaction (when the reduction potential is -0.7 V).

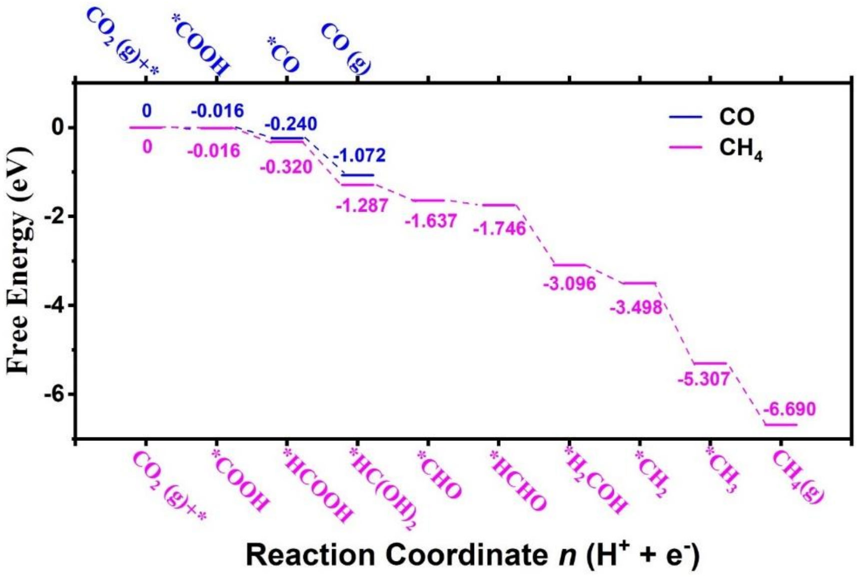

Fig. S59 The optimized structures for reaction intermediates on POM of Fe-POMOF in ECR reaction (when the reduction potential is -0.7 V).

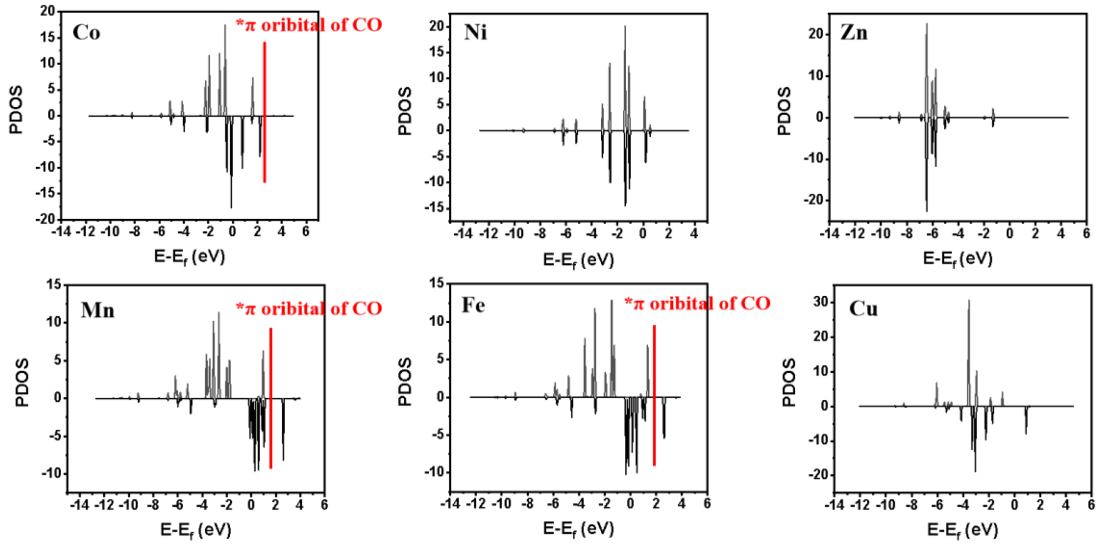

Fig. S60 The black plots are PDOS of the metal centers in M-TCPP. The red lines indicate the *π orbital of CO.

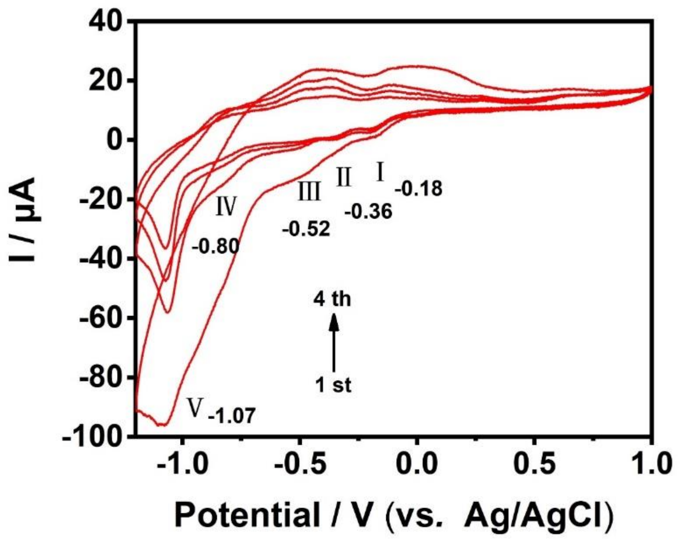

Fig. S61 CVs of Fe-POMOF were measured in CO_2-saturated 0.5 mol·L^{-1} $KHCO_3$ aqueous solution (ECR reaction) at 40 mV·s^{-1}. The peaks of I, II, III were the characteristic peaks of POM in Fe-POMOF. The peaks of IV and V were the characteristic peaks of Fe in Fe-POMOF.

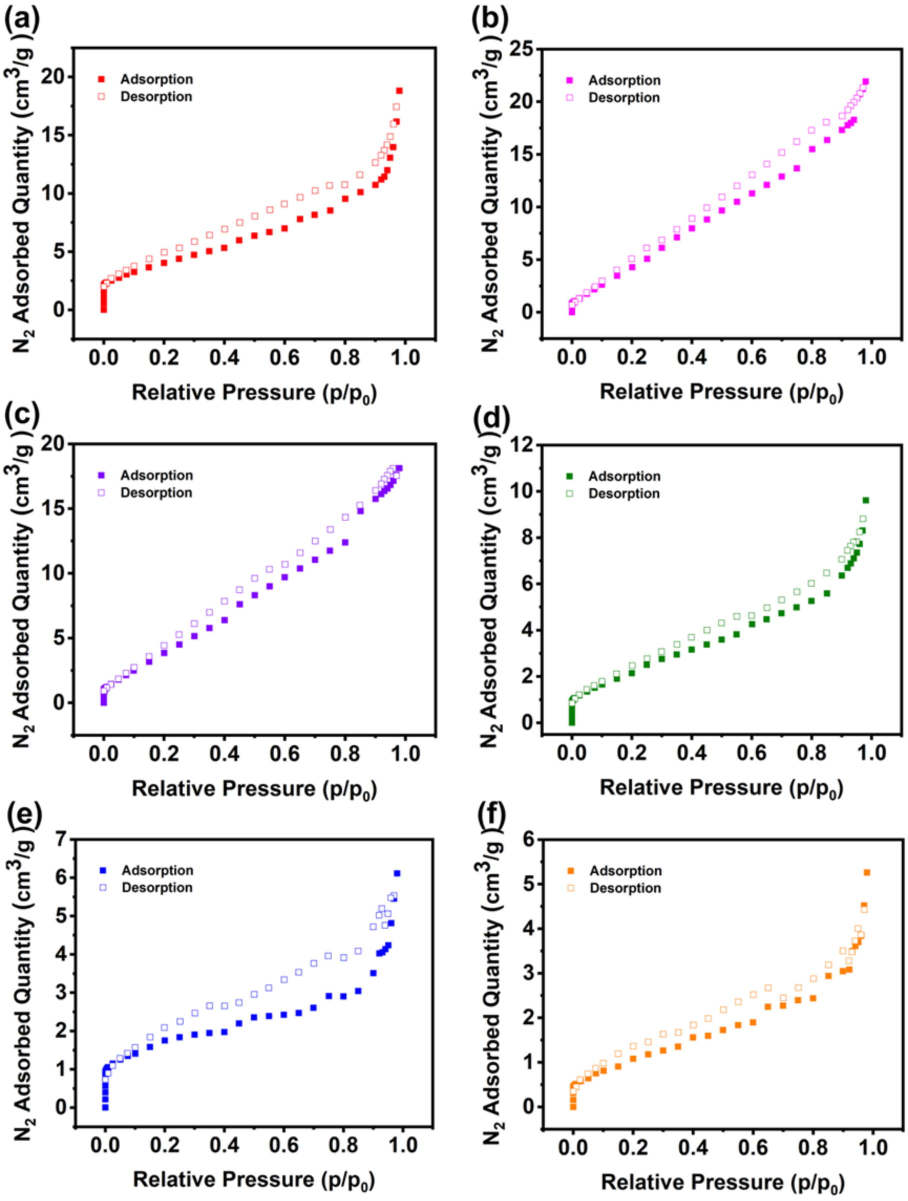

Fig. S62 N_2 adsorption isotherms for M-POMOFs at 77 K. a. Fe-POMOF, b. Zn-POMOF, c. Mn-POMOF, d. Ni-POMOF, e. Co-POMOF, f. Cu-POMOF. The N_2 uptakes of M-POMOFs were measured to be 19 cm^3 g^{-1}(Fe), 21.9 cm^3 g^{-1}(Zn), 18.1 cm^3 g^{-1}(Mn), 9.6 cm^3 g^{-1}(Ni), 6.1 cm^3 g^{-1}(Co), 5.2 cm^3 g^{-1}(Cu). The experimental pore volumes of M-POMOFs were measured to be 0.03 cm^3 g^{-1}(Fe), 0.035 cm^3 g^{-1}(Zn), 0.028 cm^3 g^{-1}(Mn), 0.015 cm^3 g^{-1}(Ni), 0.009 cm^3 g^{-1}(Co), 0.008 cm^3 g^{-1}(Cu). The Brunauer-Emmett-Teller (BET) surface areas for M-POMOFs were 15.1 m^2 g^{-1}(Fe), 23.8 m^2 g^{-1}(Zn), 20 m^2 g^{-1}(Mn), 8.2 m^2 g^{-1}(Ni), 6.4 m^2 g^{-1}(Co), 4.1 m^2 g^{-1}(Cu), respectively.

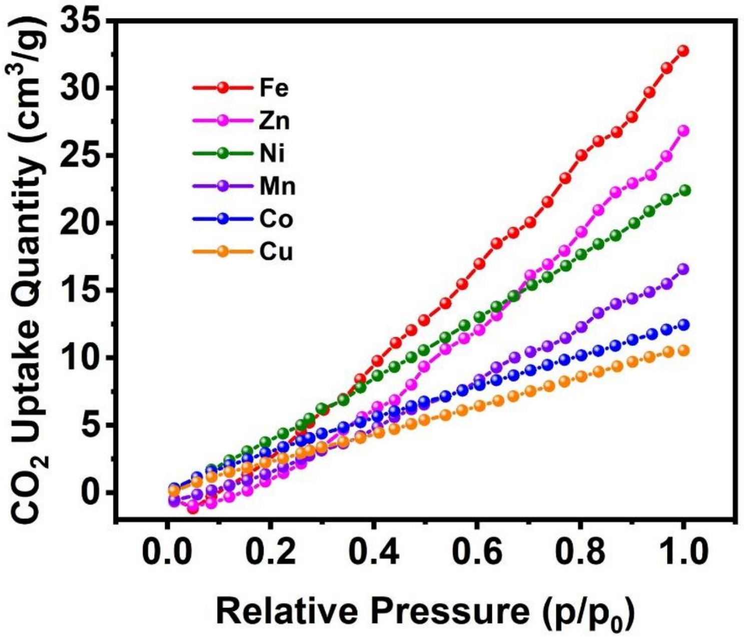

Fig. S63 Volumetric CO_2 uptake at 298 K for M-POMOFs. At 1 atm and 298 K, the CO_2 uptakes can reach 33 cm^3 g^{-1}(Fe), 27 cm^3 g^{-1}(Zn), 22 cm^3 g^{-1}(Ni), 17 cm^3 g^{-1}(Mn), 12.4 cm^3 g^{-1}(Co), 10.5 cm^3 g^{-1}(Cu), respectively.

Table S1. Comparisons of POMOFs with MOFs in CO_2 photoreduction system.

Photocatalysts	Light	Reaction time	Gas product	Yield (μmol g^{-1})	References
Fe-POMOF	λ > 420 nm	6 h	CH_4<br>CO<br>H_2	922<br>19<br>8	This work
Ni-POMOF	λ > 420 nm	6 h	CH_4<br>CO<br>H_2	29.9<br>12.9<br>14.7	This work
Cu-POMOF	λ > 420 nm	6 h	CH_4<br>CO<br>H_2	3.8<br>11.7<br>11.3	This work
Co-POMOF	λ > 420 nm	6 h	CH_4<br>CO<br>H_2	3<br>18.7<br>12.2	This work
Mn-POMOF	λ > 420 nm	6 h	CH_4<br>CO<br>H_2	2.9<br>28.4<br>32	This work
Zn-POMOF (NNU-13)	λ > 420 nm	6 h	CH_4<br>CO	704<br>25	(*29*)
NNU-14	λ > 420 nm	7 h	CH_4<br>CO	311<br>12	(*29*)
$Cu_3(BTC)_2$@TiO_2	λ < 400 nm	4 h	CH_4	11	(*38*)
TiO_2-Mg-CPO-27	λ = 365 nm	10 h	CH_4<br>CO	40.9<br>23.5	*(39)*
MOF-525	λ > 400 nm	6 h	CH_4<br>CO	37<br>384	*(40)*
MOF-525-Zn	λ > 400 nm	6 h	CH_4<br>CO	70<br>670	
MOF-525-Co	λ > 400 nm	6 h	CH_4<br>CO	221<br>1204	
ZrPP-1-Co	λ > 420 nm	15 h	CH_4<br>CO	8<br>210	*(24)*

NJU-Bai61	full light spectrum	4 h	CH_4<br>CO<br>H_2	63<br>16<br>7.48	*(42)*
MAPbI3@PCN-221($Fe_{0.2}$)	λ = 400 nm	25	CH_4<br>CO	325<br>104	*(41)*
PCN-601	λ ≥ 410 nm	10 h	CH_4<br>CO	101<br>60	*(28)*
PCN-222	λ ≥ 410 nm	10 h	CH_4<br>CO	35<br>55	*(28)*

Table S2. Conditional exploration for Fe-POMOF in photoreduction reaction of CO_2.

Photocatalyst	Injection gas	Illumination	TEOA	CH_4 (μmol g^{-1})	CO (μmol g^{-1})	H_2 (μmol g^{-1})
Fe-POMOF	CO_2	6 h	2 ml	922	19	8
no	CO_2	6 h	2 ml	not detectable	not detectable	not detectable
Fe-POMOF	Ar	6 h	2 ml	not detectable	not detectable	394
Fe-POMOF	CO_2	no	2 ml	not detectable	not detectable	not detectable
Fe-POMOF	CO_2	6 h	no	22	8	not detectable

Reaction conditions: 1. Fe-POMOF (5 mg), solvent (H_2O:28ml), TEOA (2 mL), CO_2 (1 atm), $\lambda > 420$ nm, illumination time (6h). 2. Without Fe-POMOF. 3. CO_2 was replaced by Ar. 4. In the dark. 5. Without TEOA .

Table S3. EXAFS fitting parameters at the Mo K-edge of the catalyst before and after PCR reaction (S_0^2=0.98).

	shell	CN	R(Å)	σ²	ΔE₀	*R* factor
Mo foil	Mo-Mo	8*	2.71±0.01	0.0039	3.8±1.3	0.0073
	Mo-Mo	6*	3.13±0.01	0.0032		
MoO_2	Mo-O	5.1±0.3	2.01±0.01	0.0035	1.8±1.7	0.0153
	Mo-Mo	1.2±0.3	2.54±0.02	0.0054		
MoO_3	Mo-O	2.7±1.0	1.67±0.02	0.0065	-9.6±3.4	0.0206
	Mo-O	2.0±1.5	1.94±0.03	0.0024		
	Mo-Mo	5.5±0.9	3.67±0.02	0.0050		
Mo Before	Mo-O	2.4±0.4	1.75±0.03	0.0073	7.7±4.3	0.0193
	Mo-O1	1.9±0.3	1.98±0.03	0.0018		
	Mo-Mo	0.7±0.4	2.72±0.05	0.0066		
Mo After	Mo-O	1.9±0.3	1.73±0.03	0.0035	7.9±4.0	0.0189
	Mo-O1	1.8±0.2	1.97±0.03	0.0005		
	Mo-Mo	0.9±0.4	2.72±0.05	0.0082		

[a]*CN*: coordination numbers; [b]*R*: bond distance; [c]σ^2: Debye-Waller factors; [d] ΔE_0: the inner potential correction.

The obtained XAFS data was processed in Athena (version 0.9.26) for background, pre-edge line and post-edge line calibrations. Then Fourier transformed fitting was carried out in Artemis (version 0.9.26). The k^2 weighting, k-range of 3-12 $Å^{-1}$ and R range of 1~3 Å were used for the fitting of Mo foil; k-range of 3-10 $Å^{-1}$ and R range of 1~3 Å were used for the fitting of the sample. The four parameters, coordination number, bond length, Debye-Waller factor and E_0 shift (CN, R, ΔE_0) were fitted without being fixed, the σ^2 was set.

For Wavelet Transform analysis, the χ(k) exported from Athena was imported into the Hama Fortran code. The parameters were listed as follow: R range, 1-4 Å, k range, 0-15 $Å^{-1}$ for samples; k weight, 2; and Morlet function with κ=10, σ=1 was used as the mother wavelet to provide the overall distribution.

Table S4.EXAFS fitting parameters at the Zn K-edge of the catalyst before and after PCR reaction （S_0^2=0.72）

	shell	CN	R(Å)	σ^2	ΔE_0	R factor
Zn foil	Zn-Zn	6*	2.64±0.01	0.0106	1.97±0.2	0.0074
	Zn-Zn	6*	2.75±0.07	0.0240		
ZnO	Zn-O	4.2±0.5	1.96±0.02	0.0050	3.16±2.3	0.0195
	Zn-Zn	12.8±2.2	3.23±0.02	0.0105		
Zn Before	Zn-O	4.6±0.2	1.99±0.01	0.0085	-0.4±1.4	0.0197
Zn After	Zn-O	4.7±0.3	1.99±0.01	0.0085	-0.9±1.4	0.0178

^{a}*CN*: coordination numbers; ^{b}*R*: bond distance; $^c\sigma^2$: Debye-Waller factors; ^{d} ΔE_0: the inner potential correction.

The obtained XAFS data was processed in Athena (version 0.9.26) for background, pre-edge line and post-edge line calibrations. Then Fourier transformed fitting was carried out in Artemis (version 0.9.26). The k^2 weighting, k-range of 3-12 $Å^{-1}$ and R range of 1~3 Å were used for the fitting of Zn foil; k-range of 3-10 $Å^{-1}$ and R range of 1~2 Å were used for the fitting of sample. The four parameters, coordination number, bond length, Debye-Waller factor and E_0 shift (CN, R, ΔE_0) were fitted without anyone was fixed, the σ^2 was set.

For Wavelet Transform analysis, the $\chi(k)$ exported from Athena was imported into the Hama Fortran code. The parameters were listed as follow: R range, 1-4 Å, k range, 0-15 $Å^{-1}$ for samples; k weight, 2; and Morlet function with κ=10, σ=1 was used as the mother wavelet to provide the overall distribution.

Table S5. The comparisons of current densities between M-POMOFs and MOFs in ECR reaction.

Electrocatalysts	current density (–mA cm^{-2})	Potential (V)	Electrolyte	substrate	Ref.
Fe-POMOF	30.1	–1.2 RHE	0.5 M $KHCO_3$	CP	This work
Co-POMOF	29.2	–1.2 RHE	0.5 M $KHCO_3$	CP	This work
Ni-POMOF	27.1	–1.2 RHE	0.5 M $KHCO_3$	CP	This work
Cu-POMOF	25.0	–1.2 RHE	0.5 M $KHCO_3$	CP	This work
Mn-POMOF	20.2	–1.2 RHE	0.5 M $KHCO_3$	CP	This work
Zn-POMOF	21.0	–1.2 RHE	0.5 M $KHCO_3$	CP	This work
ZIF-A-LD	~6	–1.2 RHE	0.1 M $KHCO_3$	CP	(*16*)
2D Ni-ZIF	9.0	–1.2 RHE	0.5 M $KHCO_3$	CP	(*55*)
ZIF-108	~9	–1.2 RHE	0.25 M K_2SO_4	CP	(*56*)
SIM-1	~10	–1.2 RHE	0.25 M K_2SO_4	CP	(*56*)
ZIF-7	~5	–1.2 RHE	0.25 M K_2SO_4	CP	(*56*)
ZIF-8	~11	–1.2 RHE	0.25 M K_2SO_4	CP	(*56*)
CR-MOF	~10	–1.2 SHE	0.5 M $KHCO_3$	CP	(*57*)
Cu-BTC	15-20	–1.2 SHE	0.5 M $KHCO_3$	CP	(*58*)
Cu_2O/Cu-MOF	~6	–1.2 RHE	0.1 M $KHCO_3$	GC	(*59*)
Cu@NU-1000	1.2	–0.82 RHE	0.1 M $NaClO_4$	FTO	(*60*)
ZIF-7	~20	–1.2 RHE	0.25 M K_2SO_4	CP	(*61*)
Ag@Al-PMOF	~1	–1.2 RHE	0.1 M $KHCO_3$	GC plates	(*62*)
Zn-ReSURMOF	2.5	–1.6 NHE	0.1 M TBAH/CH_3CN	FTO	(*63*)
NNU-15 (Co-MOF)	32.2	–1.1 V RHE	0.5 M $KHCO_3$	CP	(*12*)
PCN-222(Fe)	~34	–1.2 RHE	0.5 M $KHCO_3$	CP	(*64*)
Fe-MOF-525	4.0	–1.3 NHE	1.0 M $TBATF_6$/ CH_3CN	FTO	(*19*)

Table S6. Comparisons of Fe- and Co-POMOF and many MOFs in ECR performance.

Electrocatalysts	Main product	FE (%)	Potential (V)	Electrolyte	substrate	Ref.
Fe-POMOF	CO	92.1	–0.7 RHE	0.5 M $KHCO_3$	CP	This work
Co-POMOF	CO	99	–1.0 RHE	0.5 M $KHCO_3$	CP	This work
CR-MOF	HCOOH	30	–1.2 SHE	0.5 M $KHCO_3$	CP	(*57*)
Cu-BTC	Oxalic acid	51	–2.2 SHE	0.01 M TBATFB/DMF	GC	(*65*)
Cu-BTC	ethanol	10.3	–0.7 SHE	0.5 M $KHCO_3$	CP	(*58*)
CuAdeAce	MeOH	1.2	–1.55 SHE	0.5 M $KHCO_3$	CP	(*58*)
HKUST-1	CH_4	27	–1.16 RHE	0.5 M $KHCO_3$	GC	(*66*)
$Al_2(OH)_2$TCPP-Co	CO	76	–0.7 RHE	0.5 M K_2CO_3	Carbon disk	(*23*)
Fe-MOF-525	CO	50	–1.3 NHE	1.0 M $TBATF_6$/ CH_3CN	FTO	(*19*)
PCN-222(Fe)	CO	91	–0.6 RHE	0.5 M $KHCO_3$	CP	(*64*)
Cu_2(Cu-TCPP)	$HCOO^-$	68.4	–1.55 Ag/AgCl	0.5 M $EminBF_4$/ CH_3CN	FTO	(*67*)
NNU-15	CO	99.2	–0.6 V RHE	0.5 M $KHCO_3$	CP	(*12*)
Zn-BTC	CH_4	80	–2.0 SHE	$BmimBF_4$	CP	(*68*)
ZIF-7	CO	23.8	–1.1 RHE	0.25 M K_2SO_4	CP	(*56*)
ZIF-8	CO	81	–1.1 RHE	0.25 M K_2SO_4	CP	(*56*)
ZIF-108	CO	63.5	–1.1 RHE	0.25 M K_2SO_4	CP	(*56*)
SIM-1	CO	66.6	–1.1 RHE	0.25 M K_2SO_4	CP	(*56*)
ZIF-A-LD	CO	90.57	–1.1 RHE	0.1 M $KHCO_3$	CP	(*16*)
Cu@NU-1000	$HCOO^-$	28	–0.82 RHE	0.1 M $NaClO_4$	FTO	(*60*)
Cu_2O/Cu-MOF	CH_4	63.2	–1.71 RHE	0.1 M $KHCO_3$	GC	(*59*)
Ag_2O/ZIF-L	CO	80.5	–1.2 RHE	0.25 M K_2SO_4	CP	(*61*)
Ag@Al-PMOF	CO	55.8	–1.1 RHE	0.1 M $KHCO_3$	GC	(*62*)
Enzyme FDH@NU-1006	HCOOH	–	–1.1 Ag/AgCl	0.5 M Tris-buffered solution	FTO	(*69*)
Zn-ReSURMOF	CO	93	–1.6 NHE	0.1 M TBAH/CH_3CN	FTO	(*63*)
2D Ni-ZIF	CO	78.8	–0.85 RHE	0.5 M $KHCO_3$	CP	(*55*)

Table S7. The correction from the zero-point energy, entropy and heat capacity for converting the total energies to Gibbs free energies (units: eV).

Species	ZPE	TS	$\int C_p dT$
*COOH	0.63	0.17	0.09
H*COOH	0.82	0.085	0.049
H*C(OH)2	1.19	0.13	0.071
*CO	0.22	0.08	0.05
H_2O	0.58	0.42	0.09
CH_4	1.2	0.6	0.10
*HCOH	0.76	0.068	0.11
*CH	0.35	0.039	0.028
*CH_2	0.59	0.075	0.049
*CH_3	0.9	0.096	0.06

REFERENCES AND NOTES

1. R. G. Grim, Z. Huang, M. T. Guarnieri, J. R. Ferrell III, L. Tao, J. A. Schaidle, Transforming the carbon economy: Challenges and opportunities in the convergence of low-cost electricity and reductive CO_2 utilization. *Energ. Environ. Sci.* **13**, 472–494 (2020).

2. Y. Zhang, L.-Z. Dong, S. Li, X. Huang, J.-N. Chang, J.-H. Wang, J. Zhou, S.-L. Li, Y.-Q. Lan, Coordination environment dependent selectivity of single-site-Cu enriched crystalline porous catalysts in CO_2 reduction to CH_4. *Nat. Commun.* **12**, 6390 (2021).

3. N. Mohd Adli, W. Shan, S. Hwang, W. Samarakoon, S. Karakalos, Y. Li, D. A. Cullen, D. Su, Z. Feng, G. Wang, G. Wu, Engineering atomically dispersed FeN_4 active sites for CO_2 electroreduction. *Angew. Chem. Int. Ed.* **60**, 1022–1032 (2021).

4. D. Yao, C. Tang, A. Vasileff, X. Zhi, Y. Jiao, S.-Z. Qiao, The controllable reconstruction of Bi-MOFs for electrochemical CO_2 reduction through electrolyte and potential mediation. *Angew. Chem. Int. Ed.* **60**, 18178–18184 (2021).

5. X. Li, L. Liu, X. Ren, J. Gao, Y. Huang, B. Liu, Microenvironment modulation of single-atom catalysts and their roles in electrochemical energy conversion. *Science Advances* **6**, eabb6833 (2020).

6. D. Gao, Y. Zhang, Z. Zhou, F. Cai, X. Zhao, W. Huang, Y. Li, J. Zhu, P. Liu, F. Yang, G. Wang, X. Bao, Enhancing CO2 electroreduction with the metal-oxide interface. *J. Am. Chem. Soc.* **139**, 5652–5655 (2017).

7. X. Su, X.-F. Yang, Y. Huang, B. Liu, T. Zhang, Single-atom catalysis toward efficient CO_2 conversion to CO and formate products. *Acc. Chem. Res.* **52**, 656–664 (2019).

8. Y. Wang, P. Han, X. Lv, L. Zhang, G. Zheng, Defect and interface engineering for aqueous electrocatalytic CO_2 reduction. *Joule* **2**, 2551–2582 (2018).

9. A. Kirchon, L. Feng, H. F. Drake, E. A. Joseph, H.-C. Zhou, From fundamentals to applications: A toolbox for robust and multifunctional MOF materials. *Chem. Soc. Rev.* **47**,

8611–8638 (2018).

10. O. K. Farha, I. Eryazici, N. C. Jeong, B. G. Hauser, C. E. Wilmer, A. A. Sarjeant, R. Q. Snurr, S. T. Nguyen, A. O. Yazaydin, J. T. Hupp, Metal-organic framework materials with ultrahigh surface areas: Is the sky the limit? *J. Am. Chem. Soc.* **134**, 15016–15021 (2012).

11. L. W. Chen, Y. C. Hao, Y. Guo, Q. Zhang, J. Li, W. Y. Gao, L. Ren, X. Su, L. Hu, N. Zhang, S. Li, X. Feng, L. Gu, Y. W. Zhang, A. X. Yin, B. Wang, Metal-organic framework membranes encapsulating gold nanoparticles for direct plasmonic photocatalytic nitrogen fixation. *J. Am. Chem. Soc.* **143**, 5727–5736 (2021).

12. Q. Huang, Q. Li, J. Liu, Y. R. Wang, R. Wang, L. Z. Dong, Y. H. Xia, J. L. Wang, Y.-Q. Lan, Disclosing CO_2 activation mechanism by hydroxyl-induced crystalline structure transformation in electrocatalytic process. *Matter* **1**, 1656–1668 (2019).

13. Z. Liang, C. Qu, W. Guo, R. Zou, Q. Xu, Pristine metal-organic frameworks and their composites for energy storage and conversion. *Adv. Mater.* **30**, 1702891 (2018).

14. X. Zhou, J. Shan, L. Chen, B. Y. Xia, T. Ling, J. Duan, Y. Jiao, Y. Zheng, S.-Z. Qiao, Stabilizing Cu^{2+} ions by solid solutions to promote CO_2 electro reduction to methane. *J. Am. Chem. Soc.* **144**, 2079–2084 (2022).

15. H. Zhang, J. Wei, J. Dong, G. Liu, L. Shi, P. An, G. Zhao, J. Kong, X. Wang, X. Meng, J. Zhang, J. Ye, Efficient visible-light-driven carbon dioxide reduction by a single-atom implanted metal-organic framework. *Angew. Chem.-Int. Edit.* **55**, 14308–14312 (2016).

16. S. Dou, J. Song, S. Xi, Y. Du, J. Wang, Z.-F. Huang, Z. J. Xu, X. Wang, Boosting electrochemical CO_2 reduction on metal-organic frameworks via ligand doping. *Angew. Chem. Int. Ed.* **58**, 4041–4045 (2019).

17. Y. Wang, N. Y. Huang, J. Q. Shen, P. Q. Liao, X. M. Chen, J. P. Zhang, Hydroxide ligands cooperate with catalytic centers in metal-organic frameworks for efficient photocatalytic CO_2 reduction. *J. Am. Chem. Soc.* **140**, 38–41 (2018).

18. Y. C. Hao, L. W. Chen, J. Li, Y. Guo, X. Su, M. Shu, Q. Zhang, W. Y. Gao, S. Li, Z. L. Yu, L. Gu, X. Feng, A. X. Yin, R. Si, Y. W. Zhang, B. Wang, C. H. Yan, Metal-organic framework membranes with single-atomic centers for photocatalytic CO_2 and O_2 reduction. *Nat. Commun.* **12**, 2682 (2021).

19. I. Hod, M. D. Sampson, P. Deria, C. P. Kubiak, O. K. Farha, J. T. Hupp, Fe-porphyrin-based metal-organic framework films as high-surface concentration, heterogeneous catalysts for electrochemical reduction of CO_2. *Acs Catalysis* **5**, 6302–6309 (2015).

20. Z. Liang, H.-Y. Wang, H. Zheng, W. Zhang, R. Cao, Porphyrin-based frameworks for oxygen electrocatalysis and catalytic reduction of carbon dioxide. *Chem. Soc. Rev.* **50**, 2540–2581 (2021).

21. H.-Q. Xu, J. Hu, D. Wang, Z. Li, Q. Zhang, Y. Luo, S.-H. Yu, H.-L. Jiang, Visible-light photoreduction of CO_2 in a metal-organic framework: Boosting electron-hole separation via electron trap states. *J. Am. Chem. Soc.* **137**, 13440–13443 (2015).

22. X.-J. Kong, T. He, J. Zhou, C. Zhao, T.-C. Li, X.-Q. Wu, K. Wang, J.-R. Li, In situ porphyrin substitution in a Zr(IV)-MOF for stability enhancement and photocatalytic CO_2 reduction. *Small* **17**, 2005357 (2021).

23. N. Kornienko, Y. Zhao, C. S. Kley, C. Zhu, D. Kim, S. Lin, C. J. Chang, O. M. Yaghi, P. Yang, Metal-organic frameworks for electrocatalytic reduction of carbon dioxide. *J. Am. Chem. Soc.* **137**, 14129–14135 (2015).

24. E.-X. Chen, M. Qiu, Y.-F. Zhang, Y.-S. Zhu, L.-Y. Liu, Y.-Y. Sun, X. Bu, J. Zhang, Q. Lin, Acid and base resistant zirconium polyphenolate-metalloporphyrin scaffolds for efficient CO_2 photoreduction. *Adv. Mater.* **30**, 1704388 (2018).

25. Y.-C. Qiu, S. Yuan, X.-X. Li, D.-Y. Du, C. Wang, J.-S. Qin, H. F. Drake, Y.-Q. Lan, L. Jiang, H.-C. Zhou, Face-sharing Archimedean solids stacking for the construction of mixed-ligand metal-organic frameworks. *J. Am. Chem. Soc.* **141**, 13841–13848 (2019).

26. Y. Fu, D. Sun, Y. Chen, R. Huang, Z. Ding, X. Fu, Z. Li, An amine-functionalized titanium

metal-organic framework photocatalyst with visible-light-induced activity for CO_2 reduction. *Angew. Chem.-Int. Edit.* **51**, 3364–3367 (2012).

27. Z.-H. Yan, M.-H. Du, J. Liu, S. Jin, C. Wang, G.-L. Zhuang, X.-J. Kong, L.-S. Long, L.-S. Zheng, Photo-generated dinuclear $\{Eu(II)\}_{(2)}$ active sites for selective CO_2 reduction in a photosensitizing metal-organic framework. *Nat. Commun.* **9**, 3353 (2018).

28. Z.-B. Fang, T.-T. Liu, J. Liu, S. Jin, X.-P. Wu, X.-Q. Gong, K. Wang, Q. Yin, T.-F. Liu, R. Cao, H.-C. Zhou, Boosting interfacial charge-transfer kinetics for efficient overall CO_2 photoreduction via rational design of coordination spheres on metal-organic frameworks. *J. Am. Chem. Soc.* **142**, 12515–12523 (2020).

29. Q. Huang, J. Liu, L. Feng, Q. Wang, W. Guan, L.-Z. Dong, L. Zhang, L.-K. Yang, Y.-Q. Lan, H.-C. Zhou, Multielectron transportation of polyoxometalate-grafted metalloporphyrin coordination frameworks for selective CO_2-to-CH_4 photoconversion. *Natl. Sci. Rev.* **7**, 53–63 (2020).

30. H. Wang, S. Hamanaka, Y. Nishimoto, S. Irle, T. Yokoyama, H. Yoshikawa, K. Awaga, In operando x-ray absorption fine structure studies of polyoxometalate molecular cluster batteries: Polyoxometalates as electron sponges. *J. Am. Chem. Soc.* **134**, 4918–4924 (2012).

31. N. I. Gumerova, A. Rompel, Synthesis, structures and applications of electron-rich polyoxometalates. *Nat. Rev. Chem.* **2**, 0112 (2018).

32. N. I. Gumerova, A. Rompel, Polyoxometalates in solution: Speciation under spotlight. *Chem. Soc. Rev.* **49**, 7568–7601 (2020).

33. B. Nohra, H. El Moll, L. M. Rodriguez Albelo, P. Mialane, J. Marrot, C. Mellot-Draznieks, M. O’Keeffe, R. Ngo Biboum, J. Lemaire, B. Keita, L. Nadjo, A. Dolbecq, Polyoxometalate-based metal organic frameworks (POMOFs): Structural trends, energetics, and high electrocatalytic efficiency for hydrogen evolution reaction. *J. Am. Chem. Soc.* **133**, 13363–13374 (2011).

34. C. Zou, Z. Zhang, X. Xu, Q. Gong, J. Li, C.-D. Wu, A multifunctional organic-inorganic

hybrid structure based on MnIII-porphyrin and polyoxometalate as a highly effective dye scavenger and heterogenous catalyst. *J. Am. Chem. Soc.* **134**, 87–90 (2012).

35. D.-Y. Du, J.-S. Qin, S.-L. Li, Z.-M. Su, Y.-Q. Lan, Recent advances in porous polyoxometalate-based metal-organic framework materials. *Chem. Soc. Rev.* **43**, 4615–4632 (2014).

36. J.-S. Qin, D.-Y. Du, W. Guan, X.-J. Bo, Y.-F. Li, L.-P. Guo, Z.-M. Su, Y.-Y. Wang, Y.-Q. Lan, H.-C. Zhou, Ultrastable polymolybdate-based metal-organic frameworks as highly active electrocatalysts for hydrogen generation from water. *J. Am. Chem. Soc.* **137**, 7169–7177 (2015).

37. L. Marleny Rodriguez-Albelo, A. R. Ruiz-Salvador, A. Sampieri, D. W. Lewis, A. Gómez, B. Nohra, P. Mialane, J. Marrot, F. Sécheresse, C. Mellot-Draznieks, R. Ngo Biboum, B. Keita, L. Nadjo, A. Dolbecq, Zeolitic polyoxometalate-based meta-organic frameworks (Z-POMOFs): Computational evaluation of hypothetical polymorphs and the successful targeted synthesis of the redox-active Z-POMOF1. *J. Am. Chem. Soc.* **131**, 16078–16087 (2009).

38. R. Li, J. Hu, M. Deng, H. Wang, X. Wang, Y. Hu, H.-L. Jiang, J. Jiang, Q. Zhang, Y. Xie, Y. Xiong, Integration of an inorganic semiconductor with a metal-organic framework: A platform for enhanced gaseous photocatalytic reactions. *Adv. Mater.* **26**, 4783–4788 (2014).

39. M. Wang, D. Wang, Z. Li, Self-assembly of CPO-27-Mg/TiO_2 nanocomposite with enhanced performance for photocatalytic CO_2 reduction. *Appl. Catal. Environ.* **183**, 47–52 (2016).

40. H. Zhang, J. Wei, J. Dong, G. Liu, L. Shi, P. An, G. Zhao, J. Kong, X. Wang, X. Meng, J. Zhang, J. Ye, Efficient visible-light-driven carbon dioxide reduction by a single-atom implanted metal-organic framework. *Angew. Chem. Int. Ed.* **55**, 14310–14314 (2016).

41. L.-Y. Wu, Y.-F. Mu, X.-X. Guo, W. Zhang, Z.-M. Zhang, M. Zhang, T.-B. Lu, Encapsulating perovskite quantum dots in iron-based metal-organic frameworks (MOFs) for efficient photocatalytic CO_2 reduction. *Angew. Chem. Int. Ed.* **58**, 9491–9495 (2019).

42. Y. Gao, L. Zhang, Y. Gu, W. Zhang, Y. Pan, W. Fang, J. Ma, Y.-Q. Lan, J. Bai, Formation of

a mixed-valence Cu(i)/Cu(ii) metal-organic framework with the full light spectrum and high selectivity of CO_2 photo reduction into CH_4. *Chem. Sci.* **11**, 10143–10148 (2020).

43. S. Wang, M. Xu, T. Peng, C. Zhang, T. Li, I. Hussain, J. Wang, B. Tan, Porous hypercrosslinked polymer-TiO_2-graphene composite photocatalysts for visible-light-driven CO_2 conversion. *Nat. Commun.* **10**, 676 (2019).

44. A. Cadiau, N. Kolobov, S. Srinivasan, M. G. Goesten, H. Haspel, A. V. Bavykina, M. R. Tchalala, P. Maity, A. Goryachev, A. S. Poryvaev, M. Eddaoudi, M. V. Fedin, O. F. Mohammed, J. Gascon, A titanium metal-organic framework with visible-light-responsive photocatalytic activity. *Angew. Chem. Int. Ed.* **59**, 13468–13472 (2020).

45. D. Feng, Z.-Y. Gu, J.-R. Li, H.-L. Jiang, Z. Wei, H.-C. Zhou, Zirconium-metalloporphyrin PCN-222: Mesoporous metal-organic frameworks with ultrahigh stability as biomimetic catalysts. *Angew. Chem.-Int. Edit.* **51**, 10307–10310 (2012).

46. L. Liang, X. Li, Y. Sun, Y. Tan, X. Jiao, H. Ju, Z. Qi, J. Zhu, Y. Xie, Infrared light-driven CO_2 overall splitting at room temperature. *Joule* **2**, 1004–1016 (2018).

47. X. Li, Y. Sun, J. Xu, Y. Shao, J. Wu, X. Xu, Y. Pan, H. Ju, J. Zhu, Y. Xie, Selective visible-light-driven photocatalytic CO_2 reduction to CH_4 mediated by atomically thin $CuIn_5S_8$ layers. *Nat. Energy* **4**, 690–699 (2019).

48. L. C. Grabow, M. Mavrikakis, Mechanism of methanol synthesis on Cu through CO_2 and CO hydrogenation. *ACS Catalysis* **1**, 365–384 (2011).

49. Z. Jiang, H. Sun, T. Wang, B. Wang, W. Wei, H. Li, S. Yuan, T. An, H. Zhao, J. Yu, P. K. Wong, Nature-based catalyst for visible-light-driven photocatalytic CO_2 reduction. *Energ. Environ. Sci.* **11**, 2382–2389 (2018).

50. S. Barman, A. Singh, F. A. Rahimi, T. K. Maji, Metal-free catalysis: A redox-active donor-acceptor conjugated microporous polymer for selective visible-light-driven CO_2 reduction to CH_4. *J. Am. Chem. Soc.* **143**, 16284–16292 (2021).

51. J.-D. Yi, R. Xie, Z.-L. Xie, G.-L. Chai, T.-F. Liu, R.-P. Chen, Y.-B. Huang, R. Cao, Highly selective CO_2 electroreduction to CH_4 by in situ generated Cu_2O single-type sites on a conductive MOF: Stabilizing key intermediates with hydrogen bonding. *Angew. Chem. Int. Edit.* **59**, 23641–23648 (2020).

52. S. Zhu, B. Jiang, W.-B. Cai, M. Shao, Direct observation on reaction intermediates and the role of bicarbonate anions in CO_2 electrochemical reduction reaction on Cu surfaces. *J. Am. Chem. Soc.* **139**, 15664–15667 (2017).

53. N. J. Firet, W. A. Smith, Probing the reaction mechanism of CO_2 electroreduction over Ag films via operando infrared spectroscopy. *Acs Catalysis* **7**, 606–612 (2017).

54. Q. Huang, Q. Niu, N. N. Ma, L. Z. Dong, S. L. Li, D. S. Li, Y. P. Cai, Y. Q. Lan, Axial Cl/Br atom-mediated CO_2 electroreduction performance in a stable porphyrin-based metal-organic framework. *Chem. Commun.* **56**, 14817–14820 (2020).

55. Y. Gao, L. Zhang, Y. Gu, W. Zhang, Y. Pan, W. Fang, J. Ma, Y.-Q. Lan, J. Bai, Formation of a mixed-valence Cu(i)/Cu(ii) metal-organic framework with the full light spectrum and high selectivity of CO_2 photoreduction into CH_4. *Chem. Sci.* **11**, 10143–10148 (2020).

56. J.-X. Wu, W.-W. Yuan, M. Xu, Z.-Y. Gu, Ultrathin 2D nickel zeolitic imidazolate framework nanosheets for electrocatalytic reduction of CO_2. *Chem. Commun.* **55**, 11634–11637 (2019).

57. X. Jiang, H. Li, J. Xiao, D. Gao, R. Si, F. Yang, Y. Li, G. Wang, X. Bao, Carbon dioxide electroreduction over imidazolate ligands coordinated with Zn (II) center in ZIFs. *Nano Energy* **52**, 345–350 (2018).

58. R. Hinogami, S. Yotsuhashi, M. Deguchi, Y. Zenitani, H. Hashiba, Y. Yamada, Electrochemical reduction of carbon dioxide using a copper rubeanate metal organic framework. *Ecs Electrochem. Lett.* **1**, H17–H19 (2012).

59. J. Albo, D. Vallejo, G. Beobide, O. Castillo, P. Castano, A. Irabien, Copper-based metal-organic porous materials for CO_2 electrocatalytic reduction to alcohols. *ChemSusChem* **10**,

1100–1109 (2017).

60. X. Tan, C. Yu, C. Zhao, H. Huang, X. Yao, X. Han, W. Guo, S. Cui, H. Huang, J. Qiu, Restructuring of Cu_2O to Cu_2O@Cu-metal-organic frameworks for selective electrochemical reduction of CO2. *ACS Appl. Mater. Interfaces* **11**, 9904–9910 (2019).

61. C.-W. Kung, C. O. Audu, A. W. Peters, H. Noh, O. K. Farha, J. T. Hupp, Copper nanoparticles installed in metal-organic framework thin films are electrocatalytically competent for CO_2 reduction. *ACS Energy Lett.* **2**, 2394–2401 (2017).

62. X. Jiang, H. Wu, S. Chang, R. Si, S. Miao, W. Huang, Y. Li, G. Wang, X. Bao, Boosting CO_2 electroreduction over layered zeolitic imidazolate frameworks decorated with Ag_2O nanoparticles. *J. Mater. Chem. A* **5**, 19371–19377 (2017).

63. Y. T. Guntern, J. R. Pankhurst, J. Vávra, M. Mensi, V. Mantella, P. Schouwink, R. Buonsanti, Nanocrystal/metal-organic framework hybrids as electrocatalytic platforms for CO_2 Conversion. *Angew. Chem. Int. Ed.* **58**, 12632–12639 (2019).

64. L. Ye, J. Liu, Y. Gao, C. Gong, M. Addicoat, T. Heine, C. Woell, L. Sun, Highly oriented MOF thin film-based electrocatalytic device for the reduction of CO_2 to CO exhibiting high Faradaic efficiency. *J. Mater. Chem. A* **4**, 15320–15326 (2016).

65. B.-X. Dong, S.-L. Qian, F.-Y. Bu, Y.-C. Wu, L.-G. Feng, Y.-L. Teng, W.-L. Liu, Z.-W. Li, Electrochemical reduction of CO_2 to CO by a heterogeneous catalyst of Fe–porphyrin-based metal-organic framework. *ACS Appl. Energy Mater.* **1**, 4662–4669 (2018).

66. R. S. Kumar, S. S. Kumar, M. A. Kulandainathan, Highly selective electrochemical reduction of carbon dioxide using Cu based metal organic framework as an electrocatalyst. *Electrochem. Commun.* **25**, 70–73 (2012).

67. Z. Weng, Y. Wu, M. Wang, J. Jiang, K. Yang, S. Huo, X.-F. Wang, Q. Ma, G. W. Brudvig, V. S. Batista, Active sites of copper-complex catalytic materials for electrochemical carbon dioxide reduction. *Nat. Commun.* **9**, 415 (2018).

68. J.-X. Wu, S.-Z. Hou, X.-D. Zhang, M. Xu, H.-F. Yang, P.-S. Cao, Z.-Y. Gu, Cathodized copper porphyrin metal-organic framework nanosheets for selective formate and acetate production from CO_2 electroreduction. *Chem. Sci.* **10**, 2199–2205 (2019).

69. X. Kang, Q. Zhu, X. Sun, J. Hu, J. Zhang, Z. Liu, B. Han, Highly efficient electrochemical reduction of CO_2 to CH_4 in an ionic liquid using a metal-organic framework cathode. *Chem. Sci.* **7**, 266–273 (2016).

70. Y. Chen, P. Li, H. Noh, C. W. Kung, C. T. Buru, X. Wang, X. Zhang, O. K. Farha, Stabilization of formate dehydrogenase in a metal-organic framework for bioelectrocatalytic reduction of CO_2. *Angew. Chem. Int. Ed.* **58**, 7682–7686 (2019).
